# Disruption of maternal vascular remodeling by a fetal endoretrovirus-derived gene in preeclampsia

**DOI:** 10.1186/s13059-024-03265-z

**Published:** 2024-05-07

**Authors:** Xiaoli Gong, Wei He, Wan Jin, Hongwei Ma, Gang Wang, Jiaxin Li, Yu Xiao, Yangyu Zhao, Qiong Chen, Huanhuan Guo, Jiexia Yang, Yiming Qi, Wei Dong, Meng Fu, Xiaojuan Li, Jiusi Liu, Xinghui Liu, Aihua Yin, Yi Zhang, Yuan Wei

**Affiliations:** 1 Department of Obstetrics and Gynecology, Peking University Third Hospital, Beijing, China; 2 Medical Genetic Center, Guangdong Women and Children Hospital, Guangzhou, China; 3 Euler Technology, Beijing, China; 4 Department of Obstetrics and Gynecology, West China Second University Hospital of Sichuan University, Chengdu, China; 5 Department Key Laboratory of Birth Defects and Related Diseases of Women and Children (Sichuan University), Ministry of Education, Chengdu, China; 6 Department of Urology, Zhongnan Hospital of Wuhan University, Wuhan, China; 7 Department of Biological Repositories, Zhongnan Hospital of Wuhan University, Wuhan, China; 8 Human Genetic Resources Preservation Center of Hubei Province, Wuhan, China; 9 Laboratory of Precision Medicine, Zhongnan Hospital of Wuhan University, Wuhan, China; 10 Maternity Ward, Haidian Maternal and Child Health Hospital, Beijing, China; 11 Department of Obstetrics and Gynecology, Haidian Maternal and Child Health Hospital, Beijing, China; 12 Present Address: International Max Planck Research School for Genome Science, and University of Göttingen, Göttingen Center for Molecular Biosciences, Göttingen, Germany

## Abstract

**Background:**

Preeclampsia, one of the most lethal pregnancy-related diseases, is associated with the disruption of uterine spiral artery remodeling during placentation. However, the early molecular events leading to preeclampsia remain unknown.

**Results:**

By analyzing placentas from preeclampsia, non-preeclampsia, and twin pregnancies with selective intrauterine growth restriction, we show that the pathogenesis of preeclampsia is attributed to immature trophoblast and maldeveloped endothelial cells. Delayed epigenetic reprogramming during early extraembryonic tissue development leads to generation of excessive immature trophoblast cells. We find reduction of de novo DNA methylation in these trophoblast cells results in selective overexpression of maternally imprinted genes, including the endoretrovirus-derived gene PEG10 (paternally expressed gene 10). PEG10 forms virus-like particles, which are transferred from the trophoblast to the closely proximate endothelial cells. In normal pregnancy, only a low amount of PEG10 is transferred to maternal cells; however, in preeclampsia, excessive PEG10 disrupts maternal vascular development by inhibiting TGF-beta signaling.

**Conclusions:**

Our study reveals the intricate epigenetic mechanisms that regulate trans-generational genetic conflict and ultimately ensure proper maternal–fetal interface formation.

**Supplementary Information:**

The online version contains supplementary material available at 10.1186/s13059-024-03265-z.

## Background

Preeclampsia (PE), a pregnancy-specific disease, is characterized by endothelial dysfunction and unmanageable hypertension that leads to multi-organ damage in the expectant mother. Each year, PE affects 3–5% of pregnancies, leading to at least 42,000 maternal deaths globally [1, 2]. The curative effect of removing the placenta and fetus on PE indicates that the placenta may be the origin of all maternal syndromes. As one of the “Great Obstetrical Syndromes” [3], PE exhibits a placenta that shares pathological features with other diseases, such as fetal growth restriction (FGR) and preterm birth [3]. Anatomically, PE is characterized by incomplete development of the villous tree [4], reduced vascularization into the terminal villus [4, 5], and incomplete remodeling of maternal uterine spiral arteries [6, 7].

Population genetic studies have shown that susceptibility to PE involves a genetic component. PE is strongly associated with both the maternal and fetal genomes but less so with environmental factors [8, 9]. For the fetal genome, it was proposed that PE risk is inherited from the paternal copy [9]. Genome-wide association scans (GWAS) have revealed that PE risk is associated with various loci that are implicated in maternal hypertension susceptibility, endothelial cell development, and the expression of the fetal vascular epidermal growth factor (VEGF) receptor gene fms-related receptor tyrosine kinase 1 (*FLT1)* [10–16], suggesting that the genetic factors influencing PE center on vascular development and function. A genetic association of PE to endothelial development genes in the maternal genome [15] and loci neighboring FLT1 in the fetal genome [13] suggests that PE results from the interaction between fetal and maternal cells.

Biochemically, PE is associated with elevated levels of angiotensinogen (AGT) [17–19], s-FLT1 [20–22], and soluble endoglin (s-Eng) [21, 23, 24], as well as reduced levels of PAPPA [25] and PlGF [21, 26–28] in the maternal serum, which collectively result in maternal peripheral vasoconstriction [29], decreased arterial compliance [30], and organ damage such as glomerular endotheliosis [31, 32]. Studies involving animal models [33, 34] and humans [6, 35] have revealed that these biochemical changes are attributed to trophoblast cells that invade the maternal uterus myometrium, suggesting that the interactions between trophoblast and endothelial cells may be central to PE pathogenesis.

Clinical, genetic, and anatomical data provide compelling evidence that the primary PE syndrome is due to a developmental defect in the maternal–fetal interface, i.e., the placenta. As an organ of the extraembryonic lineage, the placenta originates from the trophectoderm of the blastocyst. The specification and differentiation of placental cells are driven by genome-wide epigenomic reprogramming [36–39]. This overall epigenomic reprogramming during placental development is pivotal for proper placentation. This phenomenon not only establishes the proper cellular composition of the placenta but also regulates trans-generational conflict by modulating the gene expression levels that differentially affect maternal and fetal health. Shortly after zygote formation, global erasure of DNA methylation and H3K27me3 repressive marks results in a genome that is permissive for transcriptional activation during the zygote genome activation (ZGA) stage [40–43]. The reinstatement of repressive epigenetic marks on lineage specification genes during subsequent development is instigated by the priming of maternal and ZGA-active transcription factor (TF) [44–46], the binding of polycomb group protein [47–49], and the de novo DNA methylation of CpG-island promoters [50]. Recent studies have shown that genomic DNA from PE placentas tend to be hypomethylated compared with that from non-PE placentas [51–53], suggesting the occurrence of defects in DNA methylation during placenta development in PE. However, the precise molecular mechanism underlying the epigenetic and developmental alterations in PE remains unclear.

In this study, through multi-omic integrative analysis of placentas from PE, non-PE, and twin pregnancies with selective intrauterine growth restriction, we revealed complex epigenetic processes regulating trans-generational genetic conflict to ensure proper maternal–fetal interface formation. Our results showed how disruption of such processes results in PE.

## Results

### Identification of pathogenic cells in PE

**Fig. 1:**
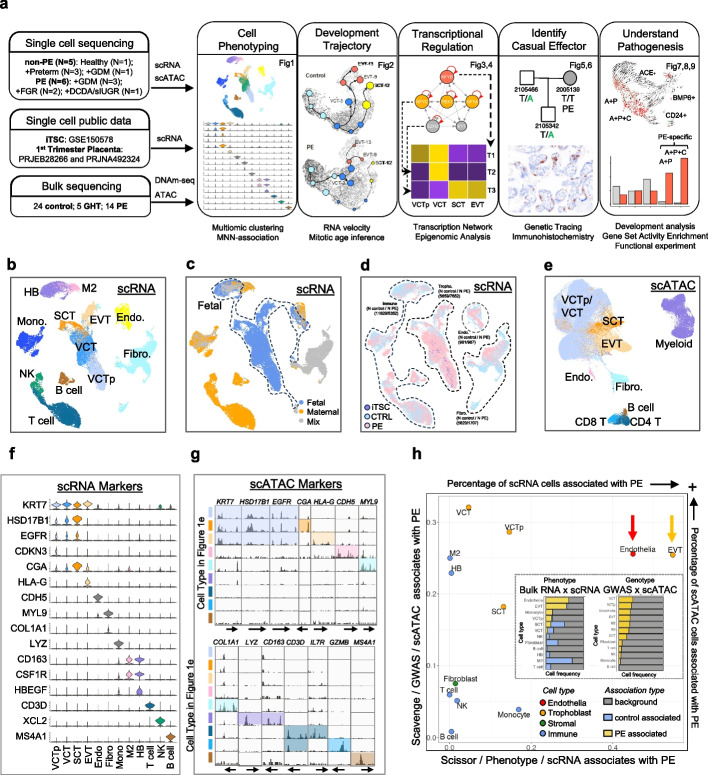
Pathogenic cells underlying preeclampsia. **a** Schematic overview of multi-omic study for PE pathogenic mechanism. 10 × Genomics single-cell RNA and ATAC sequencing were performed with 11 donors (normal pregnancy: 1; GDM: 1; preterm birth: 3; PE and GDM (PE-GDM): 2; PE and FGR (PE-FGR): 3; DCDA sIUGR: 1). Additionally, we supplemented this dataset with those of early-stage cells, i.e., public scRNA datasets pertaining to first-trimester placentas (PRJEB28266 and PRJNA492324) [54–57] and induced trophoblast stem cells (iTSCs, GSE150578 [58, 59]). Bulk genome-wide CpG capture bisulfite sequencing (DNAm-Seq) and ATAC sequencing were performed with 43 placenta samples (including 24 control, 5 GHT, 14 PE). By integration of single-cell and bulk sequencing, we annotated the major cell types in placenta; identified that trophoblast and endothelial cells were the most PE-associated cell types; reconstructed the developmental trajectory of trophoblast; revealed master transcriptional factors during trophoblast development, uncovered causal effector for PE pathological phenotype by genetic tracing and immunohistochemistry, and identified their downstream target cell. **b** UMAP projection of major cell types in scRNA cells, including trophoblasts. **c** UMAP projection of cell origin in scRNA. Trophoblast (including VCTp, VCT, SCT, and EVT) and Hoffbauer cell (HB) are fetal originated (blue); immune cells (including monocyte, M2, NK, T, and B cell) are from the maternal (orange); endothelial and fibroblast/stromal cells are contributed by both fetal and maternal. **d** UMAP projection of phenotype in scRNA. Cells of control and PE placentas are intermingled in each cell type. iTSC cells from GSE150578 [58, 59] are majorly integrated with trophoblast progenitors (VCTp). The cell number of each type in control and PE was labeled. **e** UMAP projection of scATAC cells. VCT/VCTp cells are mixed in this modality, while CD4 and CD8 T cells are clearly segregated. **f** Known marker gene expression of scRNA cell clusters. **g** Chromatin accessibility on marker genes of scATAC cell clusters. Color bar on the left side indicates the cell type in Fig. 1e. **h** PE-associated cell types identified by both phenotype-gene expression association and GWAS-chromatin accessibility association. Large panel: prevalence of Scissor-inferred PE-associated cells with scRNA (*x* axis) and the prevalence of SCAVENGE-inferred PE-associated cells with scATAC (*y*-axis). VCTp/VCT clusters are associated with PE in terms of hereditary risk but not gene expression, suggesting their implication in PE might due to abnormal differentiation but not their own function. In contrast, both endothelial cells and EVT are associated with PE hereditary risk loci as well as gene expression, implying the direct pathogenic role of them in PE. Small panel: (left) Scissor-inferred single-cell association with RNA expression profile from control or PE placenta, showing that PE is mostly associated with endothelial cells, EVT, SCT, VCTp, and monocytes. (right) SCAVENGE-inferred scATAC association with PE-associated GWAS loci, showing that PE GWAS loci are associated with VCT, VCTp, EVT, SCT, macrophages, and endothelial cells. *Abbreviation*: DNAm-seq: genome-wide CpG capture bisulfite sequencing; GHT: gestational hypertension; VCTp: trophoblast progenitor; VCT: villous trophoblast; SCT: synciotrophoblast; EVT: extravillous trophoblast; Mono: monocyte; NK: natural killer cell; M2: type-2 macrophage; HB: Hoffbauer cell; Fibro: fibroblasts/stromal cells; Endo: endothelial cells

We sequenced single cells and bulk tissues of the placenta from both non-PE and PE pregnancies (Fig. 1a). Using 10 × Genomics single-cell RNA (scRNA) and single-cell chromatin accessibility (scATAC), we sequenced fetal and maternal surfaces of placentas obtained from 11 donors, including those with normal pregnancy (*N* = 2/1/1 [sample/placenta/donor]), gestational diabetes mellitus (GDM) pregnancy (*N* = 2/1/1), preterm birth (*N* = 5/3/3), PE and GDM (PE-GDM) pregnancy (*N* = 6/3/3), and PE and FGR (PE-FGR) pregnancy (*N* = 4/2/2), and a pair of placentas from a dizygotic twin pregnancy with selective intrauterine growth restriction (DCDA sIUGR, *N* = 2/2/1, only fetal surface available) (“Methods,” Fig. 1a,b, Additional file 1: Fig. S1a-c; Additional file 2: Tables S1 and Additional file 3: Table S2). Two (2) of these donors, a non-PE control donor (2100042) and a DCDA sIUGR donor (2200314) (Additional file 2: Table S1), were not enrolled in the first study period (before 2021) but enrolled latter (after 2021) for internal validation. Hence, in Fig. 1b–h, we only used 9 donors out of 11. Additionally, we supplemented this dataset with those of early-stage cells, i.e., public scRNA datasets pertaining to first-trimester placentas [54–57] and induced trophoblast stem cells (iTSCs) [58, 59]. We also sequenced non-PE control (*N* = 24), gestational hypertension (GHT) (*N* = 5), and PE (*N* = 14) placentas by genome-wide CpG capture bisulfite sequencing (DNAm-seq) and ATAC (Fig. 1a). These data were subjected to a multimodal integrative analysis. In the single-cell analysis, all non-PE samples were defined as control.

For scRNA analysis, datasets were integrated with potential batch effects removed by Harmony [60] prior to cell cluster identification using Seurat [61]. By identifying differentially expressed genes in each single-cell cluster (Fig. 1f), we annotated the major cell populations in the maternal–fetal interface in the scRNA dataset (“Methods,” Fig. 1b and Additional file 1: Fig. S1d-e). These included the major cell populations in trophoblast, i.e., progenitor villous cytotrophoblast (VCTp) cells, villous trophoblast (VCT) cells, syncytiotrophoblast (SCT) cells, and extravillous trophoblast (EVT) cells; stromal cells with mixed arterial and venous endothelial cells, including vascular smooth muscle cells, pericytes, and fibroblasts; and immune cells, including macrophages, monocytes, NK cells, T cells, and B cells. These cells could be classified into three distinct groups based on their origin: fetal-originated cells (trophoblasts and fetal macrophage Hofbauer cells), maternal-originated cells (including T lymphocytes, B lymphocytes, maternal macrophages, monocytes, and NK cells), and mixed-origin cells (stromal cells, including endothelial cells, fibroblasts, smooth muscle cells, and pericytes) [55]. We further analyzed the expression levels of fetal- and maternal-cell-specific gene sets reported in Vento-Tormo et al. [55] in our scRNA dataset (Fig. 1b) to determine the cell origin of the mixed-origin cells (Fig. 1c and Additional file 1: Fig. S2a–l). All cell lineages described in Fig. 1b are present in the majority of the samples in this study (Additional file 1: Fig. S1e and Additional file 3: Table S2). Cells from control and PE placentas were well intermingled in each population (Fig. 1d and Additional file 3: Table S2). As expected, the iTSC population cultured in vitro [58, 59] was primarily colocalized with trophoblast progenitors (VCTp, Fig. 1d). Compared with control placentas, PE placentas were depleted in terminally differentiated SCT and EVT, were relatively enriched in VCT, and exhibited an increased count of stromal cells (Additional file 1: Fig. S1d, e). We integrated scATAC data with scRNA data (Methods) to use scRNA labels as a guide for annotating these cells (Fig. 1e). Cell clustering and annotation were validated by analyzing chromatin accessibility around known marker genes (Fig. 1g). In general, scATAC-classified cell types showed consistent chromatin opening on known marker gene promoters (Additional file 1: Fig. S3a, b).

To identify cell types that are associated with PE pathogenesis, we first compared cell type composition between PE and control donors. PE placentas exhibited a disproportionate composition of cell types, specifically trophoblasts, endothelial cells, and immune cells (Additional file 1: Fig. S1d). Using Scissor [62], we performed RNA expression similarity correlation analysis against public RNA-seq dataset of PE and control placentas [63, 64] (Fig. 1h inset, Additional file 1: Fig. S4 and S5) to search for scRNA cells whose expression profiles were associated with a specific phenotype. Orthogonally, we evaluated the association between single cells and the hereditary risk of PE by performing an enrichment analysis of PE-associated GWAS loci across scATAC profiles using SCAVENGE [65]. In the scRNA-based Scissor analysis, the PE phenotype was associated with endothelial cells, trophoblasts, and monocytes, whereas most other immune cells were associated with normal pregnancy (Fig. 1h, inset). In contrast, in the scATAC-based SCAVENGE analysis, hereditary risk loci for PE were associated with endothelial cells, trophoblasts, and macrophages (Fig. 1h, inset). Together, the two results identified EVT and endothelial cells were most significantly associated with the PE phenotype in terms of both hereditary risk and phenotypic association (Fig. 1h), indicating that they were likely the key cell types underlying PE pathogenesis.

PE-associated trophoblast, including VCTp, VCT, and SCT, were predominantly enriched in PE placentas (Additional file 1: Fig. S4a, b). The relatively higher enrichment of hereditary PE risk loci in VCT/VCTp than phenotypic enrichment suggests that PE risk loci might affect the differentiation of these cells rather than their function (Fig. 1h).

### Developmental delay of PE trophoblast

**Fig. 2:**
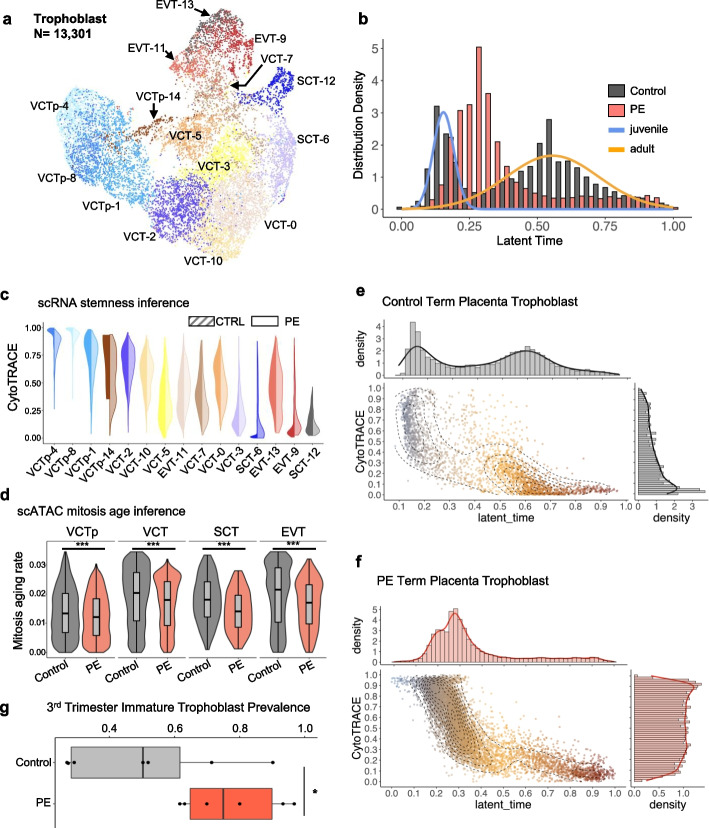
Developmental delay in PE trophoblasts. **a** UMAP projection of trophoblasts in scRNA. **b** RNA velocity derived latent time distribution for PE (pink) or control (gray) trophoblasts. Control trophoblasts were segregated into a juvenile (blue) population and an adult population (orange), while PE trophoblasts were stuck in the middle. **c** Stemness prediction by CytoTRACE of scRNA clusters of control (solid) and PE (transparent) trophoblasts. Significant delayed maturation was found for PE EVT and SCT. **d** Mitosis aging rate (age increase per gestational week) inferred by *EpiTrace* of scATAC clusters of control (gray) and PE (pink) trophoblasts, indicating significantly reduced cell proliferation/division in PE trophoblasts. *P*-values were tested by Wilcox test, raw *P*-value. **e** Scatter plot of stemness prediction (*y*-axis) and latent time of control trophoblasts, showing that the juvenile population is immature, and the developed population is mature. Distribution of latent time and CytoTRACE score were shown at the *x*- and *y*-axis, respectively. **f** In PE, developmentally delayed trophoblasts were found to be immature. **g** The frequency of immature trophoblast in termed placentas is significantly increased in PE compared to control (*P* = 0.03, *P*-value was tested by *t*-test, raw *P*-value). Immature trophoblasts were defined as CytoTRACE score < 0.2 and Latent Time value < 0.4, based on a Gaussian mixture model segregation

To investigate the role of trophoblasts in PE, we used RNA velocity to time the developmental age of trophoblasts in control and PE. A total of 13,301 trophoblast cells were identified and classified into four distinct types: VCTp, characterized by high proliferation and division activity (Additional file 1: Fig. S6a, b), VCT, SCT, and EVT, each defined by canonical markers (Fig. 2a and Additional file 1: S7a-b). The “developmental age” of trophoblasts was determined by RNA velocity in terms of latent time [66] and clustered using a bimodal Gaussian mixture model [67] (Fig. 2b). Control trophoblast cells (indicated as gray in Fig. 2b) were classified into two populations: a “juvenile” population, exhibiting a mean latent time of 0.15, and an “adult” population, exhibiting a mean latent time of 0.56 (Fig. 2b). However, PE trophoblast cells predominantly exhibited an intermediate phenotype, i.e., a phenotype between the juvenile and adult stages (Fig. 2b). Using CytoTRACE [68], we assessed trophoblast differentiation and identified significant maturation delay in PE undifferentiated progenitors (VCTp-4) and terminally differentiated trophoblasts (VCT-3, SCT-6/12, and EVT-9/13) (Fig. 2c and Additional file 4: Table S3). We performed an orthogonal analysis using *EpiTrace* [69] to infer trophoblast “mitosis aging rate” (age increase per gestational week) from scATAC data, showing that PE trophoblast cells exhibited a significantly decreased proliferation rate in each lineage (Fig. 2d).

By correlating the latent time with CytoTRACE scores, we determined that the juvenile cells correspond to the immaturely differentiated while adult cells are maturely differentiated (Fig. 2e, f). Majority of trophoblasts were terminally differentiated in control placentas; however, there was a significant increase of immature trophoblasts and decrease of mature ones (Fig. 2e, f and Additional file 1: Fig. S8a). Cell cycle scoring based on scRNA data showed that actively dividing cells (G2M/S phase) were slightly increased in terminally differentiated trophoblasts, i.e., VCT-3/SCT-12/EVT-9 (Additional file 1: Fig. S8b), in PE.

In PE, excessive immature trophoblasts exist in the placenta (Fig. 2g). This phenomenon is not solely explained by earlier delivery of PE pregnancies, because the “adult” population of trophoblast emerge as early as 6–12 gestational week (gw) in control pregnancies (Additional file 1: Fig. S9a and S10). Trophoblasts in control pregnancies could be clearly separated into juvenile or adult population and the majority of juvenile populations in 3rd control placentas enrich in fetal sides (Additional file 1: Fig. S9b). Trophoblast on maternal side of control placentas show greater maturity than those on fetal sides (Additional file 1: Fig. S9b). However, in all PE pregnancies, trophoblasts were developmentally stalled at an intermediate stage (Additional file 1: Fig. S9c and Additional file 1: Fig. S10). In the comparison groups, 29gw control vs 32gw PE/30gw control vs 35gw PE/ 38 gw control vs 36 gw PE, though control placentas are at earlier gestational week than PE, they exhibit a higher proportion of adult populations (Additional file 1: Fig. S9b-c and Additional file 1: Fig. S10).

On the basis of the mitosis rate, mitosis activity, and developmental maturity data, we concluded that trophoblast development is slowed in PE, especially in terminally differentiated SCT and EVT. Validation with an external dataset GSE173193 [70] showed similar trophoblast developmental delay in PE by CytoTRACE analysis (Additional file 1: Fig. S11).

We then delineated the developmental trajectory of trophoblast cells by RNA velocity [66] and subsequently determined three major developmental trajectories culminating in VCT-3, SCT-12, and EVT-13/9, respectively (Additional file 1: Fig. S12a-b). In PE trophoblast, the trajectory separated from control since early stage (Additional file 1: Fig. S12a-b), leading to a significant reduction in terminally differentiated cells (EVT-13/SCT-12) in PE (Additional file 1: Fig. S8a). These observations were consistent with orthogonal analysis with trajectories generated using Slingshot [71] (“Methods”) on diffusion map projection (Additional file 1: Fig. S12c-d). Together, these results indicate a developmental trajectory switch in PE trophoblasts, characterized by early-onset developmental delay, resulting in impaired maturation of terminally differentiated EVT and SCT.

### Early transcriptional defect in trophoblast development in PE

**Fig. 3:**
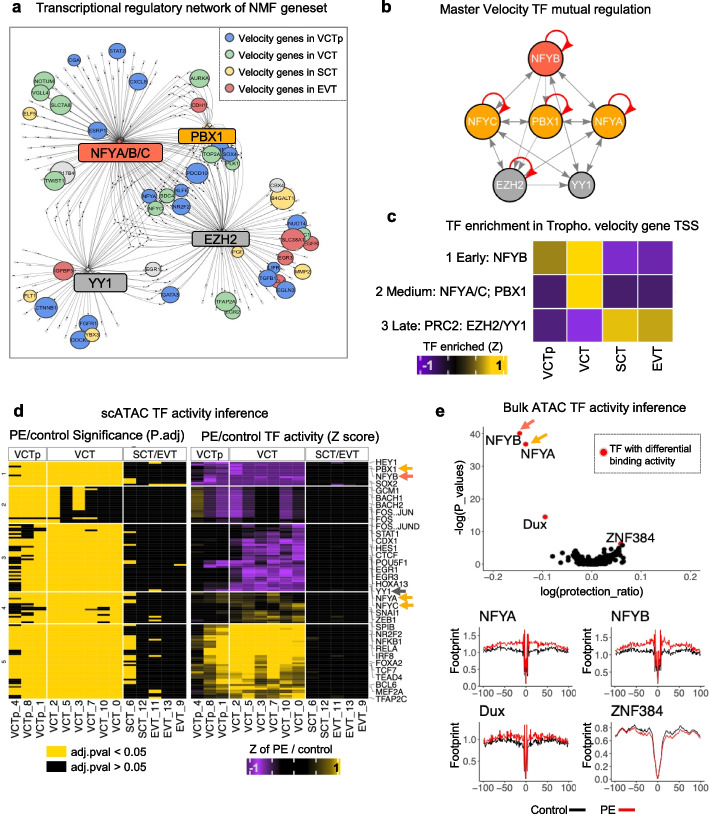
Early transcriptional defect during trophoblast development in PE. **a** The promoters of developmental switch genes (“velocity gene”) during trophoblast development predicted by RNA velocity are enriched with six core transcription factors in this gene set: NFYA, NFYB, NFYC, PBX1, EZH2, and YY1. **b** Transcriptional regulation network between the core transcription factors shows three tiers of regulation with NFYB controlling middle layer NFYA/C and PBX1, which in turns controls EZH2 and YY1. **c** TF binding activity, inferred by scATAC, of core transcription factors on the promoter of velocity genes, showing that NFYB is most active in VCT progenitor, whereas NFYA/C and PBX1 are most active in VCT, and EZH2/YY1 are most active in terminally developed EVT/SCT. **d** Heatmap of differential TF binding activity between PE and control in each trophoblast clusters. Left: Statistical significance (yellow: significant; black: non-significant), *P*-values were tested by Wilcox test and adjusted with Holm method; Right: *Z*-score of TF binding activity. Differential TF binding is most evident in early-stage VCTp and intermediate-stage VCT but not the terminally developed trophoblasts (SCT/EVT), suggesting an early developmental defect primes the pathogenic phenotype in terminally differentiated trophoblasts in PE. **e** Differential TF binding activity (protection ratio) between PE and control inferred by bulk ATAC-seq, showing significantly downregulated ZGA-associated transcription factors NFYA/B and DUX activity, and elevated ZNF384 activity in PE placenta. Bottom: averaged genome-wide chromatin accessibility profile around NFYA, NFYB, DUX and ZNF384 TFBS

To determine the molecular mechanism underlying trophoblast developmental delay in PE, we analyzed transcriptional network among “velocity genes” identified by RNA velocity. Six master transcriptional binding site motifs regulating trophoblast developmental velocity genes were identified, including ZGA-related *NFYA*/*NFYB*/*NFYC* [46, 72], the polycomb repressor complex 2 (PRC2) complex members *EZH2* [73] and *YY1* [74], and a homeobox TF, *PBX1* (Fig. 3a). By examining TF binding sites in each TF’s promoter, we found that NFYB served as the foremost upstream regulator, binding to the promoters of all the other five master TFs. NFYA/NFYC/PBX1 functioned as intermediate layer regulators, regulating both themselves and EZH2/YY1, which were determined to be downstream, bottom layer regulators (Fig. 3b).

The enrichment analysis of TF binding sites in trophoblast cell type-specific velocity genes reveals that majority of VCTp-expressed velocity genes were controlled by NFYB (Fig. 3c), whereas those in VCT were regulated by NFYA/C and PBX1 and velocity genes in terminally differentiated SCT/EVT were exclusively controlled by PRC2 (Fig. 3c). Thus, trophoblast development was modulated by a cascade of master TFs. Despite no changes in RNA expression of master TFs (Additional file 1: Fig. S13a), we observed altered TF activities in PE with scATAC data: decreased NFYB/PBX1 activities in VCTp and VCT clusters; decreased NFYA/NFYC/YY1 activities in VCT clusters (Fig. 3d and Additional file 1: S13b). Due to a technical reason, we were unable to directly infer EZH2 activity in the scATAC dataset together with other TFs. However, the activities of two of its upstream master regulators E2F4 and E2F7 were downregulated in PE trophoblasts (Additional file 1: Fig. S14). Changes in activities of TFs related to trophoblast differentiation, including GCM1, TEAD4, and TFAP2C, were also detected (Fig. 3d and Additional file 1: Fig. S13b).

We further validated the relative expression level of gene sets under control of the master TFs with trophoblast in GSE173193 dataset [70]. We found that for all but one (NFYA gene set in SCT) cases, master TFs controlled genes were expressed in lower levels in PE cells compared to control cells (Additional file 1: Fig. S15), confirming that the master TFs-regulated gene network is disrupted in PE trophoblasts.

A complementary assay was conducted to profile the transcriptional regulation through bulk ATAC sequencing of additional frozen placenta samples for validation (“Methods,” Additional file 1: Fig. S16 and Additional file 5: Table S4).

From bulk ATAC data, we inferred TF binding activity (protection score) by comparing control and PE placentas. In line with scATAC findings, ZGA-active TF, including DUX [44], NFYA/NFYB [46, 72], showed significantly decreased binding activity in PE (Fig. 3e). Collectively, these results collaboratively revealed a disrupted core transcription network in PE, participating trophoblast lineage determination is disrupted in PE.

### Defective de novo DNA methylation on PRC2-controlled regulatory loci results in imbalanced imprinted gene expression in placenta

Transcription network dysregulation during early trophoblast development in PE suggested that either the expression or the function of the dysregulated master TFs were defective in PE. The expression of master TFs, however, did not differ between PE and control trophoblasts (Additional file 1: Fig. S13a). Notably, both the NFY family and PRC2 are closely associated with DNA methylation; the NFY family of TFs binds to methylated retrotransposon sequences [45] and the PRC2 complex binds to methylated CpG islands [73, 75]. Therefore, we hypothesized that, in PE, the dysregulation in the activity of master TFs in trophoblast may be due to defective DNA methylation. We then analyzed the epigenomic profiles of PE and control placentas to determine the mechanism underlying early transcriptional dysregulation.

The placenta develops from extraembryonic tissue derived from the trophectoderm in the blastocyst while undergoing extensive epigenetic reprogramming, including de novo DNA methylation. By profiling whole-genome DNA methylation patterns in PE and control placentas, we identified a total of 60,515 differentially methylated loci in PE, including 2710 hypomethylated (PE-hypo) continuously differentially methylated regions (DMRs) and 1271 hypermethylated (PE-hyper) continuously DMRs (Additional file 6: Table S5 and Additional file 1: Fig. S17a-b). PE-hypo DMRs were significantly hypomethylated in the maternal side, but not the fetal side, of PE placentas (Additional file 1: Fig. S18a). In contrast, hypermethylation of PE-hyper DMR was more significant in the fetal side of PE placenta (Additional file 1: Fig. S18b). These results suggest that DNA hypomethylation sites are specific for PE differentiated trophoblasts, whereas DNA hypermethylation sites are associated with all PE trophoblasts.

**Fig. 4:**
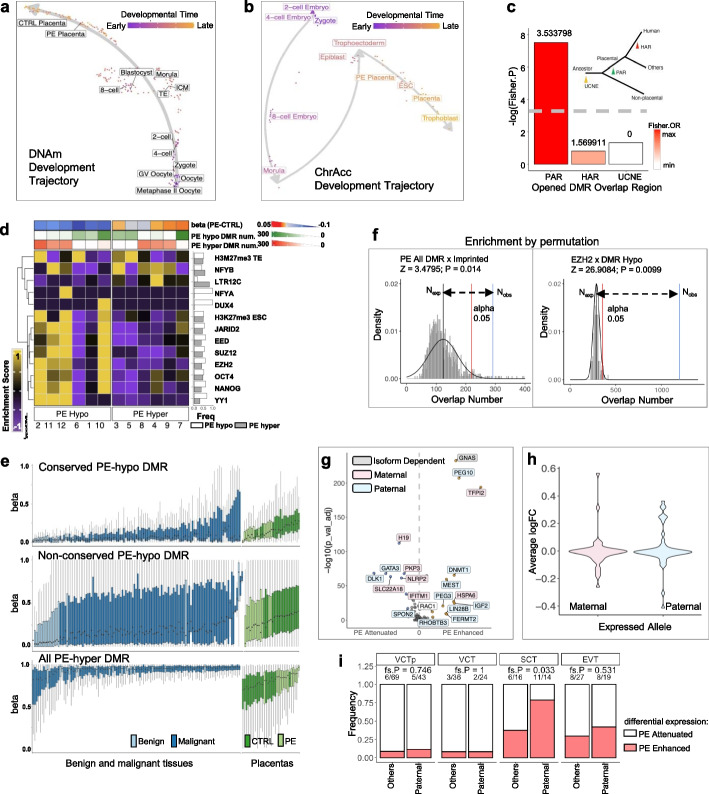
De novo DNA methylation defect on PRC2-controlled placenta regulatory loci results in imbalanced imprinted gene expression. **a** Cell evolution trajectory from zygote to placenta inferred by genome-wide DNA methylation sequencing data, showing delayed development of PE placenta in terms of DNA methylation. **b** Cell evolution trajectory from zygote to placenta inferred by chromatin accessibility on PE-specific differentially methylated region (DMR), showing delayed development of PE placenta in terms of chromatin accessibility on PE DMR. **c** Enrichment of opened (accessible) PE DMR in different types of genomic elements: ultraconserved noncoding elements (UCNE), placenta-accelerated genomic region (PAR), and human-specific accelerated region (HAR). Opened PE DMR is enriched in PAR but not HAR or UCNE, suggesting that they are functionally relevant to placental development, as the PAR regions undergone Darwinian positive selection in placental animals. *P*-value and odd’s ratio (OR) were calculated by Fisher’s exact test. Inset: schematic diagram of animal evolution and the time point where genomic regions (UCNE/PAR/HAR) were subjected to evolutionary selection. **d** Enrichment heatmap of TFBS and chromVAR-annotated genomic regions in PE DMR, showing that PRC2 complex-related genomic regions (H3K27me3 in trophectoderm and ESC, and the PRC2 complex components SUZ12, EZH2, JARID2, EED) were more likely to be subjected to DNA hypomethylation instead of hypermethylation in PE. On the other hand, NFYB binding sites and LTR12C elements are more likely to be hypermethylated in PE. **e** Sample-wise CpG methylation levels (box-and-whisker plot with mean, 0%-25%-75%-100% quantiles) of conserved PE-hypomethylated loci (top), human-specific PE-hypomethylated loci (middle), and PE-hypermethylated loci (bottom) of normal tissue (light blue, left), cancer tissue (dark blue, left), and placenta of normal pregnancy (dark green, right) and preeclampsia (light green, right) showing that PE-hypomethylated loci undergone similar hypermethylation in both placenta and cancer, suggesting these regions are the ones subjected to de novo DNA methylation during extraembryonic tissue development. In contrast, DNAm levels on PE-hypermethylated regions do not segregate cancer from non-malignant tissues, suggesting that they are under control of a different molecular mechanism. **f** Significant enrichment of all PE DMR on imprinted genes and PE-hypo DMR on h1 hESC EZH2 binding sites (*P* = 0.014, *Z* = 3.4795, PE DMR vs imprinted genes; *P* = 0.0099, *Z* = 26.9084, PE-hypo DMR x EZH2; *Z* test. Basal distribution was done by permutation performed × 1000 times. Expected number of overlap: black vertical line; alpha = 0.05 number of overlap: red vertical line; observed number of overlap: blue vertical line) on imprinted genes, indicating that imprinted genes were affected by DNA methylation defects in PE. **g** Differential expression of imprinted genes between PE and control single trophoblasts. Blue: maternal allele expressed (paternally imprinted); Pink: paternal allele expressed (maternally imprinted). *P*-values were calculated by Wilcox test and adjusted based on Bonferroni correction. **h** Log fold-change of imprinted gene expression between PE and control trophoblasts, showing that expressed maternal alleles (pink) is likely to be downregulated, while expressed paternal alleles (blue) is likely to be upregulated in PE. **i** Frequency of imprinted genes that has shown PE-specific upregulation (red: PE-enhanced) or downregulation (white: PE-attenuated) in trophoblasts, showing dysregulated imprinted gene expression in differentiated trophoblasts, while the paternal alleles were significantly more likely to be upregulated in SCT (*P* = 0.033, Fisher’s exact test)

By averaging the DNA methylation level per DMR, we projected DNAm-seq data pertaining to sequenced control and PE placentas together with a public single-cell WGBS data set pertaining to early embryonic development [42] as a UMAP. PE placentas stalled along the trajectory of trophoectoderm development toward trophoblast cells, indicating that DNA methylation in PE placentas was delayed (Fig. 4a). Trophoectoderm developmental trajectory based on chromatin accessibility at all DMRs in placental tissues and single cells of early embryonic developmental stages from publicly available data set [45, 76] suggested a similar scenario, i.e., PE placentas were situated midway along the trajectory from trophectoderm toward trophoblast (Fig. 4b). Together, these results indicate that the epigenome reprogramming during extraembryonic development is delayed in the PE placenta.

We defined open DMRs as regions with trophoblast ATAC peak and differential DNA methylation. Open DMRs were significantly enriched only in placenta-accelerated genomic regions [77] but not human-specific accelerated regions [78] and vertebrate-specific ultraconserved noncoding elements [79] (Fig. 4c), suggesting the functional relevance of DMRs in placenta development. We then annotated PE-specific DMRs using chromVAR [80]. Compared with PE-hyper DMRs, PE-hypo DMRs were enriched in PRC2 complex-related TF binding sites, including EED/EZH2/JARID2/SUZ12/YY1 (Fig. 4d), implying that defective DNA methylation in PE placenta affects PRC2 complex binding and function. Furthermore, PE-hypo DMRs covered loci that underwent de novo DNA methylation during epigenetic reprogramming in extraembryonic tissues [50], as these loci were de novo methylated during extraembryonic tissue development (Additional file 1: Fig. S17b) and were extensively methylated in cancer tissue (Fig. 4e). DNA methylation on PE-hypo DMRs was severely aberrant in PE placentas (Fig. 4e), indicating a specific defect in extraembryonic tissue-specific de novo methylation. Conversely, PE-hyper DMRs (PE-Hyper cluster 4) were enriched in retrotransposon sequences (Fig. 4d). Typical hypomethylation in maternally imprinted retrotransposons, particularly those of the long terminal repeat 12 (LTR12) family of endogenous retroviruses (ERVs), was disrupted in PE placentas (Fig. 4d and Additional file 1: Fig. S19a). These maternally imprinted and paternally hypomethylated LTR12C elements were transiently active during ZGA (Additional file 1: Fig. S19b), possibly owing to the binding of NFYA/B/C in their terminal repeat sequences [45]. In fact, hypomethylated LTR12C in human sperm are more likely to be hypermethylated in PE (Additional file 1: Fig. S20), suggesting that the establishment or post-zygotic maintenance/refinement of DNA methylation imprinting may be disrupted in PE.

Imprinting is modulated by DNA methylation and H3K27me3 modifications [81–83]. In PE, DMRs were significantly enriched in imprinted genes and binding sites of PRC2-related genes, such as *EZH2* (Fig. 4f and Additional file 1: Fig. S21), suggesting that de novo DNA methylation may further regulate imprinted gene expression in addition to the DNA methylation profile established during germline development. If such a case, we expect dysregulated imprinted gene expression in the PE placenta. This was validated by analyzing RNA expression in the scRNA dataset, which showed significant differential expression of imprinted genes in PE trophoblast (Fig. 4g, h and Additional file 7: Table S6). Notably, maternally imprinted genes were more likely to be upregulated in PE, whereas paternally imprinted (maternal allele expressed) genes were more likely to be downregulated in PE (Fig. 4g, h and Additional file 7: Table S6). The overexpression of imprinted genes was mostly exhibited by terminally differentiated SCT and EVT (Fig. 4i). In SCT, significantly more paternal allele-specific genes were overexpressed in PE (Fig. 4i, Additional file 1: Fig. S22 and Additional file 7: Table S6).

Imprinting could also be regulated by H3K27me3 modification. We additionally profiled H3K27me3 in PE and control placenta using CUT&Tag assay. In PE placentas, we identified 32,409 genomic regions with increased H3K27me3 modification (PE-gain), and 13,284 genomic regions with decreased H3K27me3 modification (PE-lost), compared to control placentas (Additional file 1: Fig. S23a-c). The PE-lost regions are highly enriched around EZH2 binding site (Additional file 1: Fig. S23d). Overall, EZH2 loci are associated with decreased H3K27me3 modification in PE placenta (Fig. S23c). Many of these PE-lost, EZH2-bound regions were located on imprinted genes (Additional file 1: Fig. S24a). Compared to the maternally imprinted loci (expressing the paternal allele), paternally imprinted loci (expressing the maternal allele) are more likely to lost H3K27me3 modification in PE placentas (Additional file 1: Fig. S24b). Together, our results indicate that both molecular mechanisms regulating imprinted gene expression, namely H3K27-trimethylation and extraembryonic tissue-specific de novo DNA methylation, are defective in PE.

### Excessive maternally imprinted gene product PEG10 in PE

**Fig. 5:**
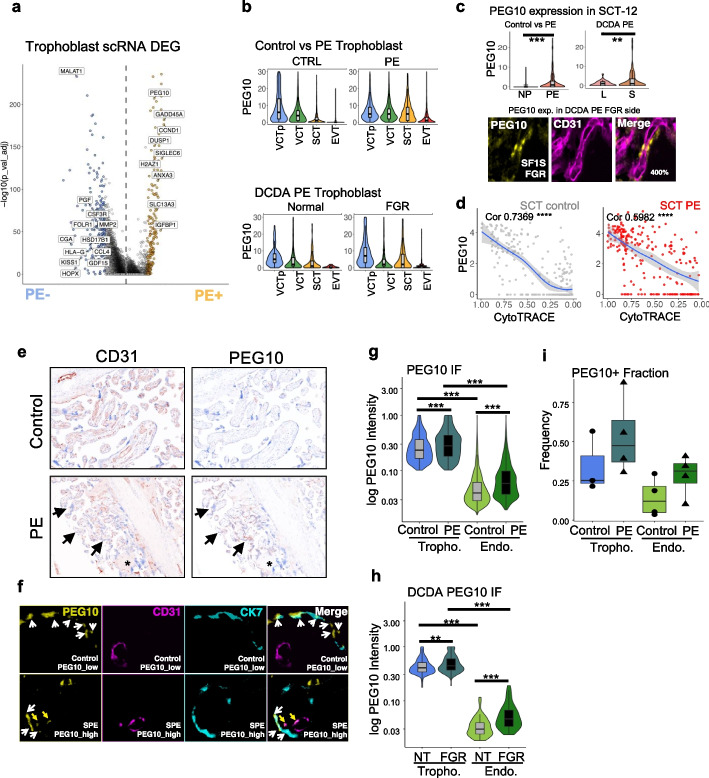
Excessive maternal imprinted gene product PEG10 in PE. **a** Differential expression of genes between PE and control trophoblasts, showing PEG10 among the most significantly upregulated genes in PE trophoblasts. *P*-values were calculated by Wilcox test and adjusted based on Bonferroni correction. **b** Expression of PEG10 in control, PE, and normal/FGR placentas from DCDA sIUGR twins, showing that PEG10 expression is gradually downregulated as trophoblast differentiate but this trend is blocked in PE or FGR placenta. **c** Significantly upregulated PEG10 expression in terminal SCT (SCT_12) cluster in PE or sIUGR FGR placenta (S) compared to their controls (L). *P*-value was determined by *t*-test, **: *P* < 0.01, ***: *P* < 0.001. Staining of PEG10 (yellow) is found in SCT lining the blood vessel (CD31 + , purple) but we noticed a faint staining of PEG10 in CD31 + endothelial compartment in this FGR placenta sample. **d** Single-cell PEG10 expression (*y*-axis) and cell maturation (1-CytoTRACE, *x* axis) in SCT of PE and control placenta. PEG10 expression is significantly correlated with cell maturity in an inverse manner (Cor.coef: 0.7369 (control)/0.5982 (PE); *P*-value: both < 2.2*1e − 16), indicating that PEG10 overexpression in PE is due to developmental delay of trophoblasts. **e** OPAL multiplex immunohistochemistry staining (brown: IHC stain; blue: DAPI) of CD31 (left) and PEG10 (right) in control (top) and PE (bottom) placentas, showing villous void of (asterisk) or with narrow, deformed fetal capillary vessel (arrows) in PE, together with excessive PEG10 expression. **f** OPAL multiplex immunohistochemistry staining of PEG10 (yellow), CD31 (purple), CK7 (cyan) in control (top) and PE (bottom) placenta, showing that PEG10 is found not only in non-CD31 trophoblast (CK7 +) cells in control but is additionally found in endothelial (CD31 +) cells in PE. **g** Immunofluorescence intensity of PEG10 in trophoblasts (T) or endothelial cells in normal (control) or PE placenta, showing significantly elevated PEG10 protein level in PE trophoblasts as well as endothelial cells. *P*-values were tested by Wilcox test, not adjusted. **: *P* < 0.01, ***: *P* < 0.001. **h** Immunofluorescence intensity of PEG10 in trophoblasts or endothelial cells from the paired normal (NT) and FGR placentas from the sIUGR pregnancy, showing significantly elevated PEG10 protein level in FGR cells. *P*-values were tested by Wilcox test, not adjusted. **i** Frequency of trophoblasts or endothelial cells that are positive with PEG10, in control and PE placentas, showing increased PEG10-positive cells in abnormal placenta. *Abbreviation*: DCDA: Dichorionic diamniotic twin pregnancy; sIUGR: selective intrauterine growth retardation. NT: normal fetus; FGR: fetal growth restriction; SPE: severe preeclampsia

We performed differential gene expression analysis in the trophoblast scRNA dataset. PE trophoblasts overexpressed *PEG10*, *GADD45A*, *CCND1*, *SIGLEC6*, and *H2AZ1* (Fig. 5a and Additional file 7: Table S6). On the other hand, the lncRNA *MALAT1* and the canonical mature trophoblast marker genes *CGA*, *HLA-G*, and *PGF* are downregulated in PE trophoblasts (Fig. 5a and Additional file 7: Table S6). GSEA enrichment of differentially expressed genes showed that PE trophoblasts exhibited increased gene set activities in “G2M checkpoint,” “E2F targets,” and “MYC targets” pathways (Additional file 1: Fig. S25a). These results are in concordance to the function of PE upregulated genes such as *CCND1* (an important factor that regulates cell cycle) and *GADD45A* (an important factor that regulates chromatin accessibility and cell cycle). The results suggest a scenario that in PE trophoblast, *GADD45A* expression is induced to arrest the cells at G2/M checkpoint from further division and differentiation, which is supported by cell cycle gene set expression scoring on single-cell RNA-seq dataset (Additional file 1: Fig. S25b).

Notably, we found an imprinted gene *PEG10* was significantly upregulated in all trophoblast cells across various trophoblast lineages in PE (Fig. 5a and Additional file 1: Fig. S26). *PEG10* was the most highly upregulated maternally imprinted gene in PE (Fig. 4g, Additional file 1: Fig. S22 and Additional file 7: Table S6). *PEG10*, which is a retrotransposon gene involved in trophoblast stem cell specification [84], functionally implicated in placenta development and embryogenesis [85].

In control samples, *PEG10* expression gradually decreased during trophoblast differentiation. In detail, PEG10 expression was high in VCTps and VCTs, but low in SCTs and EVTs (Fig. 5b, top panel). In contrast, in PE, the decrease in *PEG10* expression was delayed, with significantly elevated expression in SCTs and EVTs both in our in-house sequenced trophoblast and external dataset GSE173193 [70] (Fig. 5b, top panel, Additional file 1: Fig. S11g and h). In the paired sIUGR placentas, the trend of *PEG10* expression in the trophoblasts in normal fetus placenta was similar to control, whereas the FGR fetus placenta trophoblast phenocopied PE placenta (Fig. 5b, bottom panel). Since sIUGR placentas share a similar maternal environment, these results indicate that cell-autonomous transcriptional regulation underlies *PEG10* expression dynamics.

*PEG10* overexpression was most evident in terminally differentiated SCT-12 in either PE or sIUGR/FGR placenta (Fig. 5c). Immunohistochemistry validated the protein expression of PEG10 in perivascular SCTs, and weak but consistent PEG10 staining was observed within the CD31 + endothelial cells (Fig. 5c). Correlation analysis between the differentiation status deduced by CytoTRACE and *PEG10* expression levels in SCTs suggested that *PEG10* expression was a function of differentiation status in both control and PE placenta (Fig. 5d). Hence, the delayed maturation of trophoblasts, most evidently in SCTs, underlies *PEG10* overexpression.

Through OPAL multiplex immunohistochemistry, we determined the protein expression of *PEG10* in singleton PE, control, and DCDA PE placentas (Fig. 5e, f). The PE placenta exhibited an increased degree of PEG10 staining in villous that was void or contained deformed and narrow fetal capillaries (Fig. 5e). Consistent with the results of scRNA, PEG10 immunofluorescent intensity in trophoblasts (CK7 +), and the fraction of PEG10 + trophoblast were significantly increased in PE (Fig. 5e–i) and sIUGR placentas (Fig. 5h). In addition to PE-specific PEG10 overexpression in trophoblast, we observed a significant increase in PEG10 expression in CD31 + endothelial cells, in terms of both staining intensity (Fig. 5f–h) and cell count (Fig. 5i). These results indicate that trophoblasts exhibiting developmental delay in FGR (sIUGR) and PE placenta overexpress *PEG10*.

### PEG10 is transferred from the trophoblast to maternal endothelial cells

**Fig. 6:**
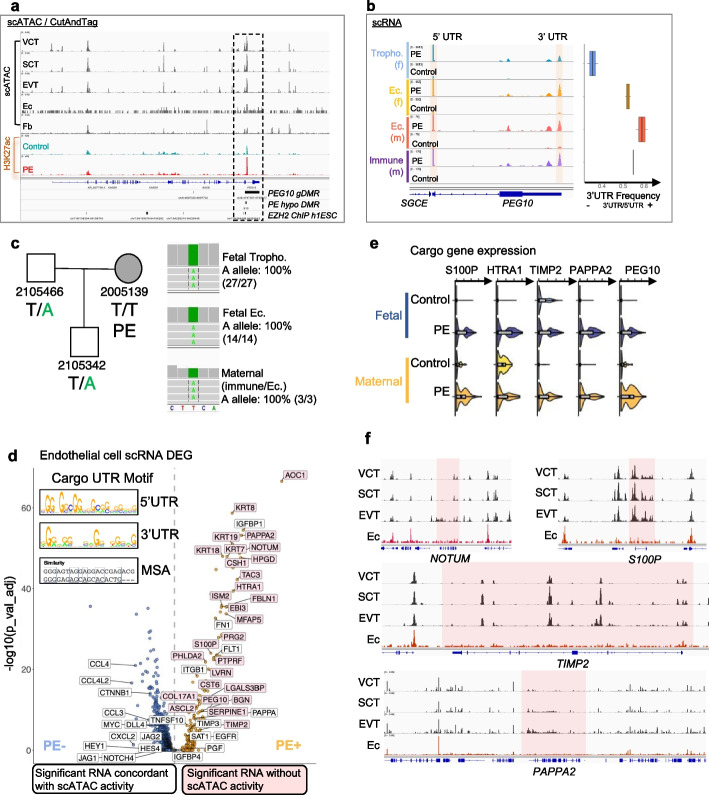
PEG10 is transferred from trophoblast to maternal endothelial cells. **a** Chromatin accessibility of VCT, SCT, EVT, endothelial cells (Ec) and fibroblasts (Fb), and H3K27ac Cut-and-Tag signals (Additional file 1: Fig. S27) from control and PE placentas, around the PEG10/SGCE locus. Compared to trophoblasts, Ec and Fb have little chromatin accessibility on PEG10 locus, despite the significant upregulation of PEG10 transcripts and proteins in PE Ec. The PEG10 locus is subjected to imprinting (gDMR), controlled by EZH2, hypomethylated in PE, and shows PE-specific increase of H3K27ac modification (Additional file 1: Fig. S27), indicating PE-specific epigenomic activation of PEG10 locus. **b** scRNA sequencing reads from fetal trophoblasts, fetal endothelial cells, maternal endothelial cells, and maternal immune lymphoid (B/NK/T) cells on PEG10 genomic region. PE cells show significantly higher PEG10 expression. RNA reads covering PEG10 3′UTR in trophoblasts are significantly lower compared to those in non-trophoblast cells, suggesting most PEG10 transcript in non-trophoblast cell are mature and spliced. **c** A trio pedigree of a PE pregnancy, where the mother (2005139) suffered SPE during pregnancy. The father and progeny carried a paternal-specific PEG10 SNP allele (T/A) and the mother is wild-type (T/T). scRNA sequencing reads from the 2005139 placenta showing that all transcripts covering the SNP, regardless of their cell-of-origin, carried the paternal A allele, suggesting a fetal origin of these RNA in maternal cells. **d** Differential expressed genes between PE and control endothelial cells, labelled with scATAC-silent, scRNA-upregulated genes (pink). Transcripts of these genes shared common 5′UTR and 3′UTR sequence motives with sequence similarity (inset), suggesting that they are likely to be bound with similar RNA-binding protein and might be transported from trophoblast to endothelial cells with this protein. *P*-values were calculated by Wilcox test and adjusted based on Bonferroni correction. **e** scATAC-silent, scRNA-upregulated genes in fetal and maternal endothelial cells from control and PE placenta, showing that these transcripts were similarly upregulated in fetal and maternal endothelial cells in PE. **f** Aggregated scATAC coverage tracks for trophoblasts (VCT: *N* = 10299 + 47086; SCT: *N* = 230 + 3495; EVT: *N* = 1062 + 6366, control + PE) and endothelial cells (Ec: *N* = 293 + 395, control + PE) on cargo genes *NOTUM*, *S100P*, *TIMP2*, and *PAPPA2*. Cargo gene region are highlighted in pink. Adjacent genomic regions were shown to control for scATAC validity in Ec

*PEG10* is an ancient retroviral Gag gene encoded by a domesticated endoretrovirus (ERV). It binds to 5′ and 3′ UTR of its transcript, leading to the formation of virus-like particles (VLPs) [86]. These VLPs are secreted via the exosome pathway and transferred into other cells. PEG10 protein and RNA transcripts levels were increased in PE trophoblasts and non-trophoblast cells, including endothelial cells (Fig. 5a, e–h and 6b, e). However, scATAC data showed that the *PEG10* locus was only accessible in trophoblast cells and inaccessible in all other cells (Fig. 6a and Additional file 1: Fig. S27a). The *PEG10* locus was differentially methylated in the gamete germline (termed germline differentially methylated region, gDMR [87], Additional file 1: Fig. S27a). We found that the *PEG10* gDMR was bound by the PRC2 complex component EZH2 and that it exhibited specific DNA hypomethylation and increased H3K27ac modification in the PE placenta (Fig. 6a and Additional file 1: Fig. S27b), indicating either a loss-of-imprinting or loss-of-de-novo*-*methylation around the region, resulting in reduced PRC2 binding and dysregulated expression of *PEG10* during trophoblast lineage differentiation in PE.

We further analyzed the structure of *PEG10* RNA transcript across various cell types. Compared with those in control, single cells in PE exhibited increased expression of *PEG10* RNA transcripts (Fig. 6b). In fetal trophoblast, the sequenced RNA reads were biased toward the 5′ UTR, suggesting active ongoing transcription (Fig. 6b). On the contrary, in other cells, including fetal and maternal endothelial cells and maternal immune cells, the sequenced RNA reads were biased toward the 3′ UTR. The fraction of reads corresponding to the 3' UTR was increased in non-trophoblasts compared to trophoblasts (Fig. 6b, right panel), suggesting more mature transcripts in non-trophoblasts. These results, in concordance with reported 3′ UTR-biased RNA levels in exosome [88] and the transfer of PEG10 VLPs between cells [86], suggest that *PEG10* gene product may be exported to non-trophoblast cells from trophoblasts.

In our cohort, we identified a family where the father and fetus were heterozygous for a *PEG10* SNP allele chr7:94665871 T > A (genotype T/A), whereas the PE mother was homozygous for the wild-type allele on this locus (genotype T/T) (Fig. 6c, left panel). All *PEG10* RNA reads (100%) in fetal trophoblast and endothelial cells harbored the paternal allele (A). Interestingly, *PEG10* transcripts in maternal immune and endothelial cells harbored the same paternal *PEG10* allele (A), indicating their fetal origin (Fig. 6c, right panel). Collectively, these results indicate cell-to-cell transfer of *PEG10* transcripts from trophoblast to other cells. The persistent monoallelic expression of the paternal allele indicates that DNA hypomethylation at the *PEG10* loci in PE results from defective de novo DNA methylation or H3K27me3 re-establishment, rather than germline imprinting defects in the maternal gamete.

PEG10 VLPs are known to be capable of carrying other RNA transcripts, bound by PEG10 on their UTRs, into other cells. We hence identified a series of putative PEG10 VLP “cargo genes”, which were characterized by the presence of RNA without an accessible genomic locus in endothelial cells (“Methods,” Fig. 6d–f and Additional file 8: Table S7). Similarities between the 3′ and 5′ UTRs of these cargo genes (0.68, *Z* = 4.3) suggested that they may be bound by a similar RNA-binding protein (Fig. 6d and Additional file 1: Fig. S28). Several of these genes, such as *HTRA1*, *TIMP2*, *ASCL2*, and *CSH1*, were known to be functional in placentation and/or could be transferred to cultured trophoblast by trophoblast debris in ex vivo culture medium [88], suggesting that cargo genes shuttle from trophoblast to both maternal and fetal endothelial cells via PEG10 VLP in PE placentas (Fig. 6d), similar to cell-free trophoblast “debris” described in a previous in vitro study [88].

We performed Pearson correlation analysis of the cargo transcript RNA expression profiles between endothelial cells and trophoblast (Additional file 1: Fig. S29) to determine whether PEG10 VLP-mediated RNA cargo transfer between cells was possible. Although we observed a high correlation between VCT/VCTp and early endothelial cell populations in control placentas, we also observed similarity between terminally differentiated trophoblast (EVT and SCT) and endothelial cells in PE (Additional file 1: Fig. S29), indicating increased cell-to-cell transfer of PEG10 VLP in PE. Collaboratively, these results suggest that PEG10-containing VLP production is amplified in PE, which results from defective de novo DNA methylation in trophoblast. The majority of transcriptional alterations in endothelial cells in PE result from the trans-cellular transfer of RNA molecules from trophoblasts, potentially carried by PEG10 VLPs.

### Excessive PEG10 disrupts maternal artery development to phenocopy arteriovenous malformation (AVM)

**Fig. 7:**
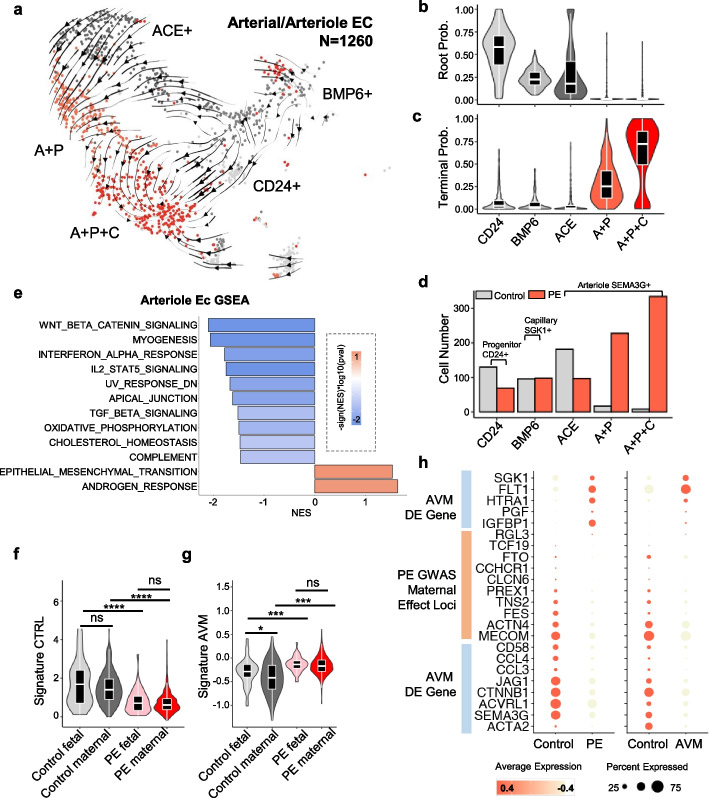
Excessive PEG10 disrupts maternal artery development to phenocopy AVM. **a** Single-cell evolution trajectory inferred by RNA velocity of arterial/arteriole endothelial cells (Ec), projected on UMAP space. Arterial Ec evolve from the CD24 + cluster (Additional file 1: Fig. S2b) into BMP6 + (SGK1 + , capillary, Additional file 1: Fig.S2b) cluster or ACE + (SEMA3G + , arteriole, Additional file 1: Fig. S2b) cluster. ACE + populations. ACE + population give rise to two ACE + /PAPPA2 + populations that differs by CLIC3 expression. **b** Root and **c** terminal cell probability of single cells in each Ec cluster shows that the PAPPA2 + clusters of Ec are *induced* in PE. **d** Cell number of each Ec cluster in control and PE placenta, showing that the PAPPA2 + clusters are PE-specific. **e** Normalized enrichment score (NES, *x* axis) of gene pathways with differential activity between PE and control ACE + population in GSEA analysis. Only significantly different pathways (raw *P*-values < 0.05) were shown. Differential activities are shown with color (red: upregulation in PE; blue: downregulation in PE). **f** Non-arteriovenous malformation (AVM) brain endothelial signature (Signature control) expression in fetal and maternal-originated placental Ec in PE and control. **g** AVM signature [89] expression in fetal and maternal-originated placental Ec in PE and control. *P*-values were tested by Wilcox test, not adjusted, *: *P* < 0.05; ****: *P* < 2.2*10e − 6. **h** Gene expression of AVM-associated genes, including the hereditary AVM pathogenic gene ACVRL1 which interacts and is inhibited by PEG10, and PE-associated genes by GWAS study [15] in PE/control placenta Ec (left panel) or AVM/control brain Ec (right panel). *Abbreviation*: A + P: ACE2 + , PAPPA2 + ; A + P + C: ACE2 + , PAPPA2 + , CLIC3 +

In PE, vascular development in both the fetus and mother was disrupted. Specifically, the remodeling of maternal uterine spiral arteries was incomplete, and fetal capillary vessels were underdeveloped, thereby leading to placental villus that was devoid of blood circulation. We analyzed angiogenesis by delineating the developmental trajectory of arterial endothelial cells by RNA velocity [66] (Fig. 7a and Additional file 1: Fig. S2a). The results revealed that an arterial CD24 + cluster served as a progenitor cell population with heightened cell division activity (Fig. 7b,c and Additional file 1: Fig. S30a-b) that differentiated into BMP6 + /SGK1 + capillaries (“BMP6” in Fig. 7a–d and Additional file 1: Fig. S2a-b; “Capillary” in Additional file 1: Fig. S30) or ACE + /SEMA3G + arteriole cells (“ACE,” “A + P” [ACE and PAPPA2], “A + P + C’ [ACE, PAPPA2, and CLIC3] in Fig. 7a–d and Additional file 1: Fig. S2a-b; “Arteriole” in Additional file 1: Fig. S30a-b). In PE, two novel arteriole PAPPA2 + endothelial cell clusters, namely arterial A + P and arterial A + P + C, emerged (Fig. 7b). RNA velocity transfer probability analysis revealed that the root cell probability was high in arterial CD24 + and BMP6 + endothelial cells (Fig. 7c) and that terminal cell probability was high in PE-specific PAPPA2 + cells (Fig. 7d). Although the cell cycle activity of the progenitor cells was higher than that of the capillary and arteriole endothelial cells in control, the cell cycle activity of capillary and arteriole endothelial cells was significantly elevated in PE (Additional file 1: Fig. S30b). Together, these results suggest the occurrence of induced differentiation of endothelial cells into a PAPPA2 + state in PE.

PEG10 protein product RF2 inhibited the TGF-beta co-receptor ACVRL1 (ALK1) and TGF-beta signaling in vitro [90]. In vivo, PEG10 knockout resulted in placenta development failure [85] and defective fetal vascular development [91, 92]. As TGF-beta signaling is essential for angiogenesis, and mutation in these genes results in major vascular developmental defects, such as congenital cardiovascular malformation or arteriovenous malformation [93–96] (OMIM #131300, #609192, #187300, #600376, #178600, #174900, #175050), we hypothesized that excessive PEG10 transferred to maternal endothelial cells may disrupt its development by affecting the TGF-beta signaling pathway. Indeed, gene set enrichment analysis of differentially expressed genes in the arteriole indicated significant attenuation of TGF-beta signaling, together with Wnt, IL-2, and IFN-a signaling in arteriole endothelial cells in PE (Fig. 7e and Additional file 1: Fig. S31). Meanwhile, EMT activity and androgen signaling pathway were upregulated in arteriole endothelial cells in PE (Fig. 7e).

TGF-beta signaling is not only implicated in vascular development but is also known to be modulated by trophoblast-derived molecules. For example, *MMP9/MMP2* is specifically expressed in EVT and SCT (Additional file 1: Fig. S7a) and regulates TGF-beta signaling intensity [97]. To validate TGF-beta signaling defects in endothelial cells in PE, we took advantage of the interesting duality of vascular malformation disease, namely AVM, and PE. AVM is also caused by loss-of-TGF-beta signaling [93–95]. Mutation in either endoglin (*Eng*) [12], an accessory TGF-beta receptor, or its co-receptor *ACVRL1* [98], which interacts with and is inhibited by PEG10 [90], causes AVM. *Eng* is known to be implicated in PE; excessive solutable endoglin (s-Eng) has been observed in the serum of patients with PE [21, 23, 24], and the overexpression of s-Eng has been observed to result in a PE-like phenotype in animal models [99]. Single-cell analysis of brain vascular endothelial cells has revealed similar downregulation of TGF-beta signaling in AVM arteries [89, 100]. We thus compared the gene expression profile between AVM and PE. Fetal and maternal endothelial cells exhibited an AVM-like gene expression pattern, i.e., the downregulation of control-specific arteriole genes and the upregulation of AVM-specific gene activities (Fig. 7f, g).

The mirroring of gene expression profile between PE and AVM arterial endothelial cells was found across individual AVM-specific and control-specific genes, as well as the expression of GWAS-associated genes of PE (Fig. 7h), including *MECOM*, a key endothelial TF that is significantly associated with PE [101–103]. In control placentas, the AVM-specific gene activity was lower in maternal endothelial cells than in fetal endothelial cells (Fig. 7g). Considering that PEG10 was produced by fetal endothelial cell-proximal VCT but not the maternal endothelial cell-proximal SCT/EVT in control, these results collectively suggest that PEG10 induced TGF-beta signaling switch between maternal and fetal endothelial cells to divert differential developmental trajectories for fetal and maternal blood vessels during placentation. The placental villous tree, which serves as an arteriovenous shunt between the remodeled maternal spiral artery and draining vein [104], is innervated by fine fetal capillaries [105, 106]. Coordinated enlargement of spiral artery opening and miniaturization of fetal capillary requires differential angiogenic signaling. Although the loss of PEG10 results in the collapse of the fetal capillary vessel in villous [92], our results revealed that maternal vascular remodeling was affected by the fetal overexpression of *PEG10*.

### PEG10 perturbs endothelial cell proliferation and function by negatively regulating TGF-beta signaling

We experimentally validated whether PEG10 affects endothelial cell biology via TGF-beta pathway in a human endothelial cell line (HUVEC), which has basal PEG10 expression (Additional file 1: Fig. S32). Overexpression of PEG10 increased the PEG10 RNA level up to 20-fold compared to control (Additional file 1: Fig. S32a) and elevated PEG10 protein expression (Additional file 1: Fig. S32b). Knockdown of PEG10, on the other hand, significantly downregulated PEG10 on both transcriptional (Additional file 1: Fig. S32c) and protein levels (Additional file 1: Fig. S32d).

**Fig. 8:**
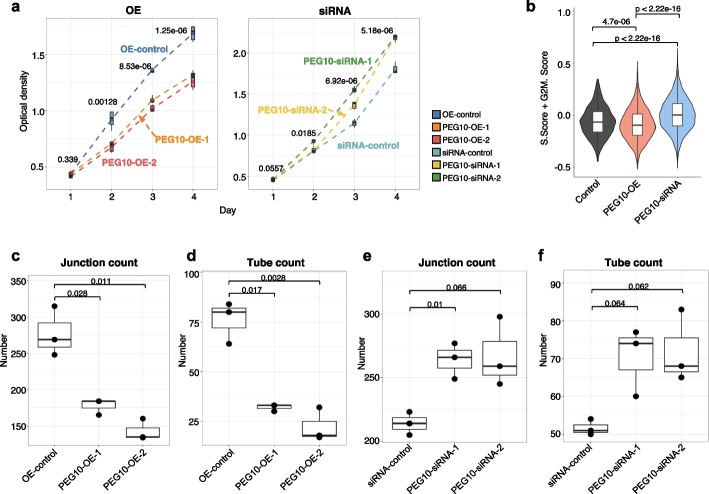
PEG10 overexpression negatively impacts endothelial cell proliferation and function. **a** Optical density in MTT assay of PEG10 in HUVEC cells transfected with control vector (OE-control) or PEG10-overexpression (PEG10-OE-1, PEG10-OE-2) vectors (left), or HUVEC cells transfected with control scrambled siRNA (siRNA control) or PEG10-targeting siRNA (PEG10-siRNA-1, PEG10-siRNA-2) (right). *P*-values were examined by *t*-test and adjusted with “BH” method. **b** Cell cycle-related gene expression score (S and G2/M) in PEG10 RNAi, control, and PEG10 overexpression (OE) cells by single-cell RNA sequencing. **c** Junction count in tube formation assay for HUVEC cells transfected with control or PEG10-overexpression vectors. **d** Tube count in tube formation assay for HUVEC cells transfected with control or PEG10-overexpression vectors. **e** Junction count in tube formation assay for HUVEC cells transfected with control or PEG10-targeting siRNA. **f** Tube count in tube formation assay for HUVEC cells transfected with control or PEG10-targeting siRNA. *P*-values were examined by *t*-test, not adjusted

In the MTT assay, overexpression of PEG10 inhibited, while knockdown of PEG10 accelerated HUVEC cell proliferation over a course of 4 days (Fig. 8a). In concordance to this, scRNA sequencing of the same cells showed increased cell cycle gene set activity in PEG10 knockdown cells compared to control cells or PEG10 overexpression cells (Fig. 8b). Furthermore, PEG10 overexpression decreased tube formation activity (Fig. 8c,d), while PEG10 knockdown increased the tube formation activity of HUVEC cells compared to control cells (Fig. 8e,f). PEG10 overexpression decreased the tube formed by ~ 3 folds and junction formed by ~ 2 folds (Fig. 8c,d). On the contrary, PEG10 knockdown increased the tube formed by 50%, and junction formed by ~ 25% (Fig. 8e,f).

**Fig. 9:**
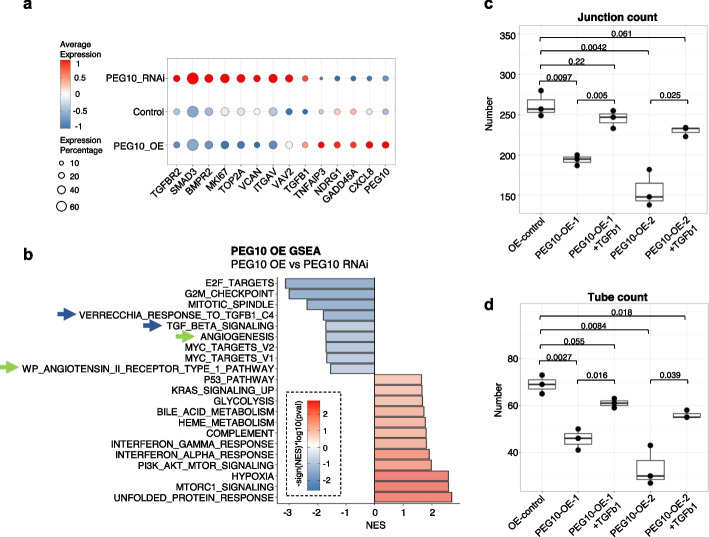
PEG10 perturbs endothelial cell biology by negatively regulating TGF-beta signaling. **a** Differential gene expression between PEG10 overexpression (OE), PEG10 RNAi, and control cells. **b** Differential gene set enrichment activity between PEG10 OE and PEG10 RNAi cells. Blue arrows indicate TGF-beta signaling and green arrows show angiogenesis pathways. **c** The reduction of tube junction count in PEG10 overexpression cell is rescued by TGF-beta supplementation. **d** The reduction of tube count in PEG10 overexpression cell is rescued by TGF-beta supplementation. *P*-values were tested by *t*-test, not adjusted

In concordance to the cell proliferation and function experimental result, PEG10 overexpression cells showed increased expression of cell cycle arrest-related gene such as *GADD45A*, and decreased expression of proliferation genes such as *MKI67* and *TOP2A* (Fig. 9a). Notably, the TGF-beta signaling pathway components *TGFB1*, *TGFBR2*, and *SMAD3* are upregulated in PEG10 knockdown cells (Fig. 9a). Gene set enrichment analysis showed that compared to PEG10 knockdown cells, PEG10 overexpression cells showed downregulated activities in cell proliferation (G2M-checkpoint, Mitotic spindle, E2F targets), TGF-beta-signaling, and angiogenesis pathways (Fig. 9b). The expression of angiogenesis and endothelia-related genes, such as *SGK1*, *GDF15*, and *ACTN4* are disrupted by PEG10 perturbation (Additional file 1: Fig. S33), suggesting possible downstream effector of PEG10-TGF-beta signaling and mimicking the phenotype observed in vivo for PE endothelial cells.

We then tested whether treating PEG10 overexpression cells with TGF-beta-1 protein could rescue their biological function. Compared to control condition, TGF-beta-1 protein restored tube growth and junction formation in the PEG10 overexpression cells (Fig. 9c, d). In general, TGF-beta-1 protein significantly improved junction and tube formation number in the PEG10 overexpression cells (Fig. 9c,d), which restored junction and tube formation in PEG10 overexpression cells to a level not significantly different from the control cells (Fig. 9c,d). Together, these results showed that PEG10 disrupts endothelial cell biology via negatively regulating TGF-beta signaling.

## Discussion

PE is clinically characterized as defective development of the placental villous tree, which is formed by trophoblasts [4, 5], the failure of uterine spiral artery remodeling, and deterioration of the endothelial vasculature [107]. Our results revealed a molecular account of PE pathogenesis.

### Epigenetic mechanisms regulating trophoblast development in PE

The uterine spiral artery is the terminal branch of the uterine artery network and undergoes structural remodeling during implantation. The invasion of the spiral artery by EVTs blocks blood flow and replaces the inner wall of endothelial cells, resulting in the dilation of blood vessel termini [108]. The encroachment of the villous tree into the uterine cavity thus functions as an arteriovenous shunt between the uterine spiral artery (“arteriole”) and venous system [104], with notably reduced blood flow velocity and increased total blood flow perfusing the villi that enable efficient gas and nutrient exchange [109]. In PE, the villous tree is underdeveloped and many terminal villi lack capillary [4, 5]. This is accompanied by reduced spiral artery remodeling in the maternal myometrium [6, 7].

By comparing individual cells derived from the placenta of PE and normal pregnancies, we corroborated and expanded upon our prior findings regarding alterations in cellular composition in PE [4, 7, 110–113]. Additionally, we identified previously unknown endothelial cells that are associated with PE. Particularly, we determined that in PE, defective epigenomic reprogramming including reduced de novo DNA methylation specific to extraembryonic tissue and dysregulated H3K27me3 modification coordinately stall the development of the trophoblast.

De novo DNA methylation of extraembryonic tissue-specific gene promoter has been extensively documented [50]. Our transcription network analysis in conjunction with DNA methylation profiling revealed that the PE phenotype may be pre-determined at the preimplantation stage as de novo DNA methylation occurs in the blastocyst stage. Additionally, we identified PE trophoblasts showing defective TF activities are primarily early-stage trophoblast progenitor cells and VCT but not terminally differentiated SCT and EVT. Hence, these findings suggest that an early imbalance of growth factor signaling between the inner cell mass and trophectoderm results in defective placenta development. Additional investigations on organoids are necessary to clarify the precise molecular mechanisms involved in this process.

### Retrovirus in placenta development

The emergence of placental structures is accompanied, and probably driven, by retrovirus domestication. Retroviruses exploit the placenta as their waypoint of trans-generational infection [114]. Due to host–pathogen co-evolution, most endoretrovirus genes are silenced during the adult stage through DNA methylation [115, 116]. However, these genes are only transiently activated during embryogenesis owing to global demethylation [42]. The genes and products of endoretroviruses are often expressed in the placenta [117–119], with the promoter of retroviral genes serving as species-specific enhancer elements that are specifically active in the placenta [120–123]. The incorporation of endoretrovirus-encoded envelop proteins, such as Syncytin I and II, promotes trophoblast fusion, resulting in the formation of multi-nuclei giant cells that line the maternal–fetal interface [124–129]. Interestingly, various animal taxa have exhibited the incorporation of envelop proteins from various retroviruses, suggesting convergent selection of retrovirus-gene-mediated placental features [117, 124, 130–138]. The incorporation of retroviral genes into trophoblasts probably facilitates not only the cell–cell fusion but also the invasiveness of trophoblasts, as well as immune suppression on the maternal side.

The adaptation of the placenta results in an active battleground for parental genes. Theoretically, such conflict may arise due to zero-sum-like competition between the mother and the fetus, such as for the nutritional supply. In the loci regulating such effects in the fetal genome, the paternal allele exhibits a proclivity towards fetal growth, whereas the maternal allele tends to protect the mother. Furthermore, the paternal endoretrovirus allele experiences reduced selection pressure for expression because it facilitates transmission of itself, in theory, to sibling littermates or the maternal body [139–141]. Examples that align with these theories include the LTR12 family of endoretrovirus, which are hypomethylated in the sperm and undergo ZGA-specific transcription. The transient expression of LTR12 endoretrovirus loci suggests a tight control over its chromatin accessibility, probably during the re-establishment of the global DNA methylation landscape. Although the functions of LTR12 endoretrovirus remain unclear, they may serve as an alternative 5′ promoter for downstream genes in certain cases [142]. On the contrary, two paternally imprinted endoretroviral genes, namely *RTL1* and *PEG10*, are expressed and functional in the placenta [143].

### Epigenetic reprogramming regulates endoretrovirus expression

PEG10 protein and RNA transcript are prevalent in the PE placenta but not in control (Figs. 5 and 6). Our findings indicate that trophoblast maturation is accompanied by reduced levels of PEG10 expression (Fig. 5d), suggesting the maturation-dependent expression of PEG10 in early trophoblasts such as TSC (VCTp) and VCT. The PEG10 promoter is bound by PRC2 (Fig. 6a) and undergoes de novo DNA methylation during extraembryonic tissue development. In the PE placenta, LTR12C is commonly hypermethylated (Additional file 1: Fig. S18b), whereas PEG10 is maintained in a hypomethylated state. The imbalance of de novo methylation effect on these loci, albeit both hypomethylated at the paternal germline, may suggest a redirected de novo DNA methylation effort from one type of endoretrovirus to another.

In PE, the concomitant increase in H3K27ac and reduction in de novo DNA methylation on the *PEG10* locus in the placenta (Fig. 6a and Additional file 1: Fig. S27) is concordant with the overexpression of *PEG10* in trophoblasts, especially in the terminally differentiated EVTs and SCTs (Fig. 5b, d). Although PEG10 is essential for villous blood vessel formation, the restriction of PEG10 expression to villous cytotrophoblasts may be essential to restrict its function exclusively to the fetal side instead of the maternal side. In the placenta of normal pregnancy, fetal capillaries in the villous tree are very fine, whereas the maternal spiral artery is deformed and wide open. The spatially adjacent blood vessels thus require differential regulation of angiogenesis.

### PEG10 function in the regulation of angiogenesis via TGF-beta

PEG10 is subjected to strong purifying selection (loss-of-function [LoF] intolerance score pLI = 0.87, with only 1 LoF SNV in 249,162 sequenced alleles in gnomAD), suggesting its essentiality. In vitro analysis and gain-of-function experiments have shown that PEG10 binds to its own UTR to form VLPs [86], which can be transferred to other cells. A similar transfer of exosome-like particles between trophoblasts and HUVEC cells has been previously documented [88]. PEG10 attenuates TGF-beta signaling by interacting with TGF-beta co-receptor ACVRL1/ALK1, ultimately inhibiting its function [90]. Our results indicate that excessive PEG10 perturbs endothelial cell biology by negatively regulating TGF-beta signaling, to an extent that mirroring AVM formation. TGF-beta is a well-documented master regulator of angiogenesis. Mutations of genes in the TGF-beta pathway underlied many human vascular developmental disorders. Interestingly, in PE, s-Eng are notably elevated in the serum, suggesting that antagonism of TGFb signaling might be a common theme in regulating vascular remodeling and endothelial permeability in PE.

## Conclusions

In our research, through single-cell analysis, we discovered developmental delays and transcriptional defects in PE trophoblasts. Our multimodal epigenomic studies showed that impaired extraembryonic tissue-specific de novo DNA methylation led to delayed development in PE trophoblasts. We observed excessive immature trophoblasts in PE placentas, which significantly upregulated maternally imprinted genes, notably PEG10. This gene can produce virus-like particles that transfer between cells. While PEG10 expression is limited to fetal vessel-interacting VCT in normal pregnancy, the maternal vessel-interacting SCT and EVT overexpressed PEG10 in PE pregnancy, enabling PEG10 VLP transfer into the maternal endothelial cells to induce ACE2 + /PAPPA2 + arteriole endothelial cells. These PE-specific endothelial cells are functionally defective marked by reduced TGF-beta and Wnt signaling. They share high transcriptomic similarity with other vascular malformation diseases caused by loss-of-TGF-beta signaling such as arteriovenous malformation. They also exhibit a gene expression profile indicative of impaired endothelial zonation. These results revealed how multi-layered epigenetic mechanism controls proper placentation, and how its disruption leads to a lethal pregnancy complication.

## Methods

### Human biospecimens

This study was conducted in accordance with the measures of the People’s Republic of China on the administration of Human Assisted Reproductive Technology, the ethical principles of the Human Assisted Reproductive Technology, the Helsinki Declaration, and the internal ethic protocols of Peking University Third Hospital, Guangdong Woman and Children’s Hospital, West China Second Hospital of Sichuan University and Zhongnan Hospital of Wuhan University.

The study protocol was approved by Peking University Third Hospital Medical Science Research Ethics Committee (approval number: (2020)YiLun(310–02)), Peking University Institutional Review Board (approval number: IRB0001052-16009), Institutional Review Board of Guangdong Woman and Children’s Hospital (approval number: YiLun[201701044]), Ethics Committee of the West China Second Hospital of Sichuan University (approval number: YiXueKeYan2020(064)) and Institutional Ethics Committee of Zhongnan Hospital of Wuhan University (approval number: 2015029 and 2020102). Informed consent was obtained from the donors and their guardians. The study protocol was approved by the Institutional Review Board (IRB) of Peking University Third Hospital ((2020)YiLun(310–02) and IRB0001052-16009), IRB of Guangdong Woman and Children’s Hospital (YiLun[201701044]), IRB of West China Second Hospital of Sichuan University (YiXueKeYan2020(064)), and IRB at Zhongnan Hospital of Wuhan University (approval number: 2015029 and 2020102). The human sample preservation by Department of Biological Repositories, Zhongnan Hospital of Wuhan University (official member of the International Society for Biological and Environmental Repositories-International Repository Locator, https://irlocator.isber.org/details/60) was approved by the Ethics Committee (approval number: 2017038) and China Human Genetic Resources Management Office, Ministry of Science and Technology of The People’s Republic of China (approval number: 20171793).

For 10 × single-cell RNA, single-cell ATAC, and immunostaining experiments, placenta samples were collected from Peking University Third Hospital and West China Second Hospital of Sichuan University. For placenta ATAC-seq and CUT&Tag experiments, placenta samples were collected from Peking University Third Hospital. For placenta methylation capture sequencing experiments, placenta samples were collected from Peking University Third Hospital and Guangdong Woman and Children’s Hospital. FFPE tumor or normal tissue slides were collected from Zhongnan Hospital of Wuhan University, Wuhan, China.

Clinical assessment of human (pregnant female) phenotype was done according to ACOG guideline of hypertension in pregnancy [144]. Routine laboratory tests and pathology assessments were done according to the relevant Chinese clinical protocols.

Placentas were collected within 1 h of labor and transferred to laboratory in high glucose + 10% FBS in DMEM. Placenta samples were resected in PBS from the maternal or fetal surface of placenta, 2 cm*1 cm in size, and within 4–5 cm from the root of umbilical cord. For single-cell sequencing, CUT&Tag, or ATAC-seq, villous was further manually dissected from the maternal surface placenta with a pair of fine forceps for further processing. For methylation sequencing, placenta samples were flash frozen in liquid nitrogen and stored at − 80 °C.

### Cell culture

Human umbilical vein endothelial cells (HUVECs) were obtained and tested for mycoplasma non-contamination from the Chinese Academy of Sciences Cell Bank (Shanghai, China). The identification of the HUVEC cell line was conducted at the China Centre for Type Culture Collection in Wuhan, China. The cells were cultured and maintained in MEM medium supplemented with 10% fetal bovine serum and 1% penicillin/streptomycin with 5% CO_2_ at 37 °C.

### siRNA and plasmid transfection

The PEG10-target specific siRNA and negative control siRNA were purchased from Shanghai GenePharma Co., Ltd. The sense sequence of the siRNAs were as follows: PEG10 siRNA-1 (5ʹ-CCCACUACCUGAUGCACAATT-3ʹ), PEG10 siRNA-2 (5ʹ-GCACUCGAUCUAUCGUCUUTT-3ʹ), and negative control siRNA (siRNA control) (5ʹ-UUCUCCGAACGUGUCACGUTT-3ʹ). The plasmids for human PEG10, OE-PEG10 (or PEG10 OE-1) was synthesized based on the sequence of NM_001172437.2 by GenScript Biotech while OE-PEG10-2ORF (PEG10 OE-2) was synthesized based on the sequence in Segel et al. [86] by GenScript Biotech. Two plasmids encode the same PEG10 amino acid sequence. Negative control vector for overexpression is pCMV-GFP-HA.

HUVECs were transfected with siRNA or plasmids using Lipofectamine 3000 Transfection Kit (L3000-015, Invitrogen, USA), according to the manufacturer’s protocol. The gene silencing efficiency and overexpression efficiency of transfected cells were confirmed by qRT-PCR and Western blot.

### Quantitative real-time PCR (qRT-PCR)

HiPure Total RNA Mini Kit (R4111-03, Magen, China) was utilized to extract total RNA from cell lines. The ReverTra Ace qPCR RT Kit (FSQ-101, Toyobo, Japan) was used for the reverse transcription. Five-hundred-nanogram cDNA templates were added to a PCR system with a final volume of 20 μl. The primer sequences are listed in the following table:

**Table:** 

Gene	Forward primer (5′–3′)	Reverse primer (5′–3′)
GAPDH	CTGGGCTACACTGAGCACC	AAGTGGTCGTTGAGGGCAATG
PEG10	GAGCACCAGGGATTTCTCAGT	GGTAGTTGTGCATCAGGTAGTG

### Western blot analysis

The cells were lysed in RIPA buffer (containing protease inhibitor and phosphatase inhibitor) on ice for 30 min. The cell lysates were centrifuged at 12,000* g* for 15 min and the supernatant was collected. For Western blots, total protein was separated using 10% SDS-PAGE gels, then transferred to PVDF membrane. Membranes were blocked with 5% skim milk in TBST buffer for 2 h at room temperature. The membranes were then incubated separately with the appropriate primary antibodies, including anti-PEG10 (Proteintech Inc, China, Cat. #4412-1-AP, 1:1000 dilution for WB and anti-GAPDH (Proteintech Inc, China, Cat. #0004-1-Ig, 1:5000 dilution for WB), for overnight at 4 °C. After washing three times with TBST buffer, membranes were incubated with secondary antibodies, including goat anti-mouse IgG (H + L) HRP (Sungene Biotech, China, Cat. #LK2003, 1:5000 dilution for WB) and goat anti-rabbit IgG (H + L) HRP (Sungene Biotech, China, Cat. #LK2001, 1:5000 dilution for WB), for 2 h at room temperature. Bands were detected using the Clarity™ Western ECL Substrate kit (1705061, Bio-Rad, USA), and images of blots were taken by the BioSpectrum 515 Imaging System (UVP, USA).

### MTT assay

The transfected cells were seeded in 96-well plates at a density of 3000 cells per well. After incubation of 1–4 days, each well was added 20 μL 5 mg/mL MTT (M5655, Sigma, USA) for 4 h at 37 ℃. The medium was removed and 150 μL of DMSO was added to dissolve the formazan precipitate in the 96-well plate. Finally, the absorbance of each well at 570 nm was tested using a microplate reader (Molecular Devices, USA).

### Tube formation assay in vitro

Tubule formation assays were performed as previously described [145]. The 96-well plates were coated with 50 µL of Matrigel (40183ES10, YEASEN, China) per well and incubated for 1 h. The transfected HUVECs (1.8 × cells per well) were seeded on Matrigel and cultured for 6 h. The tube formation of HUVECs was observed and photographed using a microscope, and the counts of tubes and junctions were analyzed by ImageJ.

For evaluating the effect of TGFb1 treatment on the tube formation, the transfected HUVECs were pretreated with 5 ng/mL TGFb1 (HZ-1011, Proteintech, China) for 12 h, and then seeded on the Matrigel for tube formation assay described above.

### Statistical methods

Clinical phenotypes were summarized as mean (range lowest–highest) or mean (percentage). All statistics for clinical phenotype was done with t-test (two-sided) or Wilcoxon Rank test (two-sided) if t-test was not applicable. Detailed statistical methods were briefly denoted in the figure legends or text accompanying. All statistical analyses in this study were performed using R (3.6.2) (http://CRAN.R-project.org).

### Single-cell RNA and chromatin accessibility sequencing

Fresh tissues were processed immediately after being obtained from donors. Tissues were cut into tiny pieces (< 1 mm diameter) and then subjected to dissociation using collagenase II (Biofrox Ltd., #2275MG100) and 100 μl of DNase (Servicebio Ltd., #1121MG010) at 37 °C for 1 h. After dissociation, cells were filtered with 40 μm BD filter mesh and subsequently centrifuged at 250* g* for 5 min. Cell pellets were washed in PBS twice and resuspended in 1 ml ice-cold RBC lysis buffer and incubated at 4 °C for 10 min. Ten milliliters of ice-cold PBS was added to the tube and subsequently centrifuged at 250* g* for 10 min. After decanting the supernatant, the pellet was resuspended in 5 ml of calcium- and magnesium-free PBS containing 0.04% weight/volume BSA. Cells were counted using Trypan blue (Solarbio, Beijing, China). For chromatin accessibility sequencing, approximately 10^6 cells were used for nucleus extraction. Nucleus extraction were performed as 10 × single-cell library preparation was done according to the manufacturer’s protocol. Chrominum Single Cell 3' V3 kits and ATAC V2 kits were used. For RNA sequencing, single-cell suspensions were loaded onto a Chromium Single-Cell Controller Instrument (10 × Genomics) to generate single-cell gel beads in emulsions (GEMs) targeting ~ 8000 cells. After generation of GEMs, reverse transcription reactions were engaged to generate barcoded full-length cDNA, which was followed by disruption of emulsions using the recovery agent, and then cDNA clean-up was performed with DynaBeads Myone Silane Beads (Thermo Fisher Ltd.). Next, cDNA was amplified by PCR. Subsequently, the amplified cDNA was fragmented, end-repaired, A-tailed, and ligated to an index adaptor, and then the library was amplified. The scRNA libraries were sequenced aiming to have ~ 5000 reads per cell on Illumina Novaseq6000 with paired-end 150-bp reads. Sequencing was performed at Berry Genomics, Beijing, China. QC metrics is described in Additional file 9: Table S8.

For ATAC analysis, tagmentation was performed according to the manufacturer’s protocol. After tagmentation reaction, nucleus suspensions were loaded a Chromium Single-Cell Controller Instrument (10 × Genomics) targeting ~ 10^4 nucleus in one reaction. After generation of GEMs, PCR reaction were performed to amplify the library. DNA clean-up was performed with size-selection XP beads. Libraries were sequenced aiming to have ~ 5000 reads per cell on Illumina Novaseq6000 with paired-end 50 bp reads. Sequencing was performed at Berry Genomics, Beijing, China. QC metrics is described in Additional file 9: Table S8.

### Shared single-cell profiling of RNA and chromatin accessibility (SHARE-seq)

SHARE-seq experiment was performed with cryo-preserved transfected HUVEC cell line samples. The experiment was performed as described previously [146] with minor modifications: (1) After fixation, nuclei were isolated with Nuclei Lysis Buffer (10 mM Tris HCl pH 7.4, 10 mM NaCl, 3 mM MgCl_2_, 0.1% Tween20, 0.1% NP40, 0.01% Digitonin, 0.75%BSA) on ice for 10 min; (2) Input 20,000 nuclei for ATAC Tn5 tagmentation per reaction; (3) Samples were multiplexed by using different R1-barcode in one single SHARE-Seq reaction. (4) ATAC library was purified with QIAquick Gel Extraction Kit (Cat. 28704, Qiagen); (5) Input 150 ng cDNA for RNA library tagmentation and purified with 0.7 × AMPureXP beads (Cat. A63880, BECKMAN COULTER); (6) Each ATAC or RNA library from one single SHARE-Seq reaction was sequenced with MGI2000 sequencer with PE150 format to target approximately 500 M raw reads on average. Sequencing was performed at Euler Technology, Beijing, China. QC metrics is described in Additional file 9: Table S8.

### Chromatin accessibility sequencing (ATAC-seq) on bulk placenta tissue

Twenty-milligram flash-frozen placenta tissue samples were minced using a double-sized douncer (Sigma, #D8938) in 1xHB (0.25 M sucrose, 0.06 M KCl, 0.005 M MgCl_2_, 0.015 M NaCl, 0.01 M Tris HCl pH 7.5), added to 5 ml trypsin and 40 μl 5U/μl DNase I (Sigma, #D5025) and digested in 37 °C for 45 min, with two times of rotation in between to mix the reaction. The digested cells were then neutralized with equal volume DMEM (Thermo Fisher, #11995065) plus 10% FBS (Gibco, #16000044) and filtered through a 70-μm cell filter (BD Falcon, #352350). The homogenized sample was centrifuged at 500* g*, 4 °C for 5 min. The sedimented cells were then resuspended in 400 μl 1xHB and washed once, transferred to 2 ml LoBind Tube (Eppendorf), and washed again. Cells were counted using Trypan blue (Solarbio, Beijing, China). After quantification, the cells were then added to a 30%-40%-50% iodixanol (Sigma, #D1556) gradient and centrifuged at 3000* g*, 20 min at 4 °C. The cell layer at 30%-40% interface was collected for library preparation. DNA library were prepared with a Tn5 transposase kit (Vazyme, #TD501) using 1 million cells per reaction according to the manufacturer’s protocol. After Tn5 transposition and PCR amplification, the sequencing library were quality-controlled with SYBR-green-based qPCR using primers for house-keeping gene (*GAPDH*) promoter and gene desert (chr5: 105187030–105190000) before sequencing. Each library was sequenced to 30 M reads on Novaseq6000 sequencer (Illumina, CA). Sequencing was performed at Berry Genomics, Beijing, China. QC metrics is described in Additional file 9: Table S8.

### CutAndTag sequencing on dissociated cells

Digestion of placenta tissue follows the same protocol with bulk ATAC assay. After quantification, 50,000–100,000 cells were used for CutAndTag experiment. CutAndTag experiments were performed with NovoProtein CutAndTag 2.0 pAG-Tn5 kit (NovoProtein, #N259) according to the manufacturer’s protocol. Antibodies used in this study include the following: anti-H3K4me3 (Diagenode, #C154100003), anti-H3K27ac (Abcam, #ab4729), anti-CTCF (Abcam, #ab188408), anti-Histone H2A.Z (Abcam, #ab4174), anti-H3K27me3 (Abcam, Cat. #ab6002), goat anti-mouse IgG (Sangon, #D111024), and goat anti-rabbit IgG (Sangon, #D111018). Each library was sequenced to 2 × human genome coverage on Novaseq6000 sequencer (Illumina, CA) in PE150 format. Sequencing was performed at Berry Genomics, Beijing, China. QC metrics is described in Additional file 9: Table S8.

### Nucleic acid preparation for DNA and methylation sequencing

Tissue genomic DNA was extracted from oral swab or placenta tissue with Qiagen Animal Tissue DNA Extraction Kit (Qiagen, #69504) according to the manufacturer’s protocol. Genomic DNA from FFPE tissue slides were extracted using MagPure Tissue DNA DF Kit (Magen Inc., #MD5112-TL-06). Extracted DNA were quality-controlled by Qubit dsDNA HS assay (Thermo Fisher Ltd.) and Agilent 2100 Fragment Analyzer.

### Single-stranded DNA methylation capture sequencing

Genomic DNA (200 ng) were bisulfite converted using EZ-DNA Methylation-Gold Kit (Zymo, #D5006) according to the manufacturer’s protocol. After conversion, the DNA were subjected to a single-stranded library preparation protocol Tequila 7N (Euler Technology). In brief, the DNA were end-repaired using Klenow (NEB) and tailed with poly-A using TdT (Takara), ligated to a poly-T overhang adaptor using T4 DNA ligase (Enzymatics), and linearly amplified for 12 cycles using PhusionU (Thermo Fisher Ltd.). The linear products were then annealed to a 5' adaptor with 7 bp 3' random nucleotide overhang and amplified using adaptor oligos (Sangon, Shanghai, China) with Phusion (Thermo Fisher Ltd.), resulting in a library with proper Illumina sequencing adaptor ends ready for NGS. Hybridization was done with SeqCap EpiGiant Enrichment Probe (Roche, #07138911001), oligos and SeqCap wash and binding buffers (Roche) following the manufacturer’s protocol. After hybridization, the library was amplified using Phusion (Thermo Fisher Ltd.) for 8 cycles and sequenced on Novaseq6000 sequencer (Illumina, CA) to 100 M PE150 reads. Sequencing was performed at Berry Genomics, Beijing, China. QC metrics is described in Additional file 9: Table S8.

### Genomic region lift-over

For genomic regions lift-over between UCSC GRCh38, GRCh37, mm9, and pantro2, the R package easyLift (version: 0.2.1, https://github.com/caleblareau/easyLift), the lift-over executable from UCSC Kent Utility (https://genome.ucsc.edu/cgi-bin/hgLiftOver) and the lift-over synteny chain files from UCSC Genome Browser were used.

### Genomic element annotation

Genomic element annotation was done using ANNOVAR (http://annovar.openbioinformatics.org/) and bedtools (https://bedtools.readthedocs.io), where applicable. Differences on any class of genomic element were computed using Fisher’s exact test.

### Single-cell RNA data preprocessing and cell clustering

Loompy-Kallisto [147] was used for mapping the RNA data for gene expression analysis. Loom files were read in R by hdf5r (https://cran.r-project.org/web/packages/hdf5r/index.html) and preprocessed with Seurat 3.2.2 [61]. Quality control was done for every single sample individually to filter against gene counts, UMI counts, total reads, and mitochondrial reads. Generally, cells with > 10% mitochondrial reads, or with UMI < 600 or > 5000, or with gene counts > 5000 were filtered prior to subsequent analysis. Such quality control process might iterate at every subsequent step to ensure the stringency of analysis. Individual samples were processed through the Seurat pipeline. Data firstly passed DoubletFinder (2.0.3) [148] with standard parameters to filter against potential doublets. The filtered data were then normalized (Log Normalization by Seurat::NormalizeData), and top 2000 variable genes were identified. Ribosomal proteins, heat shock proteins, and chaperones were intentionally removed from the variable gene list because of the highly inconsistent nature of their behaviors between different tissue types. Gene expression profile were then scaled and reduced by PCA using Seurat. Generally, ≥ 30 PCA components were included in subsequent steps. FindClusters function were initially performed using a high resolution then gradually lowered to ensure the final clusters are less than PC components. SingleR [149] annotation with human reference and conventional markers were used to initially categorize the cell clusters. Differentially expressed (DE) genes were identified with Seurat FindAllMarkers function with Wilcoxon test for the cell clusters. Comparison of the found DE genes with conventional markers was performed to ensure that clusters contain relatively pure cell population. Cell type-annotated cells were then separated into different subsets based on their types. Detailed cell types were classified manually according to known canonical markers and comparison with reference single-cell datasets. In this study, trophoblasts, endothelial cells, fibroblasts, CD8 T cells, CD4 T cells, NK cells, B cells, and myeloid cells were considered: “pools” for subsequent analysis. After preprocessing, similar type of cells from different samples were merged and re-analyzed. Quality control, doublet identification, dimensionality reduction, cluster identification, differentially expressed gene identification, and cell type identification were iteratively performed on these “pure” sets of cells. In such setting, cell type composition between samples were relatively homogeneous and usually it is unnecessary to perform data integration. When sample-driven variation is evident or in cases when dataset contains samples collecting from different sources, to control against technical variations, batch effect removal was performed using Harmony [60] with (vars.to.regress = ‘source’) option during data scaling. Contaminant cells which mis-segregated into large pools were identified and put back into the unprocessed pool. Iterative processing of cells was done semi-automatically until all cells were processed.

### Cell cycle scoring

Single-cell RNA cell cycle gene set activity measurement and cell cycle classification was done with CellCycleScore function in Seurat [61] using the `cc.gene.2019` dataset as source of G2/M and S phase gene sets. For visualization, G2M.score and S.score of a single cell were added together to suggest the relative “actively proliferating” probability.

### Developmental lineage analysis

Diffusion map of single-cell RNA expression data was computed with destiny [71] (version: 3.0.1). RNA velocity analysis of single-cell RNA sequencing data was performed with scVelo [66] using Reticulate [150] in R (3.6.2) with Python3. With RNA velocity defined the root cluster, slingshot was performed on diffusion maps to produce minimally spanning tree lineages.

### Regulatory network inference with cisTarget

Velocity genes determined in RNA velocity analysis were categorized into co-regulatory modules by non-negative matrix factorization and transcriptional binding sites (TFBS) were extracted by RcisTarget [151] with the human GRCh38 -500 bp ~  + 100 bp database downloaded from cisTopic [152] (hg38_refseq-r80_500bp_up_and_100bp_down_tss.mc9nr.feather). After extraction, high-confidence co-regulator TF of regulons with NES > 3.0 were extracted from the data. Visualization of co-regulated transcription network is performed with visNetwork (https://cran.r-project.org/package=visNetwork).

### Differentiation potential assessment

Cellular differentiation potential was assessed by CytoTRACE (version: 0.1.0) [68]. Briefly, RNA expression matrix of single cells is extracted from Seurat object, with all transcripts regardless of their in-assay-variability. CytoTRACE analysis was performed on this matrix without downsampling. Per-cluster median CytoTRACE score was used as an index for the differentiation state of each cell cluster.

### Differential expression in scRNA dataset

Differential expression (Additional file 7: Table S6) between any two sets of single cells were computed with FindAllMarkers function in Seurat with Wilcoxon test as default statistical method. The Log2FC threshold was set to be 0 and minimally expressed percentile was set to 0.1 (10%). Statistically, differentially expressed gene were determined from the resulting table using adjusted *P*-value (Berfernorri method) < 0.05 and abs(Log2FC) > 0.5 as threshold.

### Define maternal or fetal origin of placental cells

Placental myeloid, endothelial fibroblast/stromal cells from both maternal and fetal origins. Maternal- or fetal-specific marker gene sets in each cell type were calculated by FindAllMarkers based on scRNA data of Vento-Tormo et al. [55]. Significant marker genes were picked by the following criteria: (1) p_adj_val < 0.001; (2) avg_logFC > 1; (3) abs(pct.1-pct.2) > 0.3 between the annotated (WGS SNV based) fetal and maternal cells, which means the difference of cell fraction that positively expressing a certain gene between the given cluster and all other clusters is at least 30%. For example, if 60% of single cells in a cluster X expressed gene Y, compared to only 20% of single cells not in cluster X expressed gene Y, then the abs(pct.1—pct.2) is 0.4. This is followed by gene set activity measurement in single cells using Seurat::AddModuleScore. In-house sequenced single cells of each cell type are classified as maternal or fetal cells according to the gene set activities mentioned above.

### Single-cell chromatin accessibility data analysis

Single-cell ATAC (chromatin accessibility, scATAC) raw reads were mapped with cellranger-atac [153]. The mapped fragment files were then processed by ArchR [154] (version:1.0.1). Quality control, doublet identification, LSI-based dimensionality reduction, clustering, gene expression inference, peakset identification, and marker peak finding were all performed in ArchR. Batch effect removal was not involved in this analysis. Basically, cells from all samples were pooled in the initial analysis, manually annotated with known markers, and separated into different subsets. The subset data were then subjected to scRNA integration in ArchR using Seurat CCA algorithm, with constrains for integration on large pools of cell type. Peaks were called for each single-cell dataset using MACS2. Chromatin accessibility on given genomic regions was also calculated in ArchR for single-cell chromatin accessibility differential analysis. Transcription factor footprinting (activity measurement) was done in ArchR with chromVAR. Co-accessible regions were calculated with ‘addCoAccessibility’ (ArchR) (maxDist = 1000000) and filtered with correlation > 0.1.

### Extraction of cell-specific reads for SNV analysis

The 10 × Genomics cellranger (4.0) pipeline was used for mapping the scRNA for SNV analysis. The scATAC data were mapped as described above. Barcodes of defined scRNA or scATAC cell groups were firstly curated from Seurat object. BAM files was then processed by Rsamtools and reads of given specific cell barcodes were fetched. SNV were analyzed using samtools mpileup and visualized using IGV.

### Definition of VLP cargo genes

Putative PEG10 cargo genes were defined as follows: (1) abs(LogFC) > 0.5 between PE PAPPA2 + endothelial cells compared to control PAPPA2-counterparts, or adjusted *P*-value < 0.01; 2. Completely inaccessible chromatin status in PE and control endothelial cells. The PE-specific PEG10 cargo genes were defined as follows: (1) LogFC > 0.5 in PE PAPPA2 + endothelial cells compared to control PAPPA2-counterparts, or adjusted *P*-value < 0.01; (2) LogFC > 0.5 in PE trophoblast cells compared to control, or adjusted *P*-value < 0.01; 3. Accessible chromatin peaks in trophoblast cells; (4) Completely inaccessible chromatin status in PE *and* control endothelial cells. For each putative cargo gene, manual visual inspection of scATAC tracks were performed to confirm the chromatin accessibility state in trophoblast and endothelial cells (Additional file 8: Table S7).

### Sequence motif in cargo UTR

5′ and 3′ UTR of all potential transcripts from cargo gene loci were extracted from reference genome (R package org.Hs.eg.db, version: 3.6.2). Motif finding and visualization was done with R package rGADEM. Motif PWM were generated with R package TFBStools and similarity was calculated using Pearson’s method. To evaluate statistical significance, background motifs were pulled from JASPAR (vertebrate non-redundant core motifs) or ORegAnno, and similarities between the background motifs and the found UTR motifs were calculated. *Z*-score deviation from the background distribution was then computed with *scale* function in R.

### Bulk ATAC, Cut-and-Run, ChIP, and CutAndTag sequencing data preprocessing

Raw paired-end open chromatin tagmentation (ATAC), Cut-and-Run, ChIP, or CutAndTag sequencing data were mapped to human reference genome GRCh38 (Cut-and-Tag) or GRCh37 (Cut-and-Run, ChIP, and ATAC) using Bowtie2 (-k 10 --very-sensitive -X 2000) (https://github.com/BenLangmead/bowtie2). All unmapped reads, non-uniquely mapped reads, reads with low mapping quality (MAPQ < 20), and PCR duplicates were removed. For CutAndTag sequencing libraries, data were used as is, because all CutAndTag libraries showed excellent concordance to reference peak sets in Encode library. As bulk placenta tissue ATAC-seq suffers greatly from cell debris containing free-floating cell-free DNA, we further filtered the libraries for quality control. The in-house ATAC-seq data were quality-controlled by assessing insertion size (using an in-house R script) and TSS enrichment (using an in-house R script with GenomicRanges package (https://github.com/Bioconductor/GenomicRanges) measuring the depth ratio at the promoter region (refFlat annotation from UCSC Genome Browser) (0 bp of TSS vs. 1kbp + / − of TSS). A QC-passed ATAC-seq library must have TSS enrichment of 6, mapped deduplicated sequencing fragments ≥ 20 M PE reads, PCB1 > 0.9, PCB2 > 3 (https://www.encodeproject.org/pipelines). Enrichment peaks were determined by intersecting peaks found from MACS2 callpeak (-f BAMPE, https://github.com/taoliu/MACS) and Genrich (-r -m 1 -j; for ATAC only; and standard parameter for CutAndTag) (https://github.com/jsh58/Genrich). Further quality control of ATAC-seq libraries including read length, V-plot, and TSS enrichment were done with custom R script and deeptools (https://github.com/deeptools/deepTools). Reliable peaks were identified with IDR (https://www.encodeproject.org/software/idr). Reliable ATAC peaks from different set of data were converged with 1 bp minimum overlap and extended to the largest width of overlapping peaks. Joining these operation results in a set of non-overlapping, varied-width peaks across the genome encompassing all reliable open chromatin region.

### Correlation of bulk and single-cell ATAC-seq datasets

Coverage (RPKM) of ATAC-seq on IDR peaks were calculated for each sample, and Pearson correlation coefficient were calculated for each pair of samples in R (3.6.2).

### Measurement of difference of bulk ATAC-seq peaks

Read coverage of sequencing library were collected over the repeatable ATAC enrichment peak mentioned above with Sambamba. Differential enrichment was performed with DESeq2 (https://github.com/mikelove/DESeq2) using standard parameters. For samples with few replicates, we adapted a general linear model approach for estimating difference following the method mentioned in Reilly et al. [155]. Differential ATAC peaks between preeclampsia and normal placenta are summarized in Additional file 5: Table S4.

### Motif finding and transcription factor footprint analysis in bulk ATAC-seq

Transcription factor binding motifs were collected from ReMap. Motifs were extracted using HOMER package (http://homer.ucsd.edu/homer/). Raw ATAC-seq data were preprocessed using RGT-Hint (www.regulatory-genomics.org/hint) package with standard parameter (rgt-hint footprinting --atac-seq --paired-end) and matched to motifs (rgt-motifanalysis matching --organism = hg19). Differential transcription factor footprint was analyzed by Hint (rgt-hint differential --organism = hg19 --bc) on three independent pairs of biological replicates between preeclampsia and non-preeclampsia placenta.

### Histone modification analysis

Histone modification peaks were identified by MACS2 using following parameters: H3K4me3/H3K4me1/H3K27ac: -g hs -nomodel -nolambda; H3K27me3: -g hs --broad --broad-cutoff 0.05 -nomodel -nolambda. Weak (summed RPKM < 20) or irreproducible peaks were removed for further analysis. Histone modification peaks overlapping known ATAC enrichment peak were used for further analysis. Read coverage of histone modification sequencing library were collected over the repeatable ATAC-and-histone-modification enrichment peak mentioned above with Sambamba, and normalized to RPKM (reads per kilo million for library), and Z-normalized.

Broad H3K27me3 regions were called by macs2 (-f BAMPE -g hs -q 0.01 --broad --broad-cutoff 0.1 --nolambda --SPMR --nomodel -B). The called regions were merged to neighboring regions within + / − 5 kb to produce H3K27me3 domains. H3K27me3 CUT&Tag RPKM on each domain were computed. Mean and standard deviation of H3K27me3 RPKM were calculated for each region for PE and control samples, respectively. Differentially modified region were called as abs(mean(PE)-mean(control))/(std(PE) + std(control)) > 2. Enrichment of EZH2-bound regions for H3K27me3 gain or loss in PE were done with regionR::permutationTest. Overall profile of H3K27me3 on EZH2-bound regions was computed by deepTools.

### DNA methylation data processing

Raw bisulfite-converted DNA methylation sequencing data, either downloaded from NCBI SRA or directly from in-house sequencing, were processed using fastp [156] (--trim-front2 20 -w 20) (https://github.com/OpenGene/fastp) and mapped to GRCh37 + decoy reference genome using BWA-Meth (https://github.com/brentp/bwa-meth) using standard parameters. Mapped data were deduplicated and sorted using Sambamba (https://github.com/biod/sambamba) and Samblaster (https://github.com/GregoryFaust/samblaster). CpG methylation levels were extracted using Pile-O-Meth (https://github.com/dpryan79/MethylDackel) toolkit. For all libraries, conversion rate was quality-controlled by CHH methylation level > 99%. Basic statistics of in-house sequencing library were further quality-controlled by on-target rate and on-target coverage with bedtools (https://github.com/arq5x/bedtools), and duplication rate and mapping rate with Sambamba.

For mouse data of extraembryonic tissue (ExE) methylation [50], the sequencing reads were similarly preprocessed and mapped to mm9 reference. ExE-specific de novo methylation region from Smith et al. [50] were directly used. To compare methylation level on human homologous regions, these mouse methylation regions were lifted-over from mm9 to GRCh37.

For Chimpanzee data of sperm methylation, the processed CpG methylation level from Molaro et al. [157] was directly used, with lift-over from hg18 and pantro2 to GRCh37.

### Differential methylation analysis

CpG methylation level (beta: defined as reads of C nucleotide over total read coverage on a single C or G base on CpG loci) was measured for each CpG loci across the genome as mentioned above using Pile-O-Meth. For each locus, beta from preeclampsia or non-preeclampsia (including normal, gestational hypertension, and gestational diabetes) pregnant female were summarized in R (3.6.2) using an in-house script. Differentially methylated loci (DML) were defined as follows: (1) *P* < 0.01 for *t*-test between preeclampsia- and non-preeclampsia individuals; (2) beta difference between preeclampsia and non-preeclampsia individuals > 0.1.

### Differentially methylated region (DMR) analysis

Initial DMR candidate were made by merging within-100 bp-apart DML. The average beta of each initial DMR were calculated as mean beta of all CpG encompassed in the DMR. This average beta was subjected to *t*-test, and *P* < 0.01 regions were selected as candidate “seed” DMR. Segments of methylation difference level were computed using a circular binary segmentation approach on beta difference between preeclampsia and non-preeclampsia placenta with DNAcopy (https://github.com/veseshan/DNAcopy). K-means clustering was performed using R (3.6.2) on the methylation beta difference on each segment, and clusters of segments fully encompassed candidate “seed” DMR were selected as true DMR candidate (Additional file 6: Table S5).

### Pseudotime analysis of methylation data

Single-cell or bulk methylation sequencing (bisulfite sequencing: WGBS, GSE81233 [42, 158]) data and metadata were collected from NCBI SRA. In-house data were described as mentioned before. Data mapping was done with Monocle3 (https://github.com/cole-trapnell-lab/monocle3) with standard practices, with the mean beta value as “expression value.” In this and the following combined ATAC-seq analysis, we chose Monocle3 over other tools because of its simplicity in processing large number of individually sequenced scATAC/scWGBS libraries and to combine them together with bulk sequencing data. Dispersion was estimated with negative binomial model. Mean beta values from PE-hypermethylated LTR12C regions and PE-hypomethylated de novo methylated loci in extraembryonic tissue are used for clustering and pseudotime analysis. Data were preprocessed using 10 dimensions with UMAP. To cluster samples from different techniques, we used “align_cds” function from Monocle3 using mutual nearest neighbour alignment method [159] and performed pseudotime trajectory analysis on these aligned data.

### Pseudotime analysis of ATAC-seq data

Single-cell or bulk ATAC sequencing data (SRP163205 [45] and GSE101571 [76]) and metadata were collected from NCBI SRA. In-house data were described as mentioned before. The overall processing was similar to pseudotime analysis of methylation data mentioned above, with the only difference that ATAC-seq RPKM from all DMR regions were used as “expression level” in the analysis.

### Overlapping region analysis

DMR and ATAC-seq peak regions were overlapped with known ultraconserved noncoding elements (UCNE) [79], human-accelerated regions (HAR) [78], and placental animal-specific accelerated regions (PAR) [77] and statistical significance of set enrichment were calculated with Fisher’s exact test in R (3.6.2). For DMR region overlapping with known imprinted genes, imprinted gene list is downloaded from https://www.geneimprint.com/ and only experimentally validated imprinted genes were used. Permutation-based overlap size statistics is performed by regioneR (v1.28.0). Statistics of DMR region overlapping with all known chromVAR [80] region annotation sets is performed by LOLA (v1.26.0).

### Sequencing of a paternally derived PEG10 allele

The paternally derived PEG10 allele chr7:94665871 T > A was initially discovered by comparing the single-cell RNA-seq data on fetal and maternal faces of placenta of a PE female, 2005139. Presence of the allele in placental trophoblast was confirmed by scATAC SNV analysis. However, the maternal cells have no scATAC reads covering on this region, probably because of no expression of PEG10 in the maternal cells. Oral swabs of the mother (2005139), father (2105466), and progeny (2105342, after birth) were taken and Sanger-sequenced using primers 5′CACATCCTCTCTGAAACGGCT3′ and 5′CCTTTCCACACTGCACCGAT3′. Primer validity was confirmed with in-house known heterozygous carrier and homozygous wild-type controls of the same allele. Low-pass WGS (LWGS) with standard double-stranded library preparation assay using in-house adaptors were also used to confirm haplotype transmission of the T allele in progeny was from mother. To do this, LWGS data were mapped using bwa-mem and SNV were analyzed using sentieon DNAscope. Haplotype analysis was done with Eagle [160].

### RNA velocity in arterial and trophoblast cells

RNA dynamical velocity analysis of single-cell 3′ RNA sequencing data, including inferring dynamical streamline, computing dynamic genes, latent time, and the probability of root cell and terminal cell, was performed with scVelo [66] using Reticulate [150] in R (3.6.2) with Python 3 and following standard protocol. PE and control trophoblast were performed RNA velocity separately, while PE and control arterial cells were performed as a whole.

### Association of PE transcriptome with single-cell RNA profiles by Scissor

Raw data of bulk placenta RNA-seq was downloaded from GSE148241 [63, 64] and mapped by STAR [161] to give read count matrix covering genes. The parameters used in mapping and quantification were as follows: alignment was performed with STAR (version 2.5.3a). STAR reference genome was built with '--runMode genomeGenerate --runThreadN 16 --genomeDir b37/STAR-genome --genomeFastaFiles b37/human_g1k_v37_decoy.fasta --sjdbGTFfile b37/Homo_sapiens.GRCh37.82.gtf --sjdbOverhang 100'. Alignment was performed with '--chimSegmentMin 20 --chimScoreMin 5 --quanMode GeneCounts --twopassMode Basic --outSAMtype BAM SortedByCoordinate'. The quantified gene table was then passed to DEseq2 [162] and analyzed with the default parameter, only filtering for genes that are expressed. Expressed genes were filtered as 'count >  = 10'. Differentially expressed genes were defined as the pvalue (raw *P*-value) < 0.05 and absolute value of log2FoldChange (original log2 fold change) > 0.5.

The correlation between bulk PE placental transcriptome and scRNA placental cell was inferred by Scissor [62] (https://sunduanchen.github.io/Scissor/vignettes/Scissor_Tutorial.html), setting alpha = 0.05, family = “binomial”, cutoff = 0.2 (the default setting, Scissor selected cells should not exceed 20%). To avoid technical aberration caused by the imbalance of single-cell number in each cell type, scRNA placental cells were down-sampled to 1000 cells per cell type. The Scissor-positive cell is defined as the average of correlations with all bulk samples is greater than 0 and the number of positive correlations is larger than the number of negative correlations, and the Scissor-negative cell is defined as the average of correlations with all bulk samples is less than 0 and the number of negative correlations is larger than the number of positive correlations. By Scissor::reliability.test, the *p*-value of Scissor identified association is 0.000 and AUC is 0.8738095.

### Association of PE GWAS risk loci with single-cell chromatin accessibility profiles by SCAVENGE

For GWAS risk loci association, PE-associated GWAS loci from [102] were downloaded from GWAS catalog. The raw genotyping dataset is not publicly available and fine-mapping of risk loci is impossible. To take an approximation of posterior probability of association, we then took the log of adjusted *P*-value of these significantly associated loci and normalized them to a max of 1. A peak-x-cell matrix is extracted from the full scATAC dataset from ArchR and subjected to LSI, MNN analysis in SCAVENGE [65]. *Z*-scores of loci enrichment in single cells, and cell–cell similarity-based propagation of the *Z*-score, were performed with SCAVENGE. The seed cells were selected as the top 0.1% *Z*-score enriched cells. Cells with top 25% TRS were considered positively associated with trait.

### Immunostaining

Placenta tissues (from 3 SPE, 3 control, and 1 SPE with sIUGR donors) were processed with standard paraffin section protocol to 10–30-µm-thick slides. Immunostaining with primary antibodies, including Anti-PEG10 (1:1500, Abcam, ab215035), Anti-CK7 (1:1500, Abcam, ab9021) and Anti-CD31 (1:1000, Abcam, ab9498), and DAPI followed the instruction of Opal 7 color manual Kit (AKOYA Biosciences, NEL811001KT). Fluorescent slides were scanned using the PhenoImager (Akoya Biosciences) using × 20 objective with the following exposure times: DAPI MSI, 0.38 ms; Cy5 MSI (PEG10), 17.33 ms; Cy3 (CD31), 15.76 ms; FITC (CK7), 6.58 ms. Images were generated using inForm software (Akoya Biosciences).

### Automatic quantification of fluorescent staining intensity

Image exportation was done by inForm Tissue Finder software (version: 2.6). After exportation, the images were imported to Fiji to split into different channels. Channel-specific images were imported into R (version: 4.1.1). Masking was performed to get endothelial and trophoblast cells, by identifying the contour with CD31 or CK7 staining. Quantification of PEG10 intensity inside of cell contours was subsequently performed. Masking and quantification were performed with computeFeatures.basic function from EBImage (package version 4.34.0).

### Gene set enrichment analysis

Differentially expressed gene set between PE and control arterial cells were calculated by Seurat::FindAllMarkers, setting min.diff.pct = 0, logfc.threshold = 0, only.pos = F. Gene set enrichment analysis was performed with fgsea [163] R package (version:1.18.0), with differentially expressed genes in PE and msigdbr [164] database (verrsion: 7.5.1, category = "H" or "C2"). Significant pathway is defined as pval (raw *P*-value) < 0.05 and the absolute value of NES > 1.

### Similarity between AVM and PE arterial endothelial cells

The brain vessel single-cell RNA-seq dataset was downloaded from NCBI GEO (GSE187875 [89, 100]) and used with its original cell type and pathology annotation. Seurat (v4.0) were used to extract differentially expressed genes (DEG) specifically upregulated in AVM or control arterial endothelial cells. Seurat::AddModuleScore was used to calculate the gene set activity in fetal and maternal endothelial cells of placenta.

### Correlation of cargo expression profile (Additional file 1: Fig. S29)

scRNA-upregulated, scATAC-silent genes were firstly selected as mentioned in “Definition of VLP cargo genes.” The mean expression levels of these genes in each cell type were extracted for PE and control samples, respectively. For each type of sample, Pearson’s correlation was performed between all cell types.

### Mitosis aging rate determination using EpiTrace

Mitosis age of single cells in the scATAC dataset were inferred by *EpiTrace *[69] (https://github.com/MagpiePKU/EpiTrace) following the standard protocol. Briefly, chromatin accessibility on age-associated DML sites were measured for each cell and the resulting score is subjected to iterative HMM smoothing-approximation. The deduced cell mitotic age is then divided by gestation week of the sample, to give a single-cell mitotic aging rate which approximates cell proliferation rate. Differences between mitotic aging rate were measured by Wilcox test.

### Supplementary Information

Additional file 1: Fig. S1. Placental cell type composition in this study. Fig. S2. Placental cell types with mixed-origin. Fig. S3. Similarity between accessibility and gene expression in scATAC. Fig. S4. Scissor inferred phenotype associated cells. Fig. S5. Examples of marker gene expression in PE and control placenta bulk RNAseq dataset [172–179]. Fig. S6. Cell cycle scores in trophoblast clusters. Fig. S7. Expression of trophoblast cluster marker genes. Fig. S8. The frequency of trophoblast cluster and cell cycle phase. Fig. S9. Latent time distribution across gestational week in control and PE trophoblasts. Fig. S10. Latent time of trophoblasts, grouped by trimester. Fig. S11. scRNA analysis of trophoblast cells in external validation dataset. Fig. S12. Developmental trajectory switch in PE trophoblast. Fig. S13. RNA expression and transcription factor binding activity of master transcription factors (TF). Fig. S14. Transcription factor activities upstream of EZH2 in PE placenta. Fig. S15. Master transcription factor controlled velocity gene set expression in the trophoblast cells in external validation dataset. Fig. S16. Bulk ATAC seq assay on frozen placenta tissue. Fig. S17. Differential DNA methylation between control and PE. Fig. S18. Differential methylation in fetal and maternal face of placenta between PE and control. Fig. S19. Deficient ExE-specific *de novo *methylation in paternally imprinted loci in PE placenta. Fig. S20. DNA methylation levels on recently evolved, primate-specific retrotransposons, particularly the imprinted LTR12C, discriminate PE and control placenta. Fig. S21. PE DMR regions are enriched with PRC2 related binding loci. Fig. S22. Differential expression of imprinted genes in trophoblast. Fig. S23. Reduced H3K27me3 modification on EZH2-controlled genes in PE placenta. Fig. S24. Reduced H3K27me3 modification on paternally imprinted genes in PE placenta. Fig. S25. PE trophoblast overexpressed genes to stall its cell cycle progression. Fig. S26. Differential gene expression in each trophoblast lineage. Fig. S27. Differential epigenetic modification around *PEG10*. Fig. S28. Motif similarity of cargo genes. Fig. S29. Heatmap of similarity of cargo gene RNA expression between trophoblasts and endothelial cells. Fig. S30. Differential expression and cell cycle activity in endothelial cells. Fig. S31. Differential expressed genes in Wnt beta Catenin and TGF-beta pathways. Fig. S32. PEG10 qPCR and western blot. Fig. S33 Transcriptional dysregulations under PEG10 perturbation.

Additional file 2: Table S1. Characterization of donors in the study.

Additional file 3: Table S2. Single cell annotation.

Additional file 4: Table S3. Statistical details for trophoblast CytoTRACE analysis.

Additional file 5: Table S4. Differential peaks in bulk ATAC assay.

Additional file 6: Table S5. PE specific differential methylation regions.

Additional file 7: Table S6. Differentially expressed genes in trophoblast.

Additional file 8: Table S7. List of cargo genes.

Additional file 9. Table S8. QC metrics for sequencing assays in the study.

Additional file 10. Uncropped images of Western blots in Fig. S32.

Additional file 11. Protocol for accessing the data with controlled access on GSA.

Additional file 12. Review history.

## Data Availability

The raw sequence data reported in this paper have been deposited in the Genome Sequence Archive (Genomics, Proteomics & Bioinformatics 2021) in National Genomics Data Center (Nucleic Acids Res 2022), China National Center for Bioinformation / Beijing Institute of Genomics, Chinese Academy of Sciences (accession number: HRA001423 [165]) that are publicly accessible at https://ngdc.cncb.ac.cn/gsa-human. The dataset has been reported to the China Human Genetic Resources Management Office (CHGR), Ministry of Science and Technology of The People’s Republic of China. Data access is under regulation of the Chinese Biosafety Law and CHGR policy. Researchers wish to access the data should apply on the GSA website following the instructions provided on Additional file 11. The data access committee (DAC), Drs. Yuan Wei (weiyuanbysy@163.com) and Yi Zhang (zy@eulertechnology.com), would evaluate the request and help the researcher to access the data. The DAC would reply by email within a week of the request. The source codes for reproducing the figures were deposited under GPL 3.0 license at GitHub [166]. The source data accompanying the source codes were deposited under CC 4.0 license at Zenodo [167]. The study additionally used a series of public datasets from NCBI SRA sequencing read archive (https://www.ncbi.nlm.nih.gov/sra) and the Chinese Genome Sequencing Archive (https://bigd.big.ac.cn/) including H3K27ac ChIP-seq data of placenta from Roadmap Project: GSM1127147 [168, 169]; human embryo ATAC-seq: SRP163205 [45, 170] and GSE101571 [76, 171]; human embryo methylation sequencing: GSE81233 [42, 158]; human placenta single-cell RNA-seq: PRJNA492324 [54, 56], PRJEB28266 [55, 57], GSE150578 [58, 59], and GSE173193 [70]; human placenta bulk RNA-seq GSE148241 [63, 64]; human brain vascular single cells RNA-seq GSE187875 [89, 100]. The Encode transcription factor binding sites, ChromHMM tracks, CpG islands, repeat regions data, and lift-over chains were collected from UCSC Genome Browser (http://www.genome.ucsc.edu/). Chromatin interactions were downloaded from 4Dgenome database (http://4dgenome.int-med.uiowa.edu). Transcription factor binding information for bulk ATAC-seq analysis were downloaded from ReMap database (http://pedagogix-tagc.univ-mrs.fr/remap).
