# Supplementary material for: Disruption of maternal vascular remodeling by a fetal endoretrovirus-derived gene in preeclampsia
Source: Genome Biol. 2024 May 7;25:117. doi: 10.1186/s13059-024-03265-z (PMC11075363; doi:10.1186/s13059-024-03265-z)
Supplement: Supplementary file 1 — Additional file 1: Fig. S1. Placental cell type composition in this study. Fig. S2. Placental cell types with mixed-origin. Fig. S3. Similarity between accessibility and gene expression in scATAC. Fig. S4. Scissor inferred phenotype associated cells. Fig. S5. Examples of marker gene expression in PE and control placenta bulk RNAseq dataset [172–179]. Fig. S6. Cell cycle scores in trophoblast clusters. Fig. S7. Expression of trophoblast cluster marker genes. Fig. S8. The frequency of trophoblast cluster and cell cycle phase. Fig. S9. Latent time distribution across gestational week in control and PE trophoblasts. Fig. S10. Latent time of trophoblasts, grouped by trimester. Fig. S11. scRNA analysis of trophoblast cells in external validation dataset. Fig. S12. Developmental trajectory switch in PE trophoblast. Fig. S13. RNA expression and transcription factor binding activity of master transcription factors (TF). Fig. S14. Transcription factor activities upstream of EZH2 in PE placenta. Fig. S15. Master transcription factor controlled velocity gene set expression in the trophoblast cells in external validation dataset. Fig. S16. Bulk ATAC seq assay on frozen placenta tissue. Fig. S17. Differential DNA methylation between control and PE. Fig. S18. Differential methylation in fetal and maternal face of placenta between PE and control. Fig. S19. Deficient ExE-specific de novo methylation in paternally imprinted loci in PE placenta. Fig. S20. DNA methylation levels on recently evolved, primate-specific retrotransposons, particularly the imprinted LTR12C, discriminate PE and control placenta. Fig. S21. PE DMR regions are enriched with PRC2 related binding loci. Fig. S22. Differential expression of imprinted genes in trophoblast. Fig. S23. Reduced H3K27me3 modification on EZH2-controlled genes in PE placenta. Fig. S24. Reduced H3K27me3 modification on paternally imprinted genes in PE placenta. Fig. S25. PE trophoblast overexpressed genes to stall its cell cycle progression. [file 13059_2024_3265_MOESM1_ESM.docx]

**Supplementary Figures and Legends**

**Figures S1 – S33.**

**
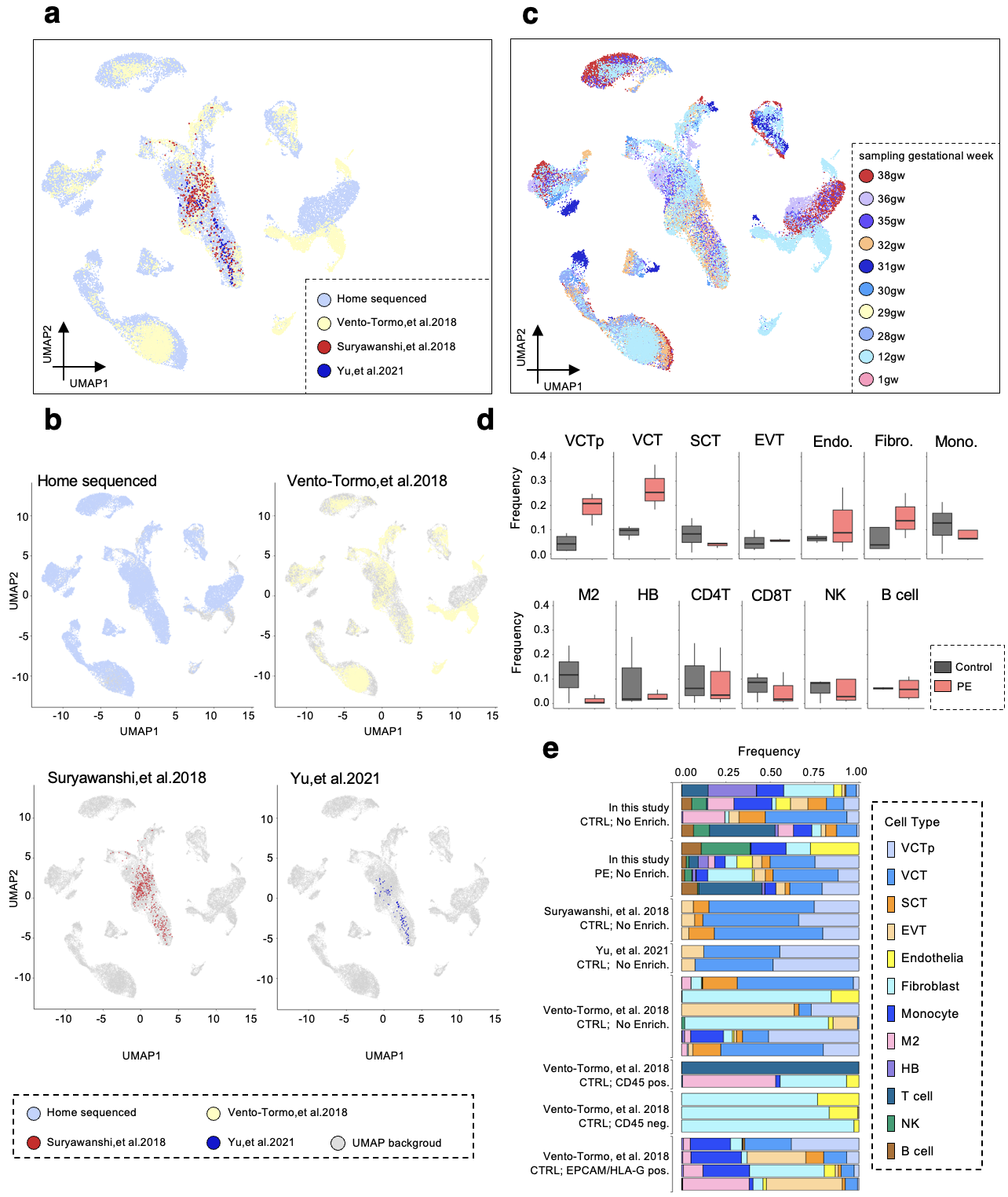
**

**Fig. S1. Placental cell type composition in this study.**

**(a)** UMAP projection of data sources in scRNA. **(b)** UMAP projection of each data source in **(a)**. **(c)** UMAP projection of gestational week in scRNA. **(d)** Box-and-whisker plot of cell type frequency comparing PE (dark grey, N = 4) with in-house sequenced control (red, N = 4) placenta samples. Prevalence of VCTp, VCT, fibroblasts and endothelial cells are higher in PE placenta, and prevalence of SCT, M2, and HB cells are lower in PE placenta. **(e)** Cell type composition of donors in this study. Donors can be classified by the source of sample collection (placenta samples collected in this study, labelled as ‘in this study’; data from PRJNA492324 dataset [54, 56], labelled as ‘Suryawanshi, *et al*. 2018’; data from PRJEB28266 dataset [55, 57], labelled as ‘Vento-Tormo, *et al*. 2018’; data from GSE150578 dataset [58, 59], labelled as ‘Yu, *et al*. 2021’), clinical phenotype (non-preeclampsia pregnancy, control and preeclampsia pregnancy, PE) and enrichment method before single cell RNA sequencing (no enrichment; enriching CD45 positive cells, labelled as ‘CD45.pos’; enriching CD45 negative cells, labelled as ‘CD45.neg’; enriching EPCAM or HLA-G positive cells, labelled as ‘EPCAM/HLA-G pos’).


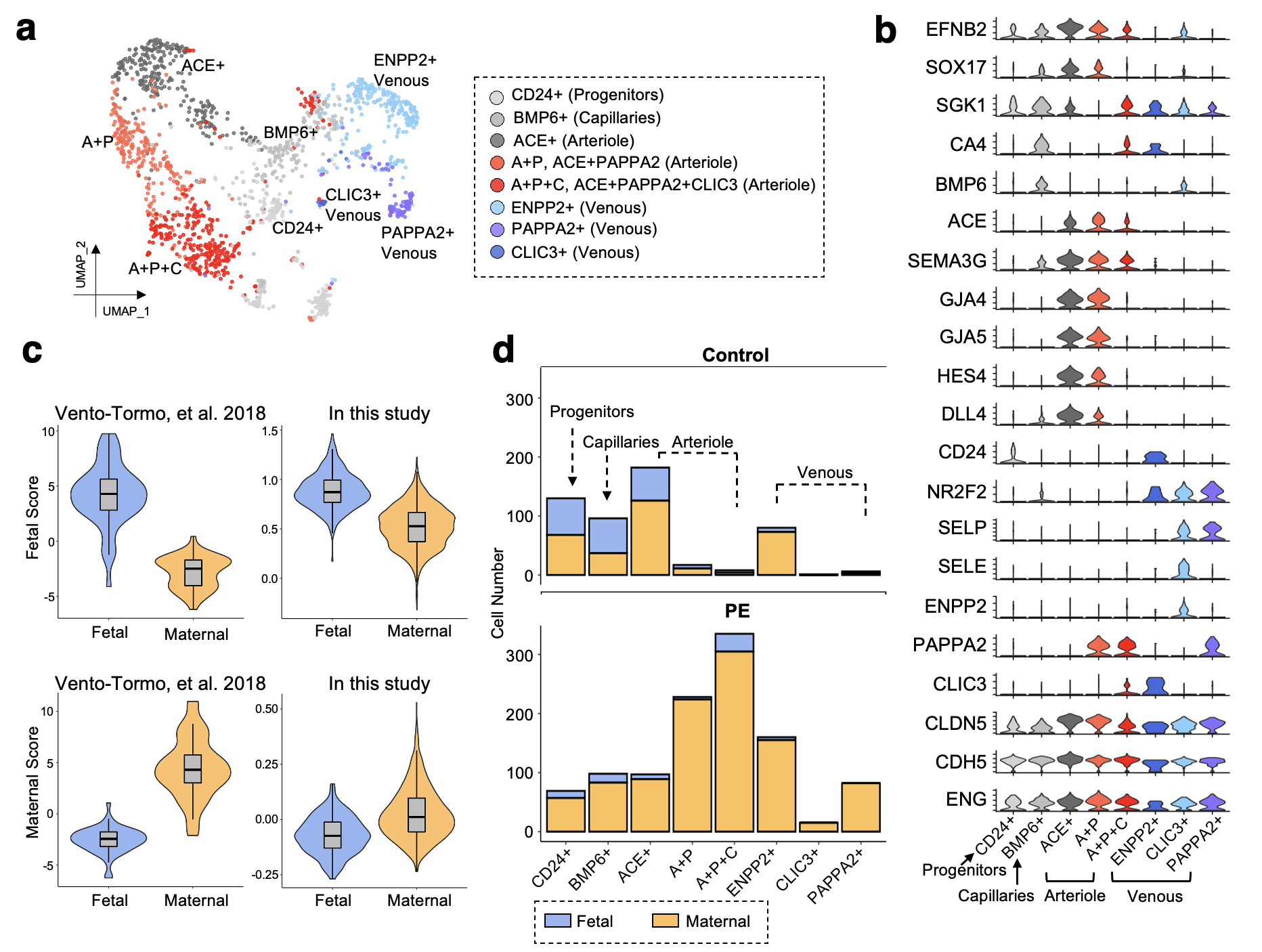


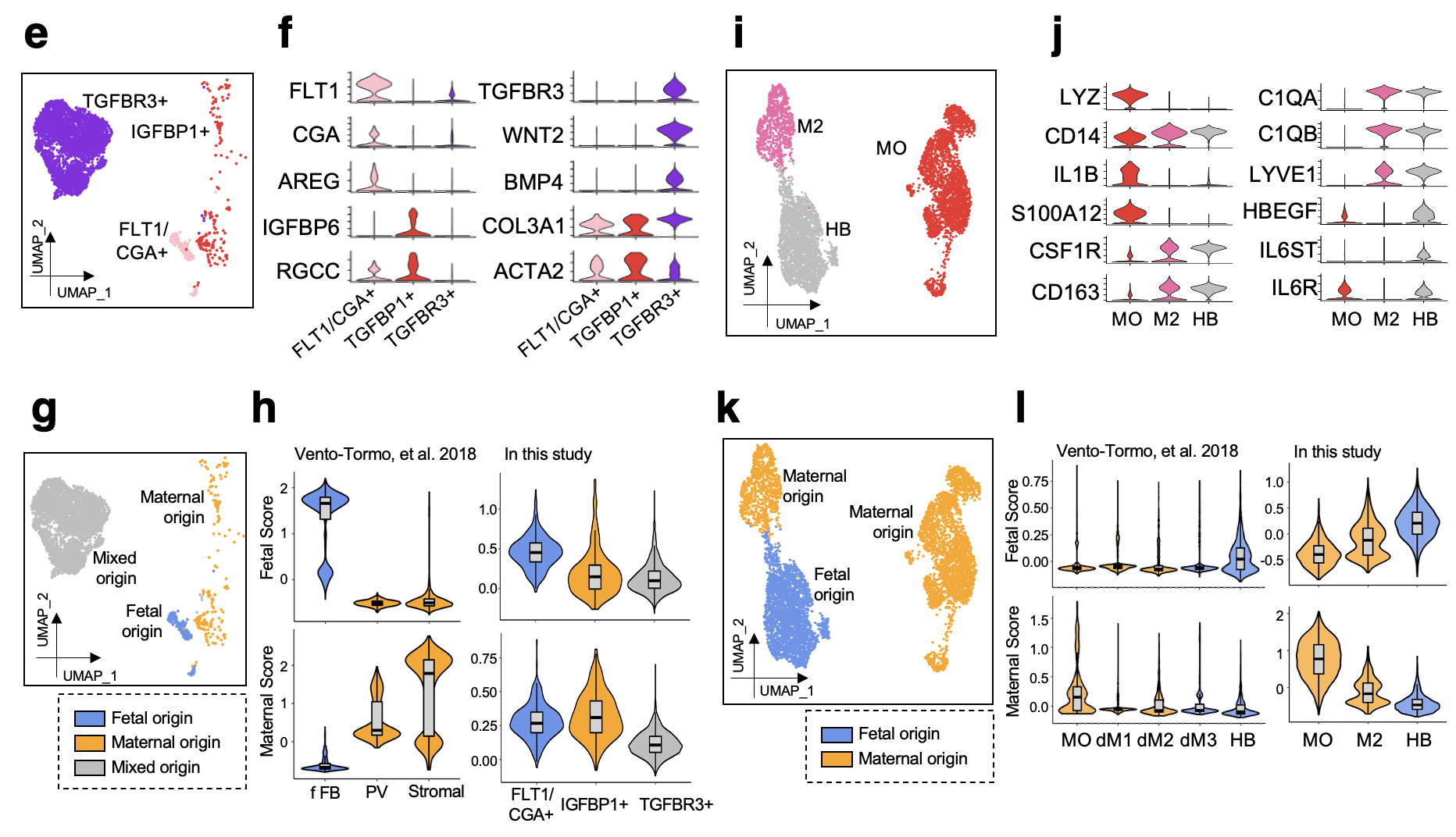


**Fig. S2. Placental cell types with mixed-origin.**

**(a)** UMAP projection of scRNA endothelial cells. **(b)** Endothelial marker gene expression in clusters of **(a)**. Color code of endothelial clusters is matched with **(a)**. **(c)** Fetal- or Maternal-gene set expression of maternal and fetal endothelia (Methods) in scRNA dataset from Vento-Tormo, *et al*. 2018 [58, 59] (left) or endothelial cells in this study (right). **(d)** Cell number of maternal (orange) or fetal (blue) origins of cells in each cluster between control and PE. **(e)** UMAP projection of scRNA fibroblast cells. **(f)** Fibroblast marker gene expression in clusters of **(e)**. Color code of fibroblast clusters is matched with **(e)**. **(g)** UMAP projection of cell origin in scRNA fibroblast. **(h)** Fetal- or Maternal-gene set expression of maternal and fetal stormal/fibroblast/perivascular cells (Methods) in scRNA dataset from Vento-Tormo, *et al*. 2018 [58, 59] (left) or fibroblast cells in this study (right). **(i)** UMAP projection of scRNA meyloid cells. **(j)** Myeloid marker gene expression in clusters of **(i)**. Color code of myeloid clusters is matched with **(i)**. **(k)** UMAP projection of cell origin in scRNA myeloid. **(l)** Fetal- or Maternal-gene set expression of maternal and fetal myeloid (Methods) in scRNA dataset from Vento-Tormo, *et al*. 2018 [58, 59] (left) or myeloid in this study (right).


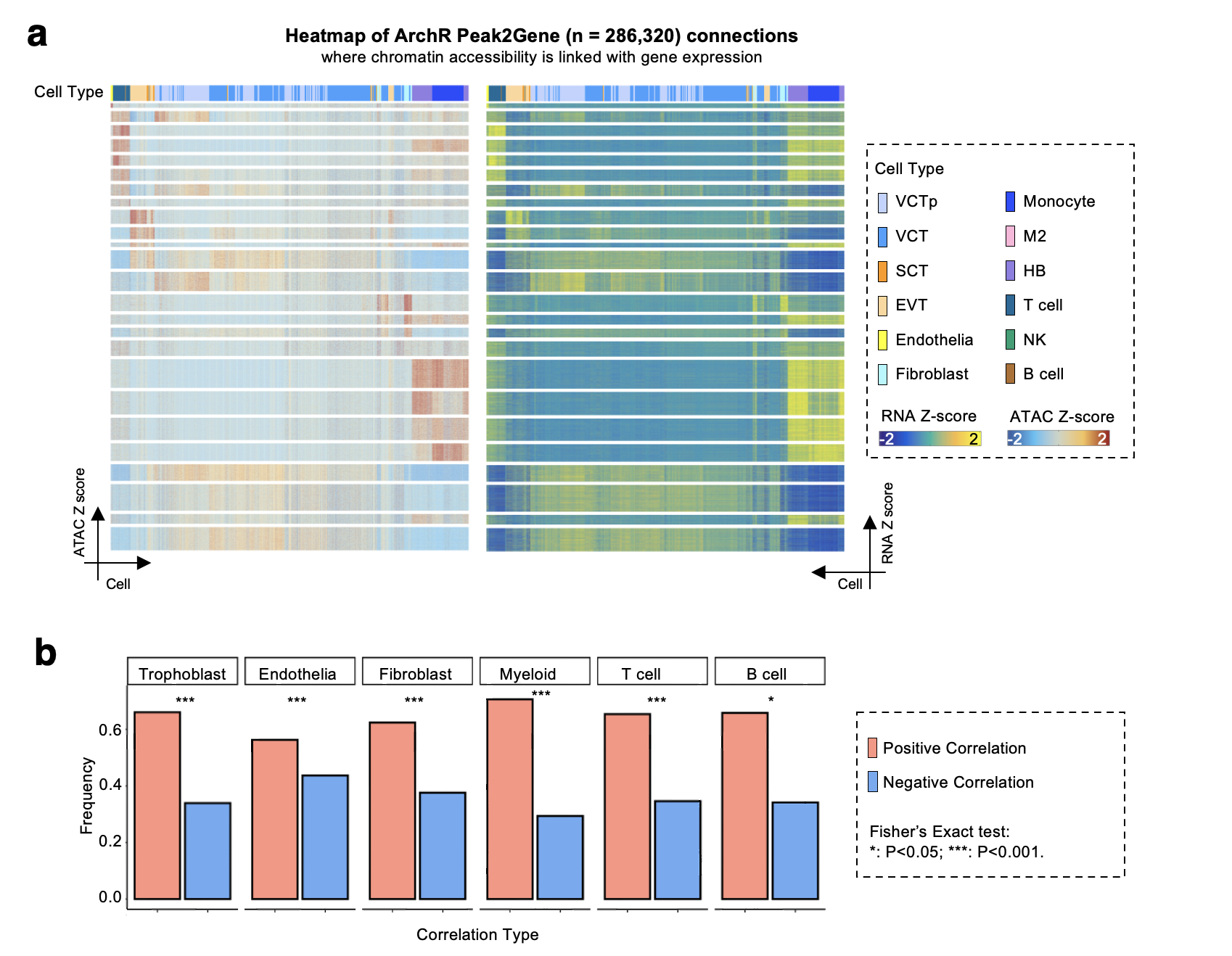


**Fig. S3. Similarity between accessibility and gene expression in scATAC.**

**(a)** Heatmap of similarity between chromatin accessibility (left) and RNA expression (right) in integrated scATAC data in Fig. 1e. **(b)** Statistic of peak-to-gene link in **(a)**. Positive correlation (Pos, red) between chromatin accessibility and gene expression was defined as [ATAC Z score * RNA Z score >0]; negative correlation (Neg, blue) between chromatin accessibility and gene expression was defined as [ATAC Z score * RNA Z score < 0]. Fisher's exact test was performed. Positive correlation peak-to-gene link is significantly enriched in each type of placental cell. P-values: Fisher's exact test by counts. *: P<0.05; ***: P<0.001.


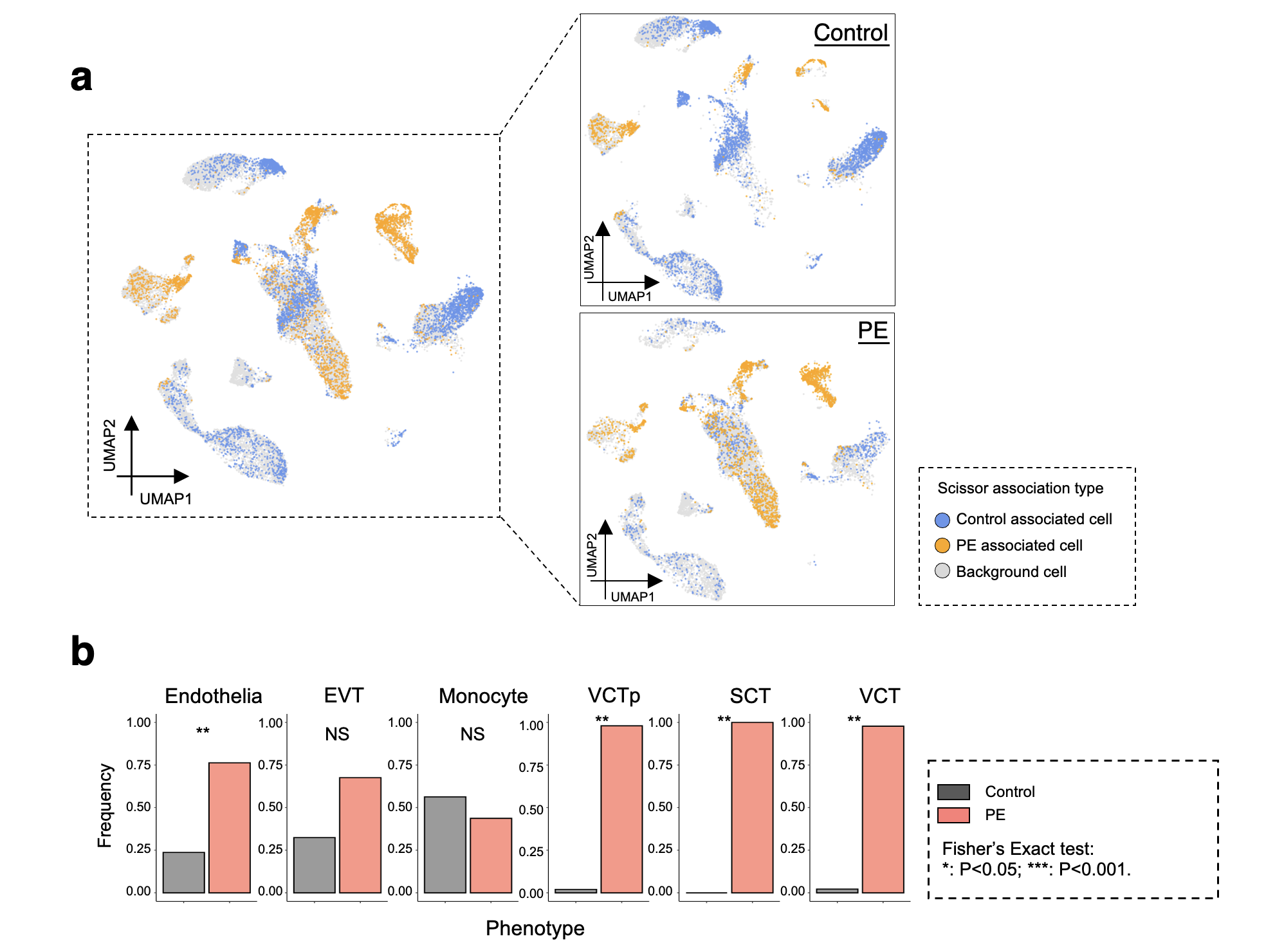


**Fig. S4. Scissor inferred phenotype associated cells.**

**(a)** Left: UMAP projection of phenotype associated scRNA cells in Fig. 1b and 1f. Right: UMAP projection of phenotype associated scRNA cells in control (top) and PE (bottom) placentas. Bule: control-associated cells; orange: PE-associated cells; grey: background cells. **(b)** Frequency of PE-associated cells in trophoblast, endothelial cells and monocytes. Grey: control placentas; red: PE placentas. Statistically significant pairs of comparisons were shown under the plot. P-values were tested by Fisher's exact test, NS: not significant, *: P<0.05, **: P<0.01.


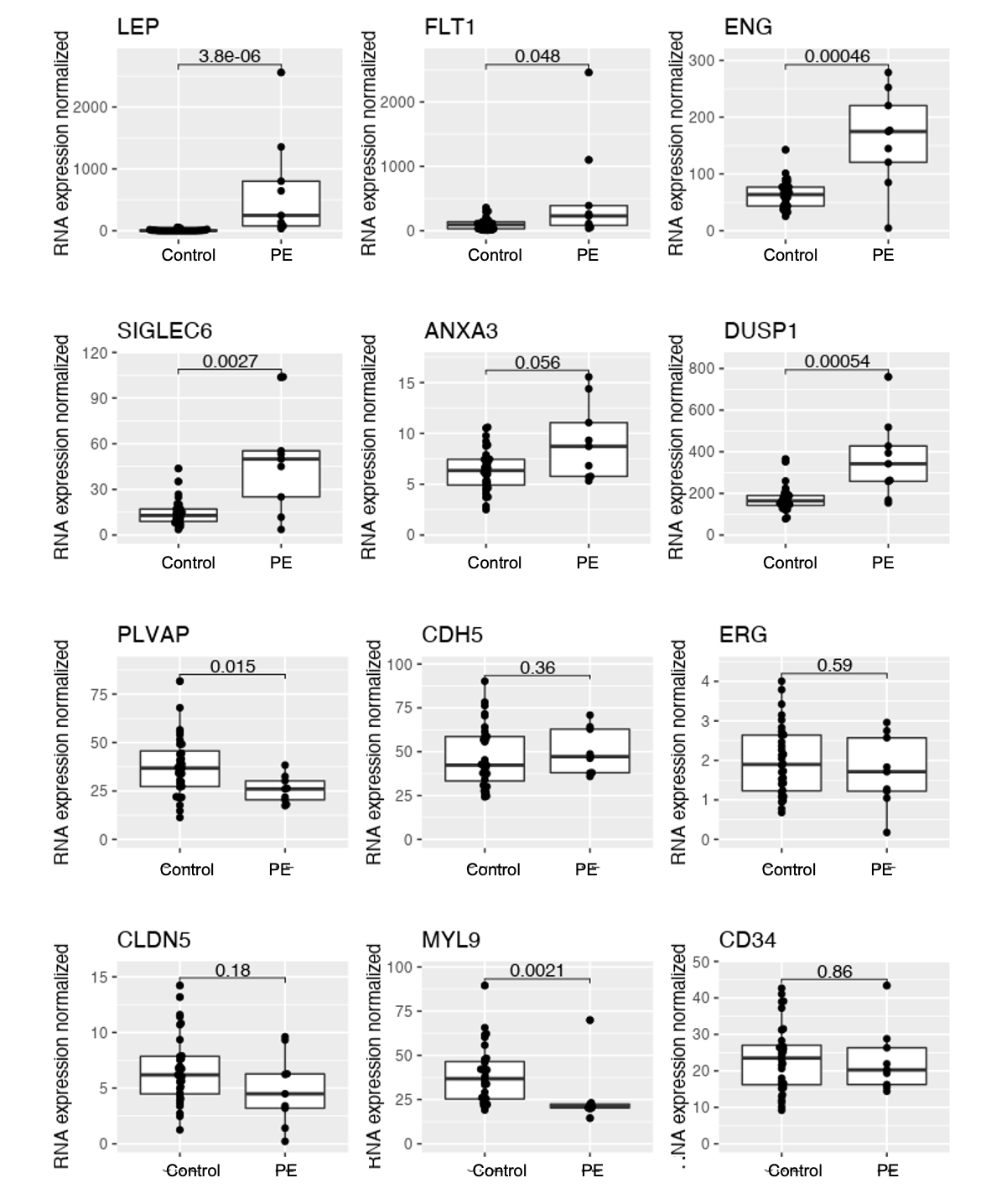


**Fig. S5. Examples of marker gene expression in PE and control placenta bulk RNAseq dataset.**

Normalized RNA expression of reported PE upregulated genes, including *LEP [63]*, *FLT1 [22, 172, 173]*, *ENG [21, 24]*, *SIGLEC6 [174]*; trophoblast upregulated genes, such as *ANXA3* [175] and *DUSP1* [176] between placentas from normal pregnancy (control) and early-onset of severe preeclampsia (PE) in bulk RNAseq dataset, GSE148241 [63, 64]. In addition, endothelial markers including, *PLVAP*, *CDH5*, *CLDN5 [177]*, *ERG [178]*, fibroblast marker, *MYL9* [179] and hematopoietic stem cell marker *CD34 [180]*. Gene expression result indicates that the PE and control placenta bulk RNAseq dataset (GSE148241 [63, 64]) is consist of both fetal cells (such as, trophoblast, fetal endothelial, fetal fibroblast and fetal hematopoietic lineage cells) and maternal cells (maternal endothelia, maternal fibroblast and maternal hematopoietic lineage cells).


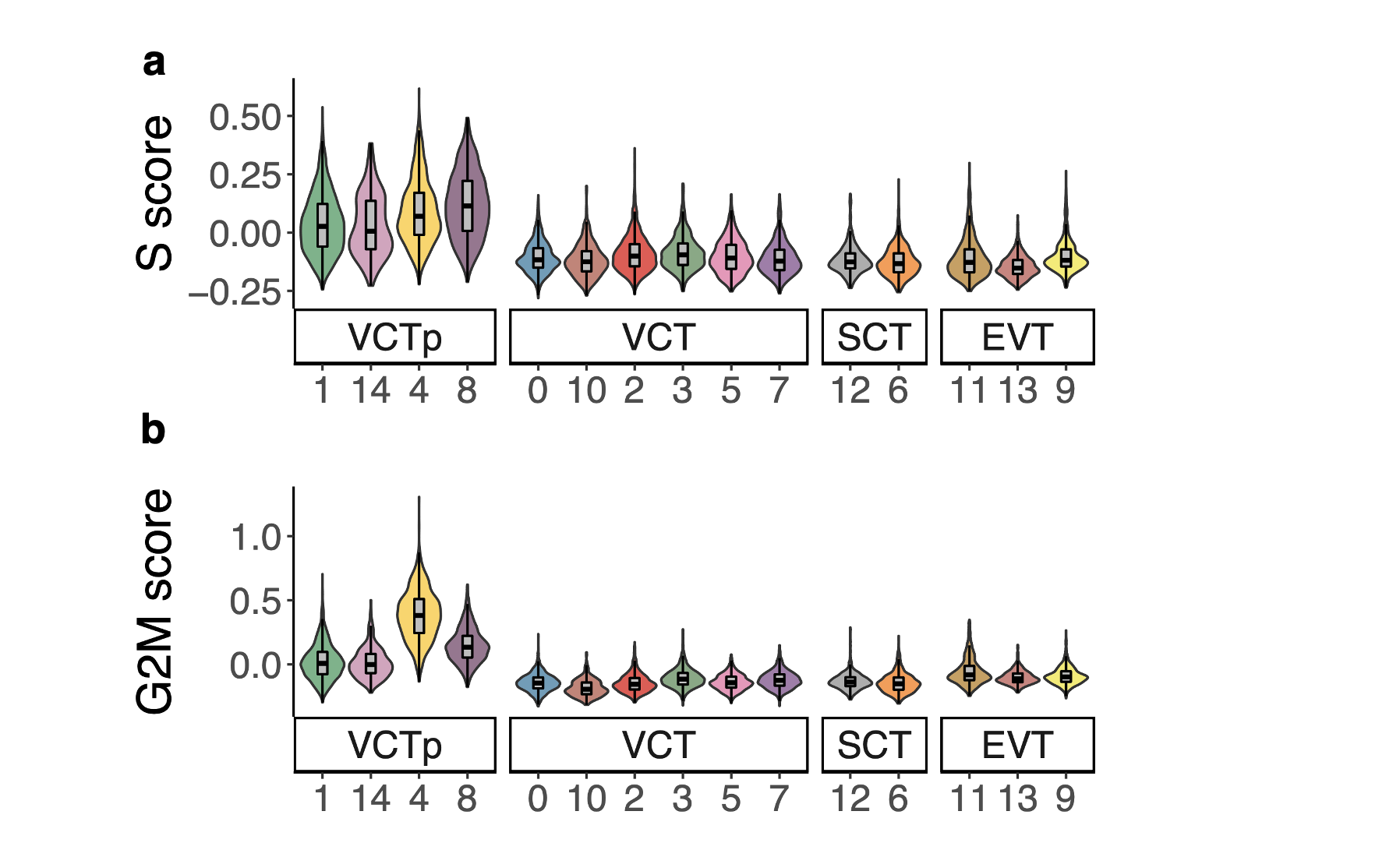


**Fig. S6. Cell cycle scores in trophoblast clusters.**

**(a)** S phase gene set activity (S score) in each trophoblast cluster in Fig.2a. **(b)** G2/M phase gene set activity (G2M score) in each trophoblast cluster in Fig.2a.


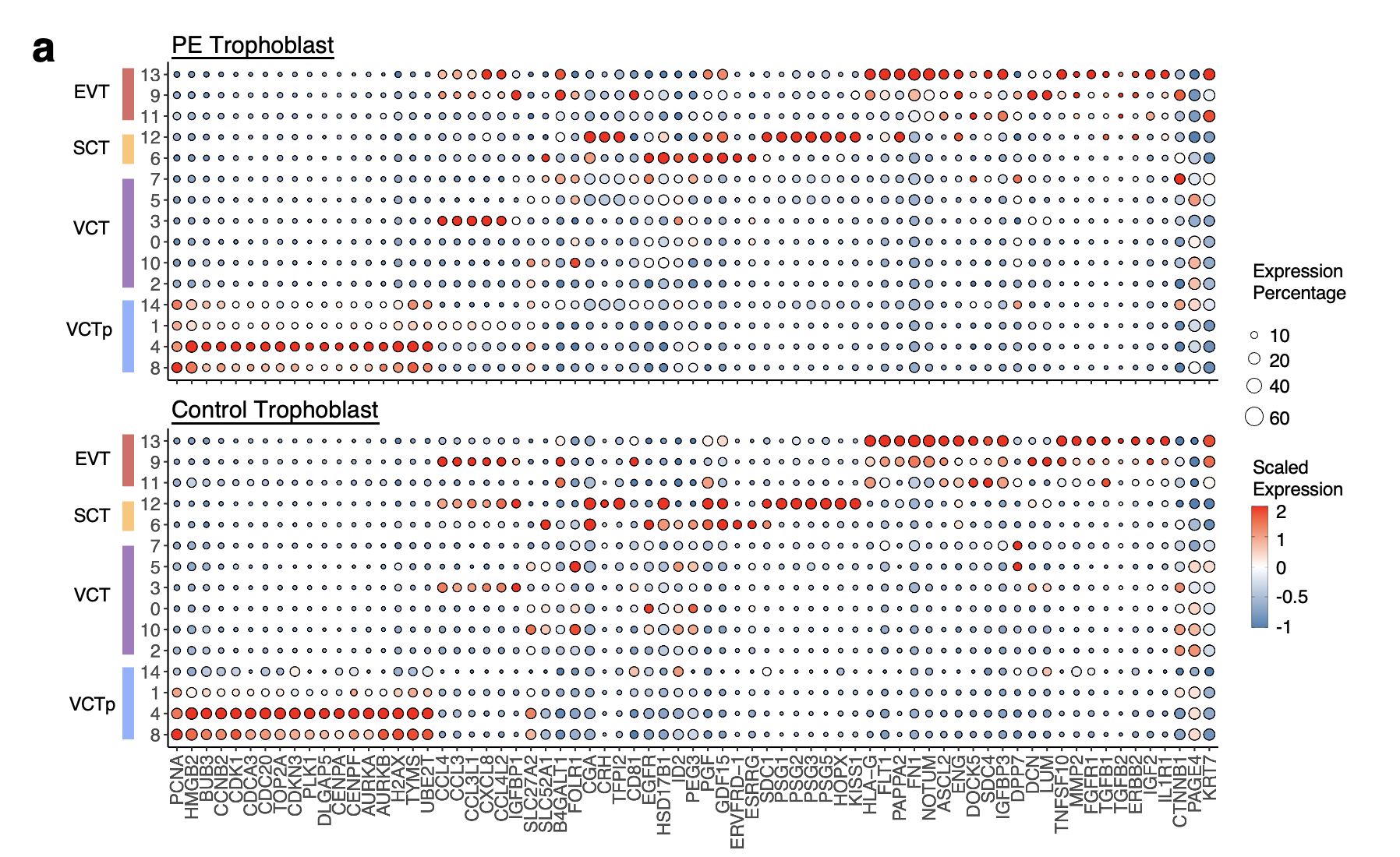


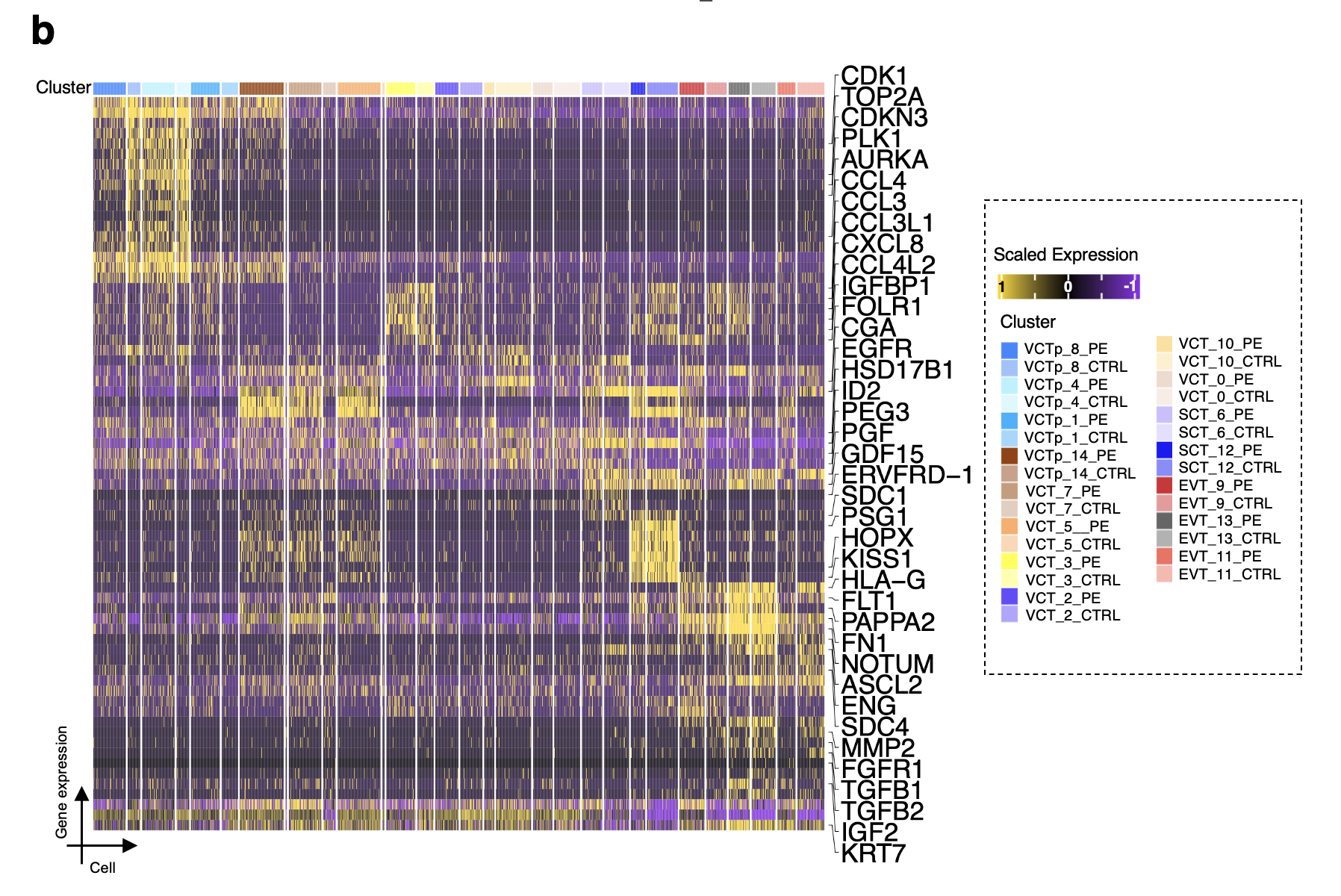


**Fig. S7. Expression of trophoblast cluster marker genes.**

**(a)** Expression of trophoblast cluster marker genes in control (bottom panel) and PE (top panel). All trophoblast clusters express high level of *PAGE4/KRT7/CTNNB1*. VCTp clusters (clusters: 1/4/8/14) express high level of cell proliferating genes, *HMGB2/CCNB2/CDK1/TOP2A/AURKA/AURKB/CDKN3/PLK1*. VCT clusters (cluster: 0/2/3/5/7/10) express high level of nutrition transportation and sensing genes, *SLC27A2/ SLC52A1/ FOLR1*. SCT clusters (cluster: 6/12) are characterized by the expression of *CGA/GDF15/SDC1/PGF/HSD17B1*. EVT clusters (clusters:9/11/13) are marked by the expression of *HLA-G/PAPPA2*. **(b)** Heatmap of the single cell expression of trophoblast cluster marker genes in control and PE. The color of top annotation bar indicates the trophoblast clusters in Fig. 2a. The transparency of top annotation bar indicates clinical phenotype: control trophoblast is solid while PE is transparent. Expression levels are scaled by Z (-1~1) and colored from purple to yellow.


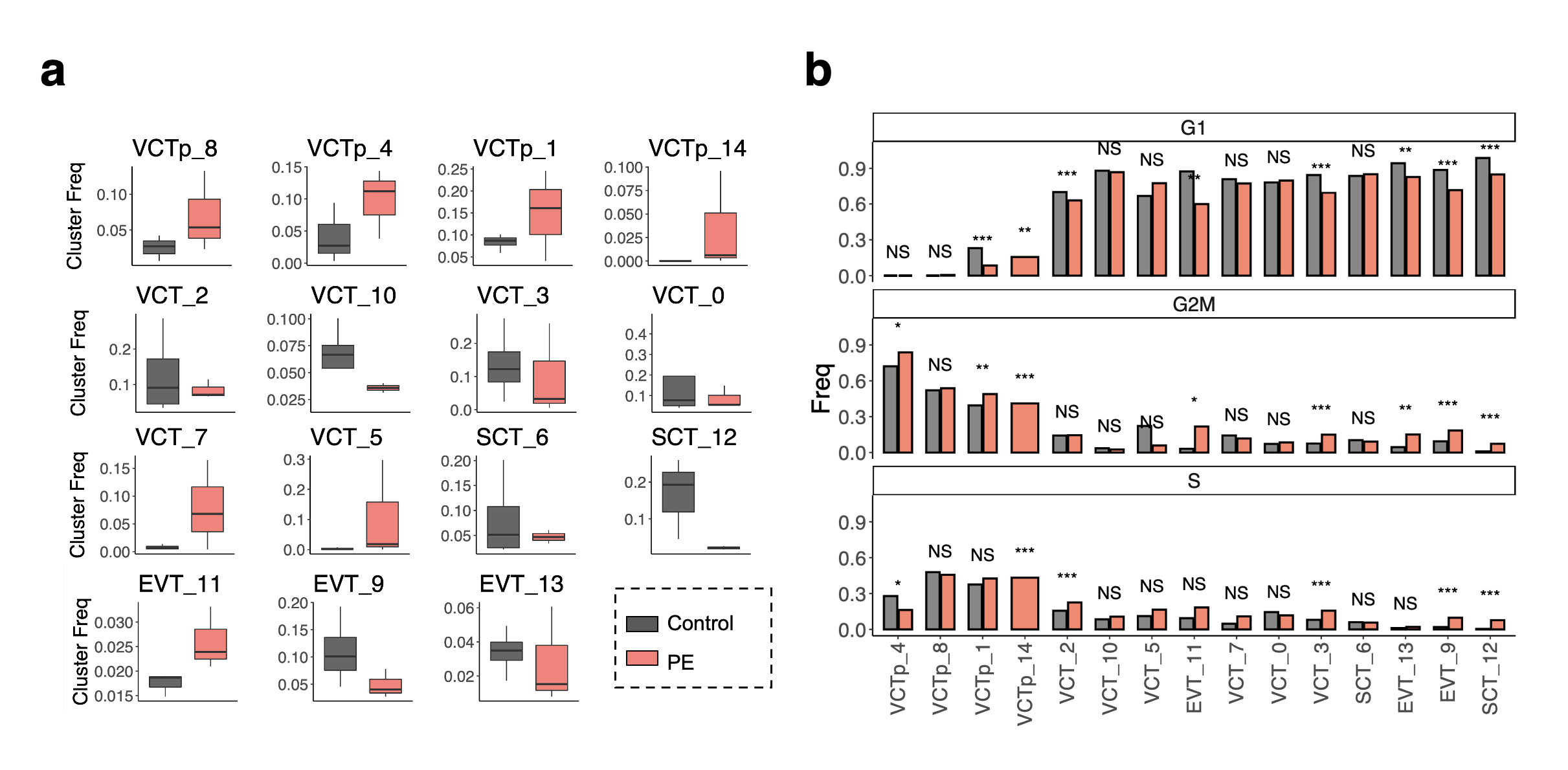


**Fig. S8. The frequency of trophoblast cluster and cell cycle phase.**

**(a)** Cell number percentage (Cluster Freq) of each trophoblast cluster in Fig. 2a between PE (red) and control (grey). **(b)** The frequency of inferred cell cycle phase in trophoblast clusters in Fig. 2a. P value was calculated by Fisher's exact test between control and PE of each phase per cluster.

**Fig. S9. Latent time distribution across gestational week in control and PE trophoblasts**

**(a)** Latent time distribution of Day21 iTSC (GSE150578 [58, 59]), 1st Trimester vill (PRJNA492324 [54, 56]), 1st Trimester placenta (PRJEB28266 [55, 57]) and 3rd Trimester fetal side Non-PE (control) placenta (28 gestational weeks (gw), in this study). **(b)** Latent time distribution in fetal and maternal sides of control placentas from 29gw to 38gw. **(c)** Latent time distribution in fetal and maternal sides of PE placentas from 32 to 36gw.


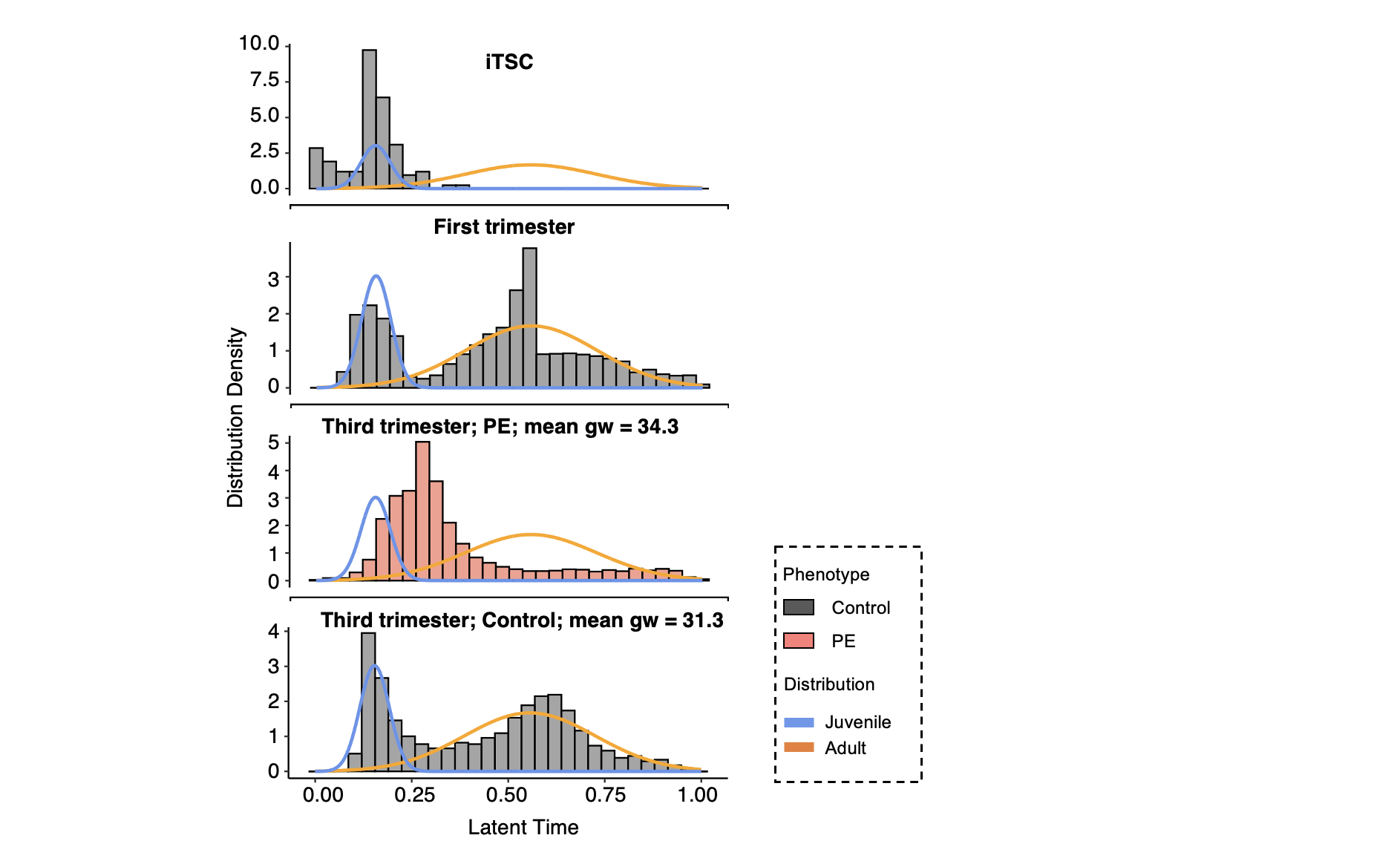


**Fig. S10. Latent time of trophoblasts, grouped by trimester.**

Latent time distribution in iTSC (GSE150578 [58, 59]), first trimester samples (PRJNA492324 [54, 56] and PRJEB28266 [55, 57]), third trimester PE placentas (mean gestational week is 34.3) and third trimester control placentas (mean gestational week is 31.3).


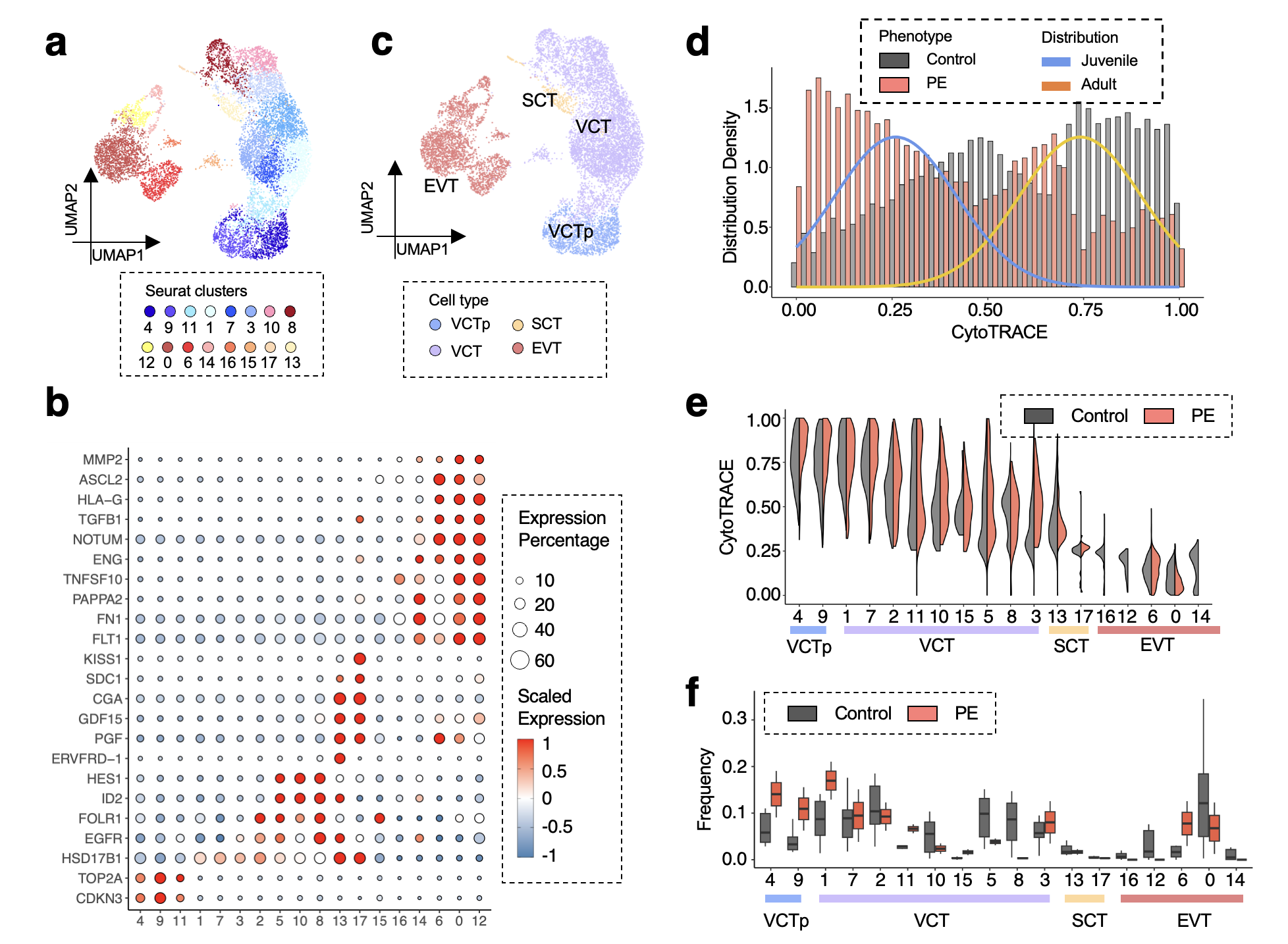


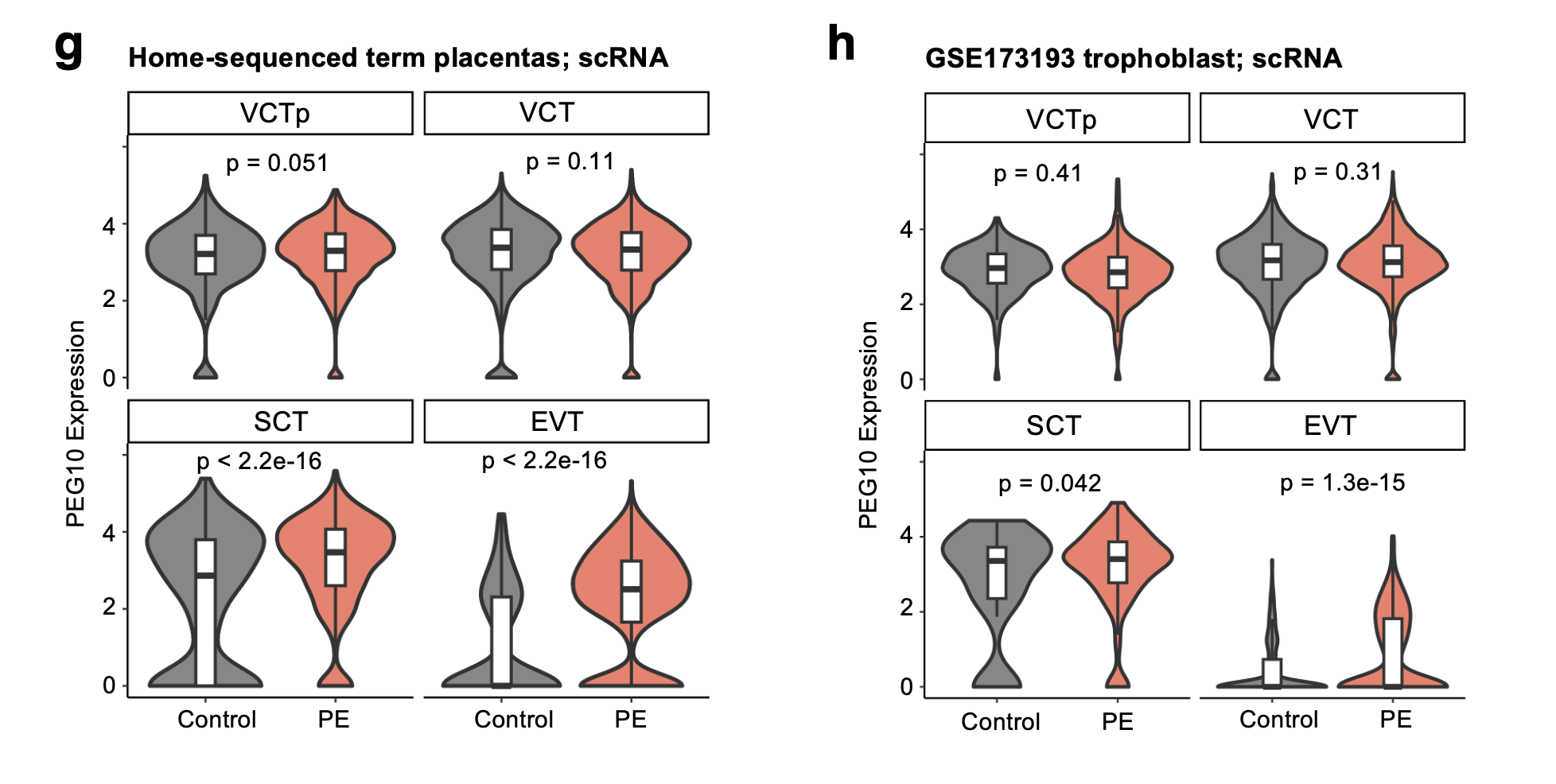


**Fig. S11. scRNA analysis of trophoblast cells in external validation dataset.**

**(a)** scRNA single cell trophoblast clusters in external validation dataset (GSE173193 [70]). **(b)** Canonical marker gene expression in each single cell cluster as in **(a)**. **(c)** Gross cell type annotation of the same cells in **(a)**. **(d)** The distribution of CytoTRACE score in control (grey) and PE (red) trophoblasts. CytoTRACE score of control trophoblast was decomposed into two distributions by mixtools, ‘Adult’ distribution (yellow) has lower CytoTRACE score, representing differentiated cells, while ‘Juvenile’ distribution (blue) with higher CytoTRACE score, indicating trophoblasts with strong stemness potential. **(e)** CytoTRACE score of PE and control cells in single cell clustered ordered from left to right according to their average CytoTRACE score. **(f)** Cell frequency in each single cell cluster for PE and control. **(g)** scRNA expression of *PEG10* in each subtype of trophoblast in in-house sequenced trophoblast as shown in Fig.2b. P-values were tested by t-test. **(h)** scRNA expression of *PEG10* in each subtype of trophoblast in GSE173193 trophoblast. P-values were tested by t-test.

**
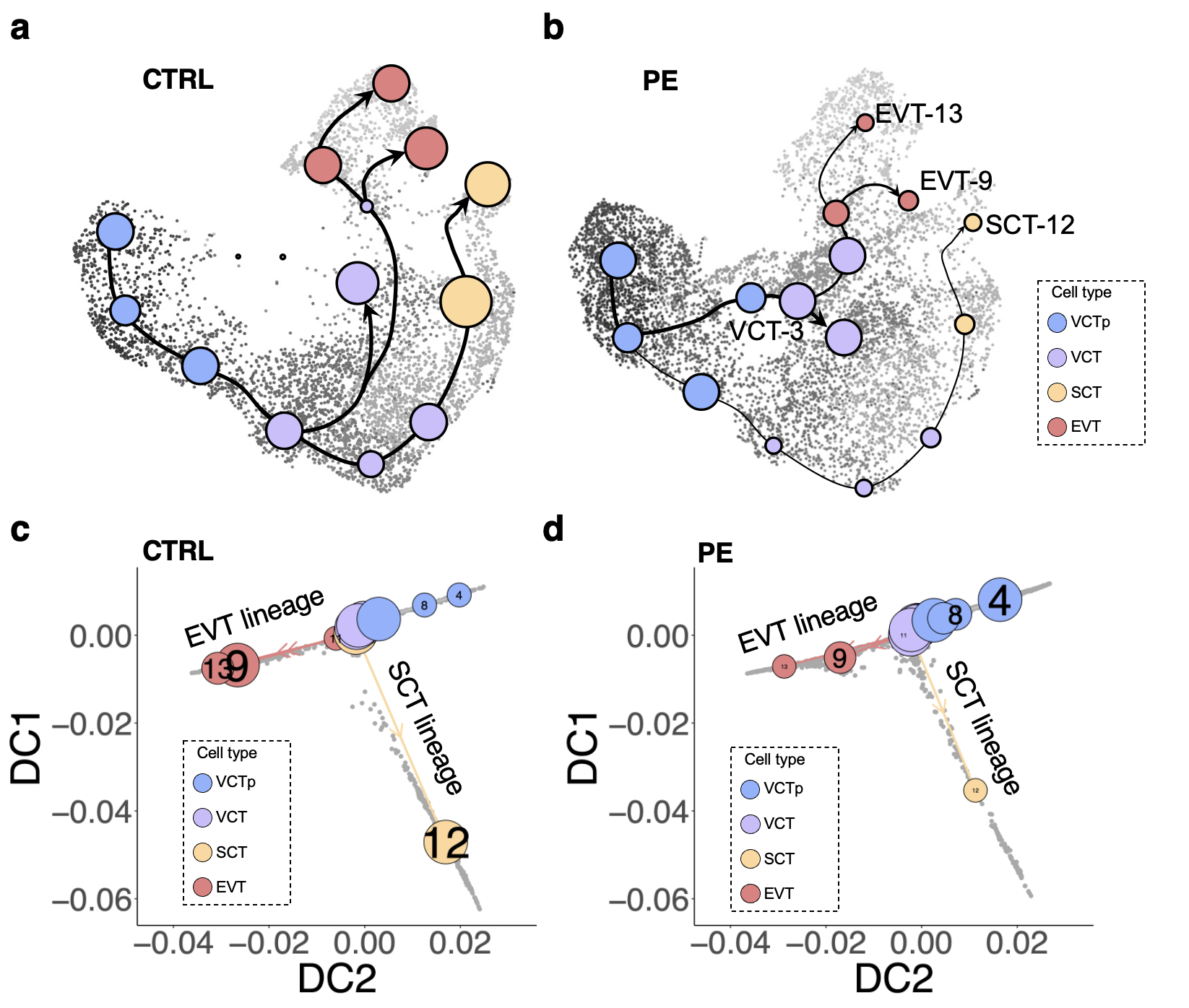
**

**Fig. S12. Developmental trajectory switch in PE trophoblast.**

**(a-b)** Schematic diagrams summarized trophoblast developmental trajectories on UMAP projection in control **(a)** and PE **(b)**, the colors of dots indicate trophoblast cell types. Size of dots and lines are scaled by cluster frequency. Arrows indicate directions of trajectories. Comparing with control, developmental trajectories in PE switched from VCTp8 to differentiate into VCTp14, and differentiation towards EVT/SCT are attenuated. **(c-d)** Slingshot-inferred developmental trajectories on diffusion map projection in control **(c)** and PE **(d)**. X-axis, diffusion component (DC) 2 and Y-axis, DC1. Diffusion components are the eigenvectors in diffusion map analysis. Dots represent general trophoblast cell types as in **(a-b)** and labelled with number. Arrows represent directions of trajectories towards terminal lineages: SCT or EVT.


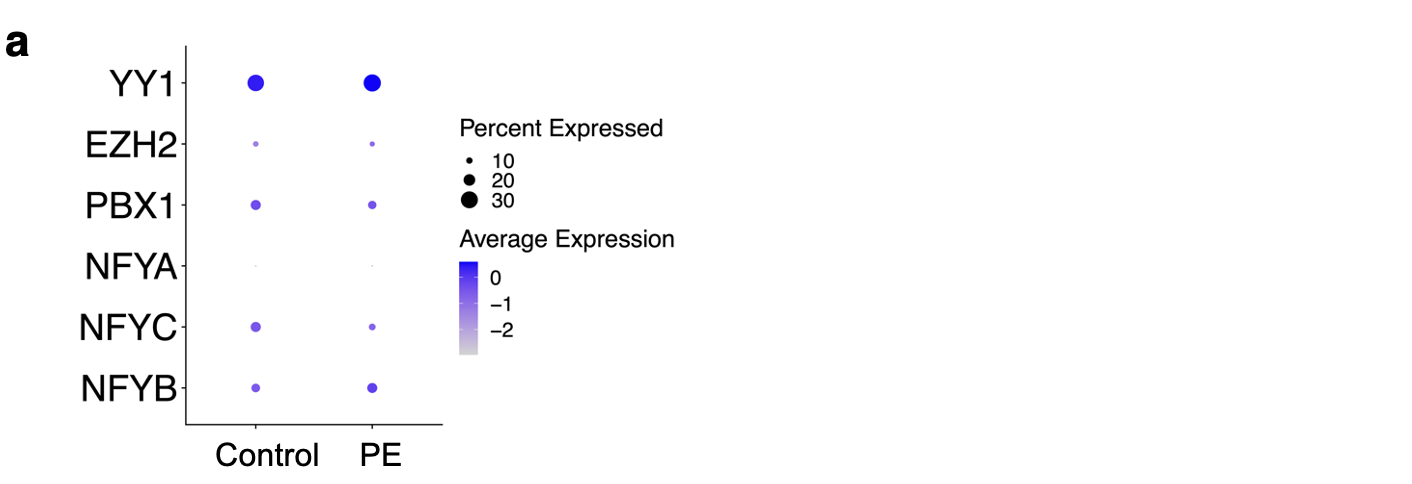


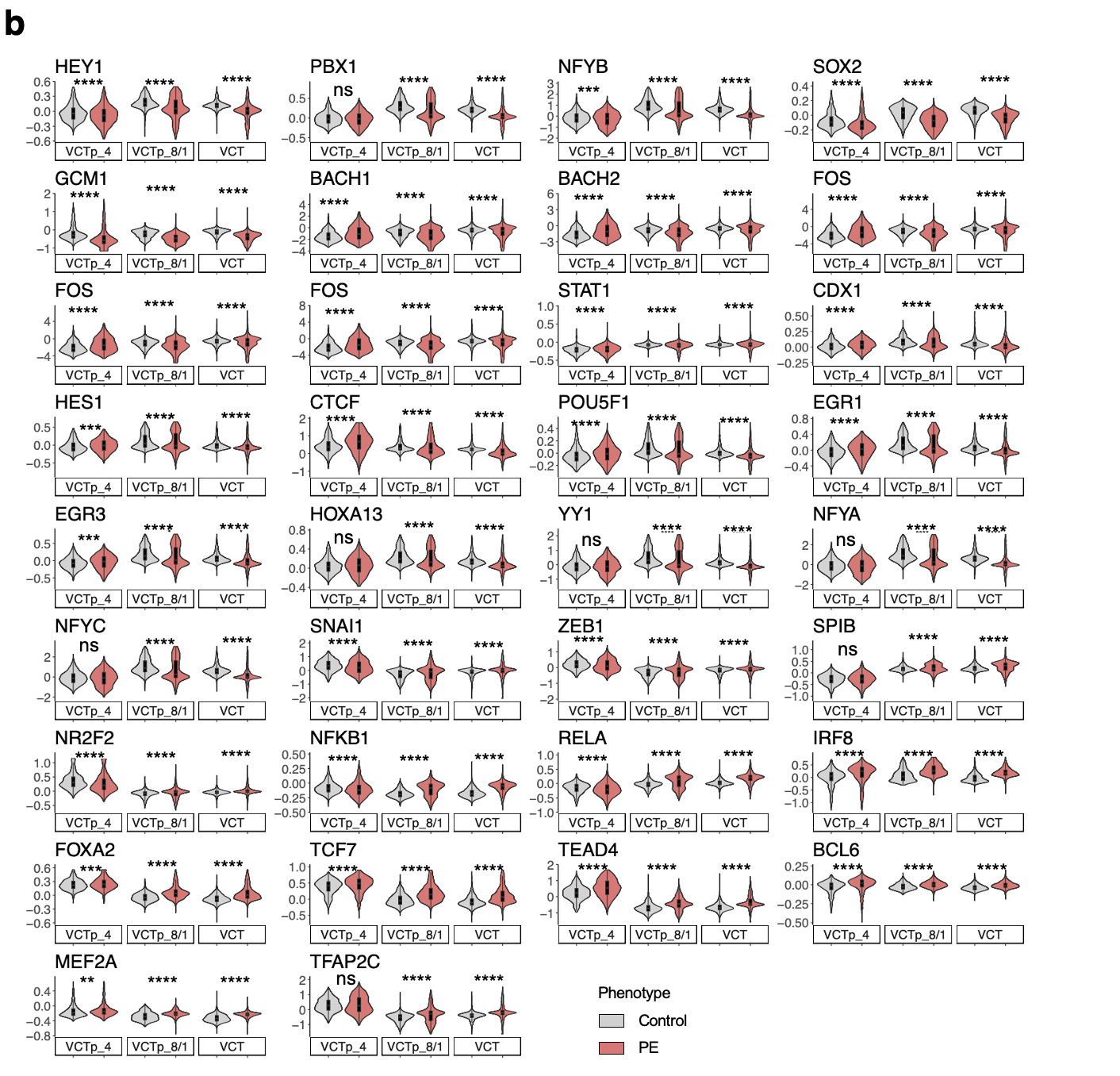


**Fig. S13. RNA expression and transcription factor binding activity of master transcription factors (TF).**

**(a)** RNA expression of master TFs between control and PE trophoblast. No significant difference in transcription level of master TFs between control and PE trophoblast. **(b)** TF binding activity between PE and control VCTp or VCT, measured by scATAC. P-values were tested by t-test (ns: not significant, *: P<0.05, ** P<0.01, ***: P<0.001).


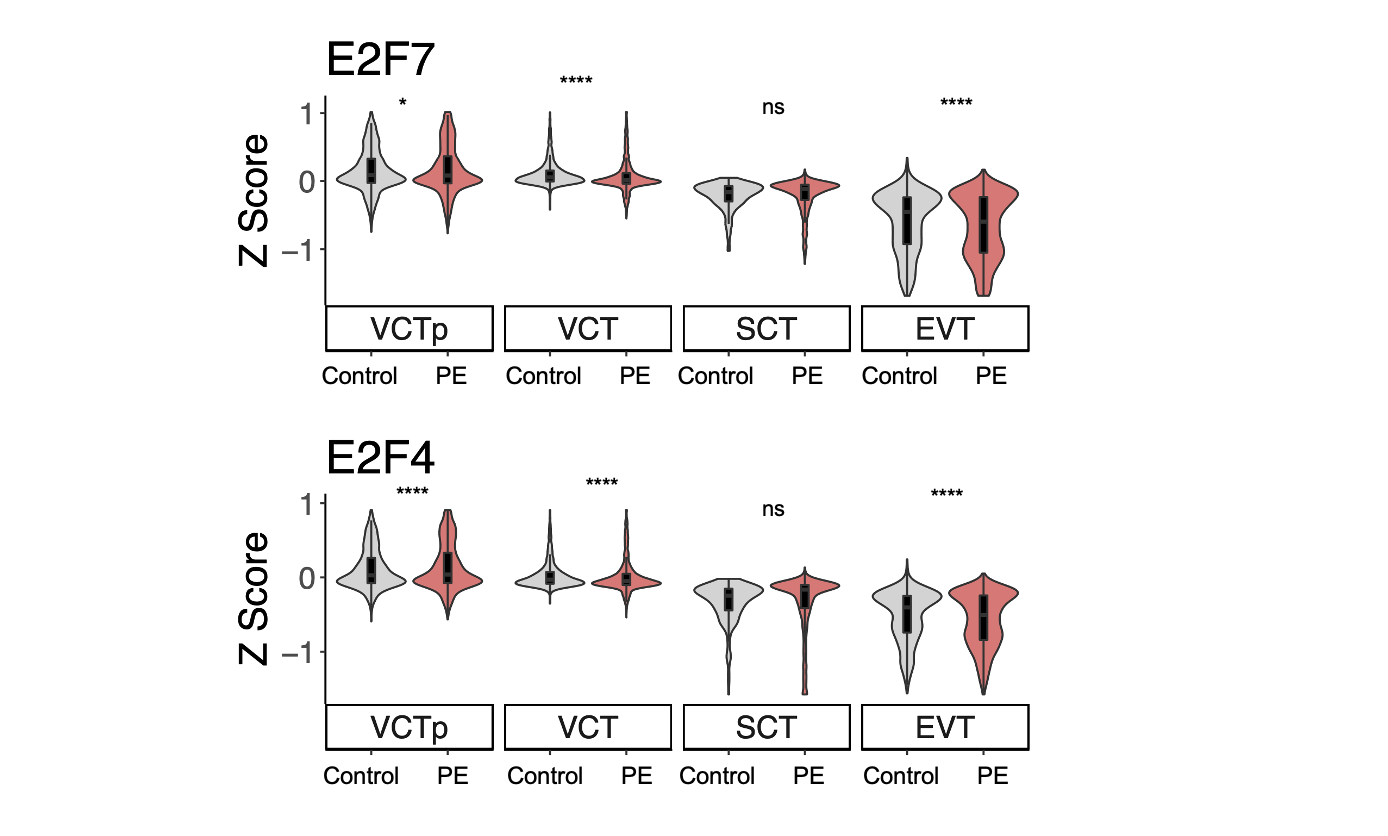


**Fig. S14. Transcription factor activities upstream of EZH2 in PE placenta.**

The Z-score of transcription factor activities of EZH2 upstream TF E2F7 and E2F4 in control and PE placenta samples. Single cell Z-scores are shown. Gray: single cells from control placenta. Red: single cells from PE placenta. P-values were tested by t-test (ns: not significant, *: P<0.05, ** P<0.01, ***: P<0.001, ****: P<0.0001).


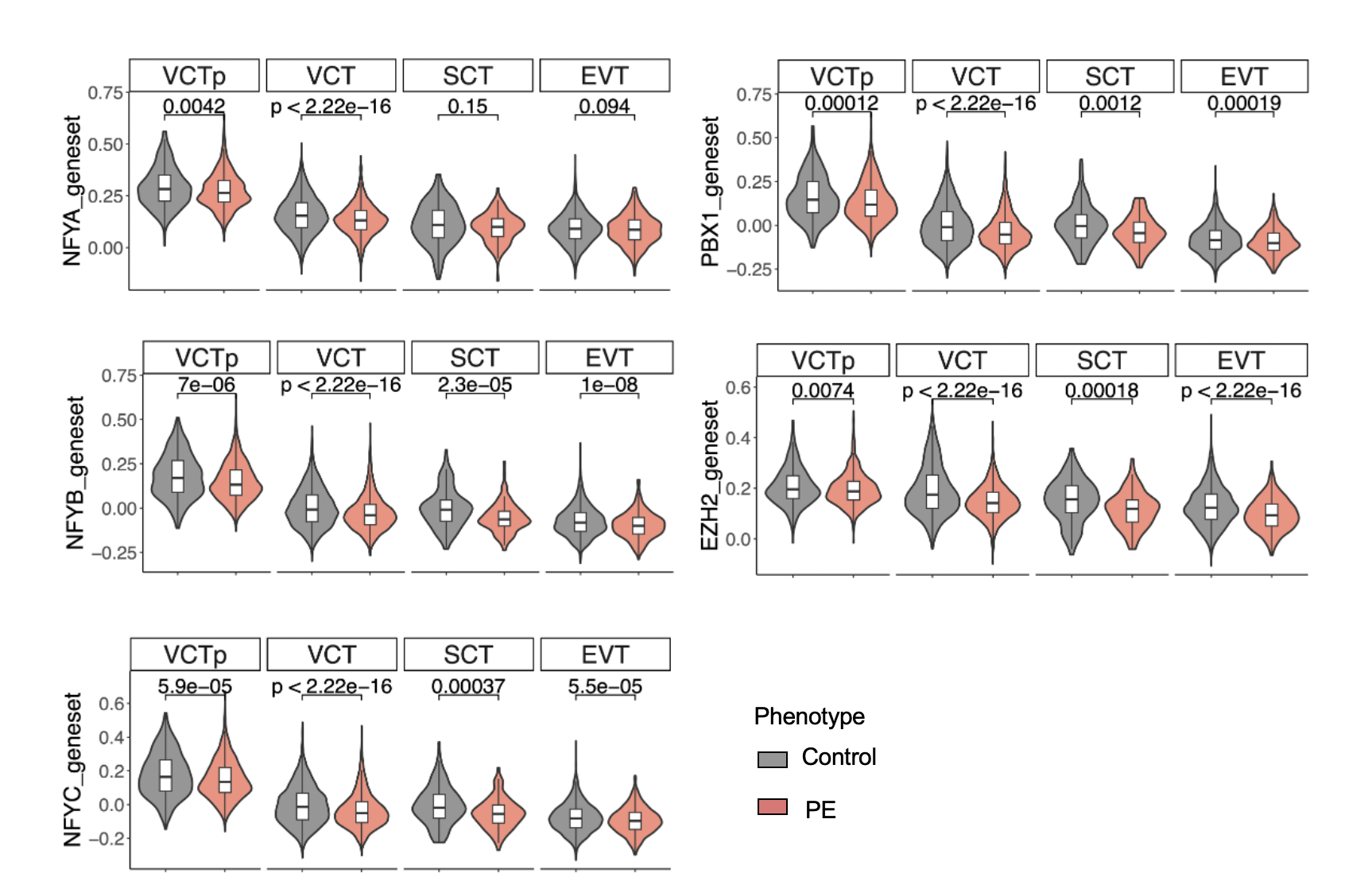


**Fig. S15. Master transcription factor controlled velocity gene set expression in the trophoblast cells in external validation dataset**.

Expression of master transcription factor gene sets described in Fig. 3 between control and PE trophoblast in external validation dataset (GSE173193 [70]). P-values were tested by t-test.


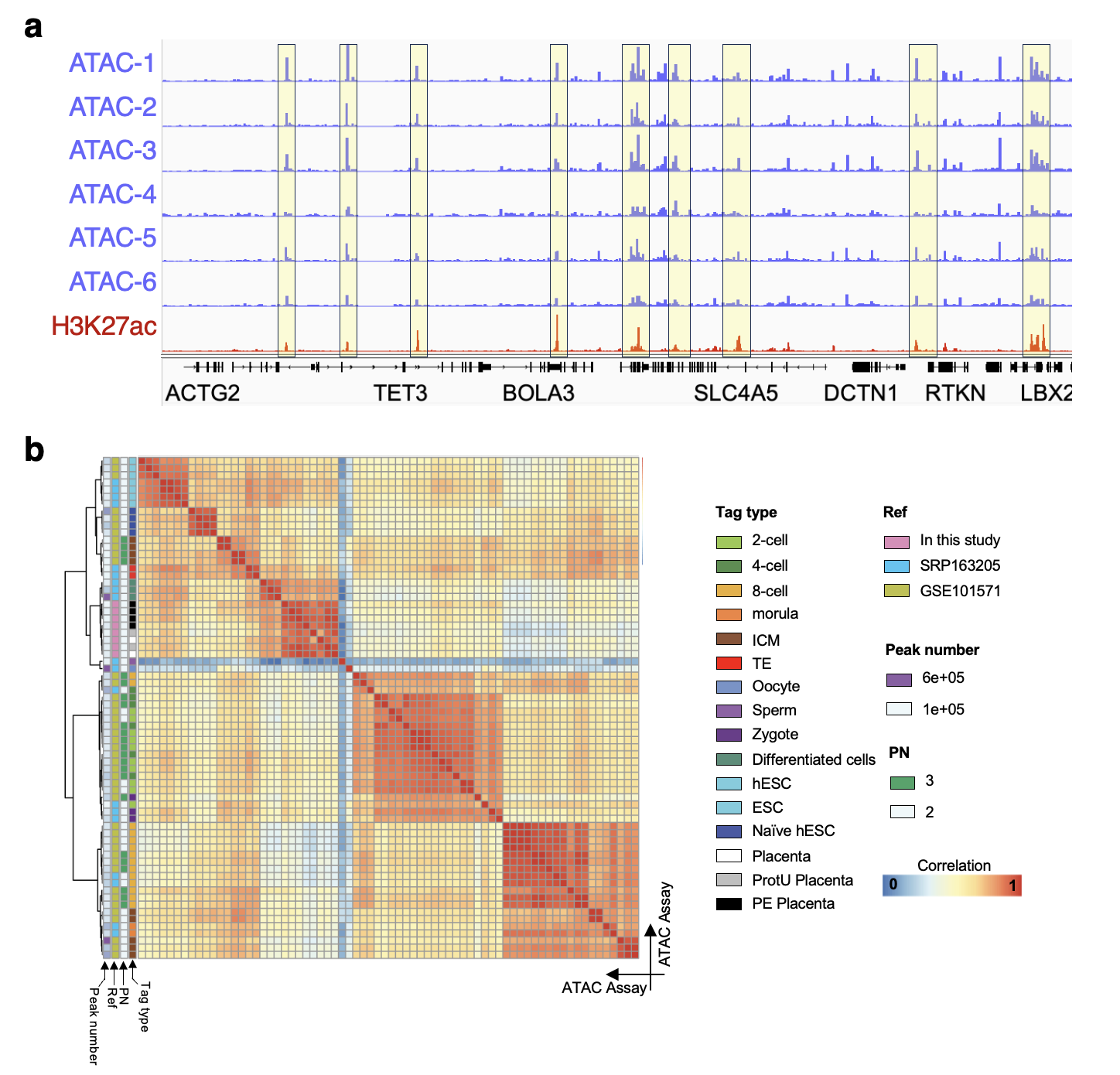


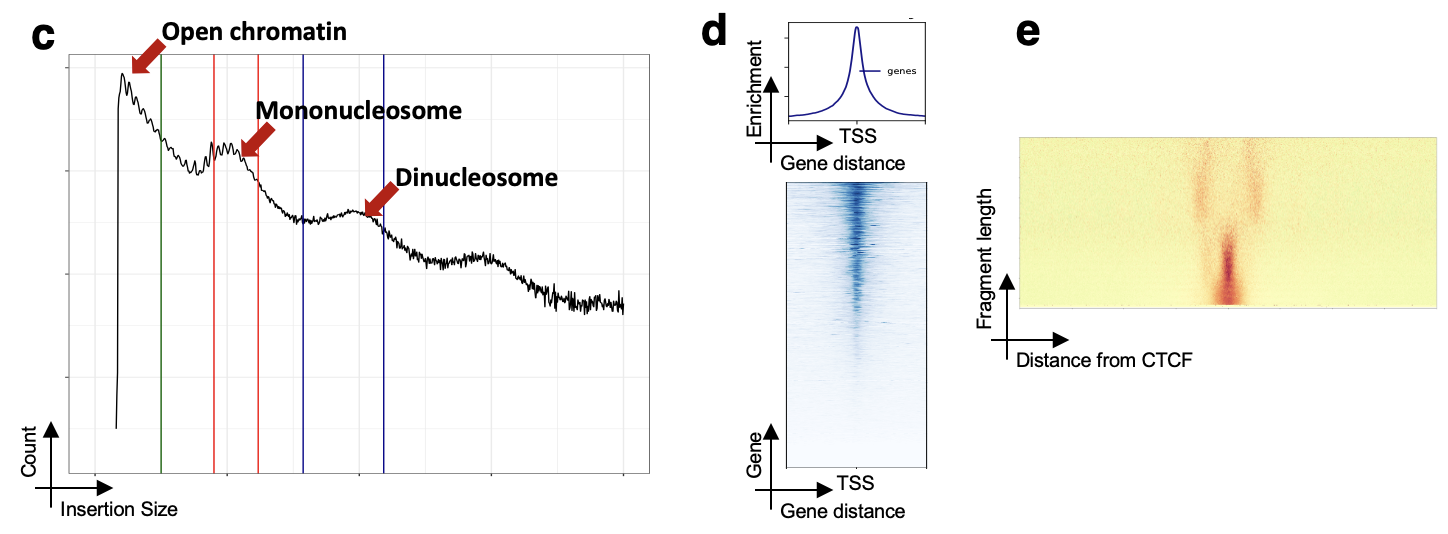


**Fig. S16. Bulk ATAC seq assay on frozen placenta tissue.**

**(a)** Placenta ATAC-seq signals overlap with activating histone mark (H3K27ac). Bulk placenta ATAC-seq data in this study shown as dark grey and H3K27ac ChIP-seq data of placenta from Roadmap Project (GSM1127147) shown as red. Majority of H3K27ac peaks overlapped with placenta ATAC-seq peaks, shown in light orange shades, indicating that placenta ATAC-seq peaks marked open chromatin with active transcription. **(b)** Open chromatin accessibility distinguishes early developmental stages of human embryos and pathological state of placentas. Per-sample correlation of single cell ATAC-seq peaks from zygote, SRP163205 [45] and GSE101571 [181]; 2-cell, GSE101571 [181]; 4-cell, SRP163205 [45] and GSE101571 [181]; 8-cell, SRP163205 [45] and GSE101571 [181]; morula, SRP163205 [45]; ICM, GSE101571 [181]; trophectoderm (TE), SRP163205 [45]; hESC, SRP163205 [45] and GSE101571 [181] and bulk placenta ATAC-seq peaks from preeclampsia and non-preeclampsia placenta were shown. Overall, samples of similar developmental stage and pathological state were clustered together. Samples from pre-ZGA, and post-ZGA, clustered together regardless of study origin, suggesting that these ATAC-seq data were sufficiently robust and batch effect were minimal. Preeclampsia and non-preeclampsia placenta clustered separately. Preeclampsia placentas (placenta PE, black) had distinctive ATAC peak profile comparing with non-preeclampsia placentas (placenta and placentaProU, from pregnancies with positive proteinuria but no hypertension). Placentas from pregnancies with proteinuria but not preeclampsia (placentaProU, grey) are indifferent from placentas from normal pregnancy (placenta, white) based on bulk ATAC-seq peaks. Labels on the side: tagtype: embryonic developmental stage or pathological state. ICM: inner cell mass, TE: trophectoderm, differentiated_cells: cells differentiated from hESC; Placenta: non-preeclampsia normal placenta; PlacentaProtU: placenta from proteinuria, non-preeclampsia pregnancy; PlacentaPE: placenta from preeclampsia pregnancy. PN: pronucleus (for embryonic development single-cell ATACseq data, as in the original publication). **(c)** Read length distribution from an example placenta ATAC-seq data, showing characteristic peaks of fragment length at open chromatin, mononucleosome, dinucleosome, and trinucleosome, suggesting that this bulk ATAC-seq protocol successfully captures the original chromatin structure. **(d)** TSS-enrichment (sequencing depth around +- 2kbp of transcription start site) of an example placenta ATAC-seq library, showing enrichment at 0bp of TSS >30-fold compared to boundary (+-2kbp). **(e)** V-plot around CTCF binding site on an example placenta ATAC-seq data, showing characteristic histone and CTCF binding footprints.


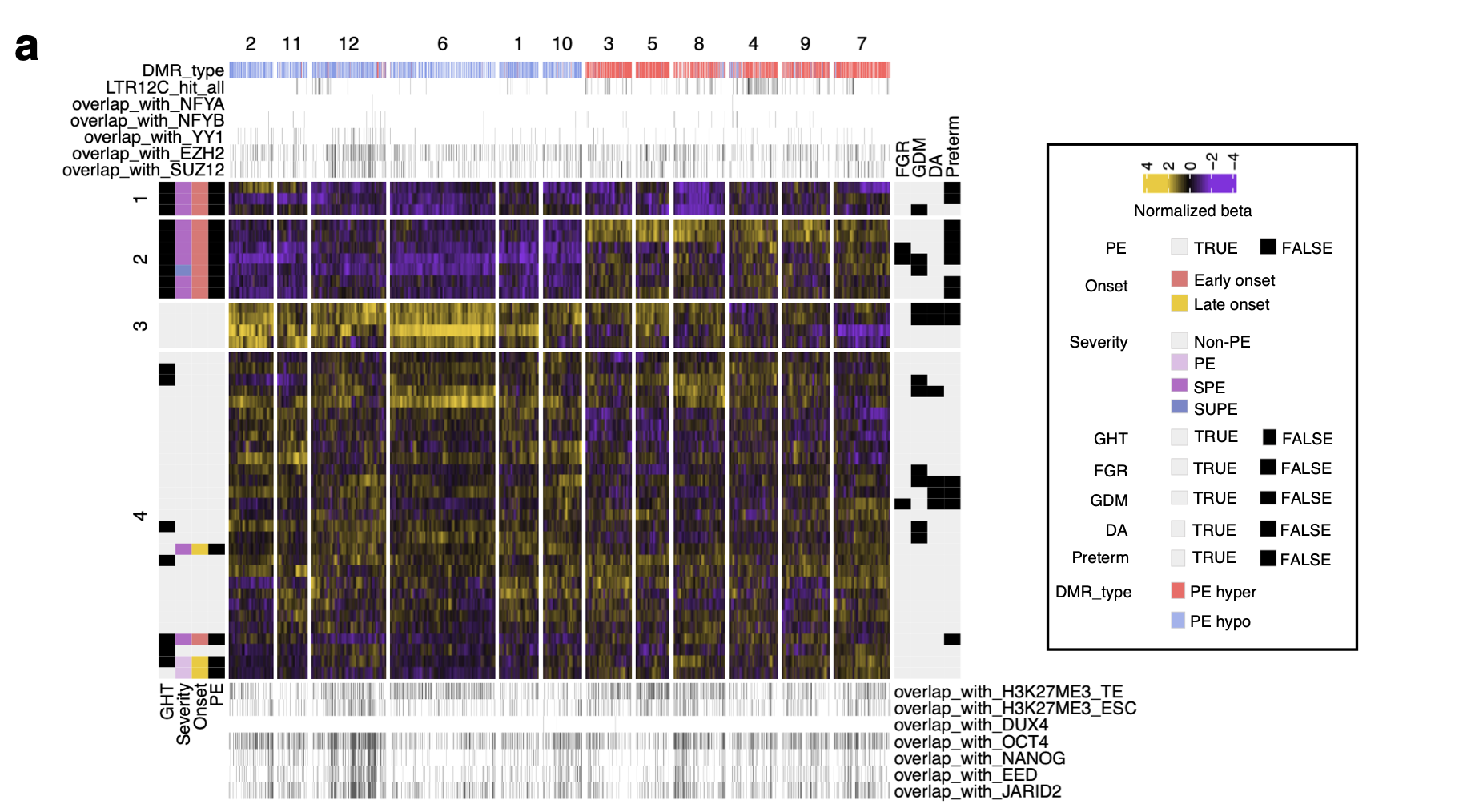


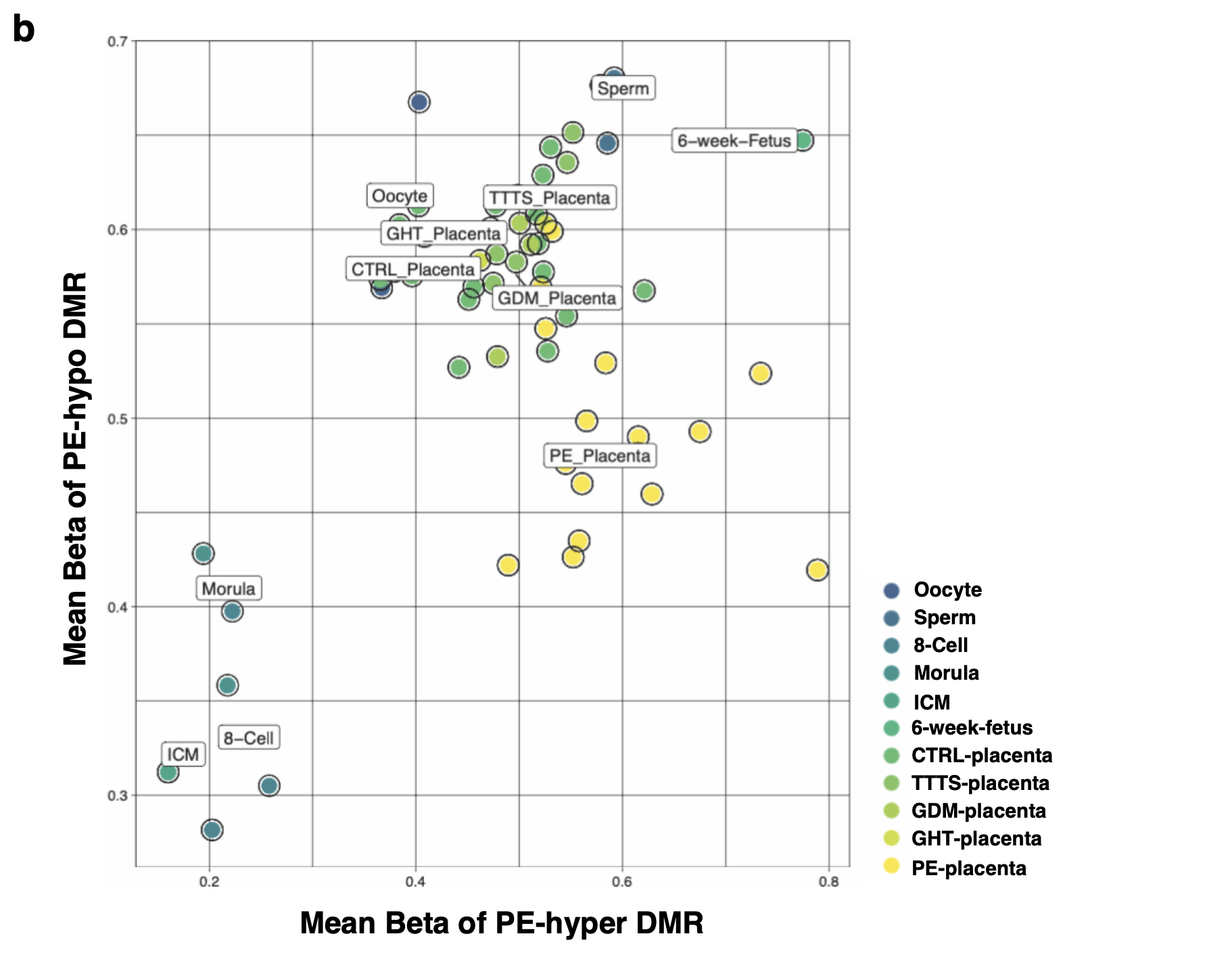


**Fig. S17. Differential DNA methylation between control and PE.**

**(a)** Heatmap of normalized beta value on PE-specific DMR including both preeclampsia-hyper (PE-hyper) DMR and preeclampsia-hypo (PE-hypo) DMR across 43 placenta samples from preeclampsia (PE), fetal growth restriction (FGR), gestational diabetes mellitus (GDM), gestational hypertension (GHT), chronic hypertension (HT), chronic diabetes mellitus (DM), preterm birth, diamniotic pregnancy (DA) pregnancies, or ‘normal’ pregnancies without above complications. Preeclampsia placentas can be distinguished from non-preeclampsia placentas based on DNA methylation profile. Unsupervised hierarchical clustering showed clear divergence of PE-hyper and PE-hypo DMR across samples. PE-hyper DMR were enriched with LTR12C elements. PE-hypo DMRs was highly enriched with PRC2 binding (EZH2/EED/JARID2/SUZ12/YY1), H3K27me3 modification in ESC and trophectoderm, and pluripotency factor (OCT4/NANOG) binding. **(b)** Mean methylation level of preeclampsia specific DMR could distinguish preeclampsia placenta to non-preeclampsia placenta. X axis: Mean beta of PE-hyper DMR from each sample; y axis: Mean beta of PE-hypo DMR from each sample. Single cell WGBS from oocyte, sperm, 8-cell stage, morula, ICM, 6-week fetus, and bulk DNAm-seq of placenta from normal (control), twin-twin transfusion-syndrome (TTTS), gestational diabetes (GDM), gestational hypertension (GHT), or preeclampsia pregnancy (PE) were shown in the figure. Mean beta were calculated as the mean methylated CpG fraction in DMR. As shown in the figure, most PE placenta could be distinguished from all other placentas by hypermethylated PE-hyper DMR and hypomethylated PE-hypo DMR.


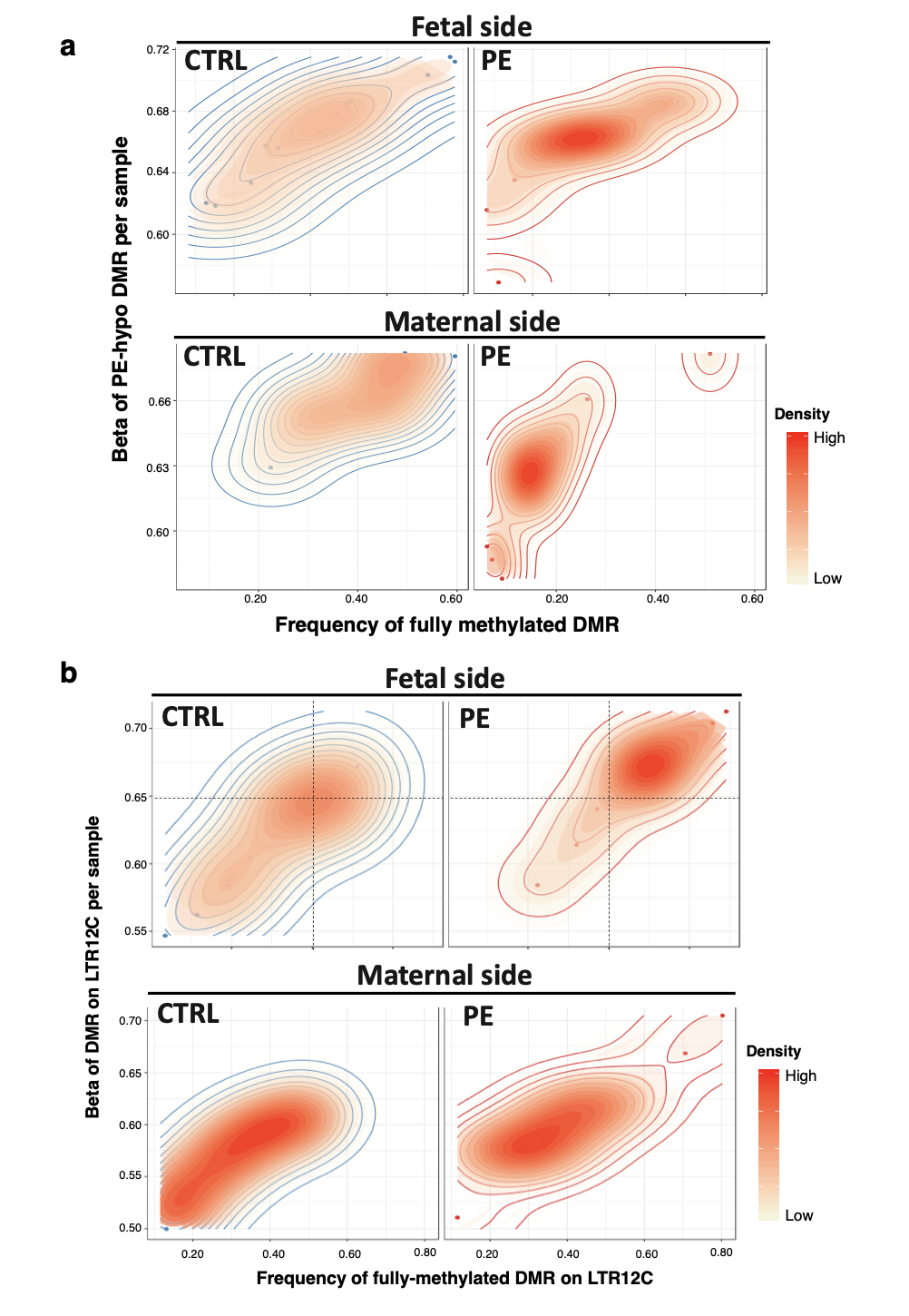


**Fig. S18. Differential methylation in fetal and maternal face of placenta between PE and control.**

**(a)** Mean beta level (Y-axis) of PE-hypo DMR, and frequency of fully-methylated PE-hypo DMR (X-axis), on control (left panels) and PE (right panels) placenta fetal (top panels) and maternal (bottom panels) face. Only PE placenta maternal face shows a significant shift of PE-hypo DMR hypomethylation, suggesting that these hypomethylation occurs in SCT/EVT-rich anatomical regions. **(b)** Mean beta level (Y-axis) of LTR12C DMR, and frequency of fully-methylated LTR12C DMR (X-axis), on control (left panels) and PE (right panels) placenta fetal (top panels) and maternal (bottom panels) face. Only PE placenta fetal face shows a significant shift of LTR12C hypermethylation, suggesting that these hypermethylation occurs in VCT-rich anatomical regions.


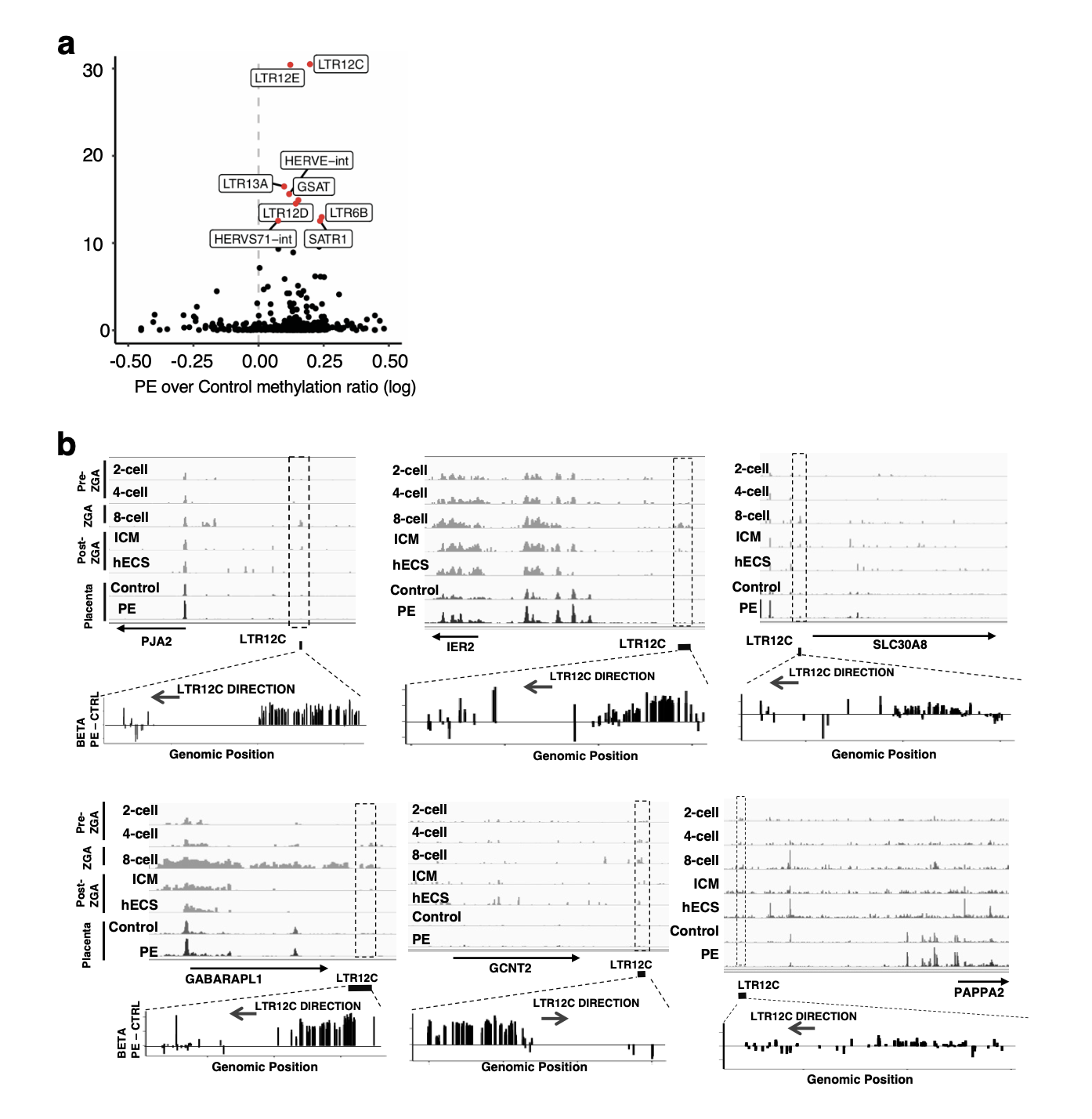


**Fig. S19. Deficient ExE-specific *de novo* methylation in paternally imprinted loci in PE placenta.**

**(a)** LTR12 family retrotransposon are hypermethylated in preeclampsia placenta. X-axis: log-ratio of mean methylation level of preeclampsia placenta over normal placenta for individual class of repeat element; Y-axis: Fractions of repeats containing preeclampsia-specific DMR. Red dots with names are repeats with significant enrichment (Fisher’s exact test P<0.05) of DMR. **(b)** Preeclampsia-hypermethylated LTR12C around velocity genes *PJA2*, *IER2*, *SLC30A8*, *GABARAPL1*, *GCNT2* and *PAPPA2* were shown in the figure. Top: ATAC-seq of 2-cell, 4-cell, 8-cell, ICM, hESC, PE and control Placenta. Bottom: Methylation difference (beta: PE- control) on the loci, shown together with LTR12C genomic direction. These data showed that preeclampsia- hypermethylated LTR12C were commonly hypermethylated in 5’ transcriptional start regions. These LTR12C were most active around ZGA (8-cell stage) and silenced post-ZGA.


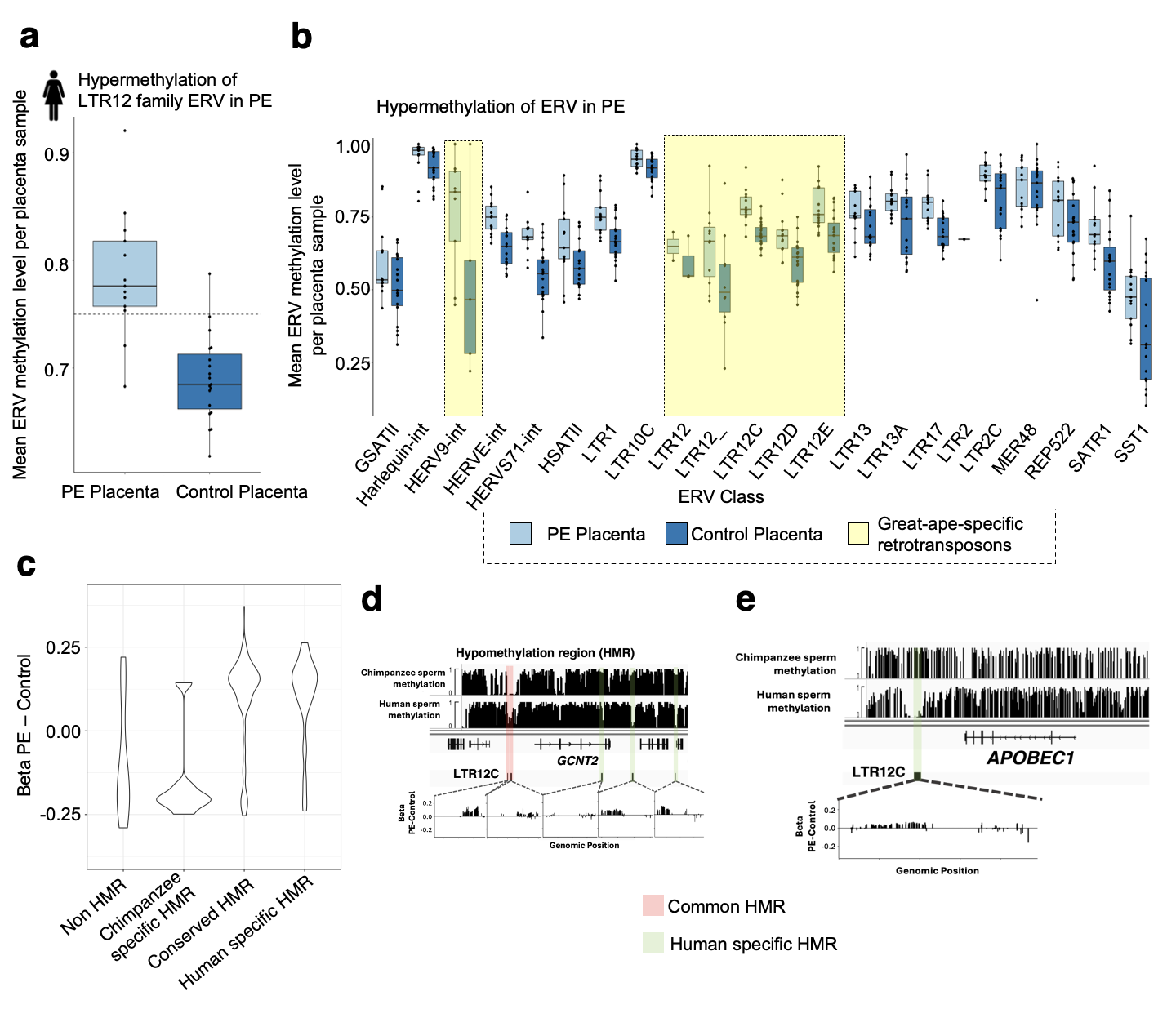


**Fig. S20. DNA methylation levels on recently evolved, primate-specific retrotransposons, particularly the imprinted LTR12C, discriminate PE and control placenta.**

**(a)** Mean beta level (Y-axis) of LTR12C elements clearly distinguish PE (light blue) and control (navy) placenta in human. **(b)** Mean beta level (Y-axis) of all repeat masker-annotated class of ERV in PE (light blue) and control (navy) placenta. Great-ape-specific retrotransposons are highlighted in yellow. **(c)** Mean beta level difference (Y-axis) between PE and control placenta of non-LTR12C or LTR12C elements, classified by their DNA methylation status in male gamete (sperm), as chimpanzee-specific (hypomethylated only in chimpanzee sperm), human-specific (hypomethylated only in human-sperm), or conserved (hypomethylated in both chimpanzee and human sperm). Only LTR12C that are hypomethylated in human sperm are found to be hypermethylated in PE placenta, suggesting an interesting scenario that the imprinting mechanism might be dysfunctional on these loci in PE pregnancy. **(d-e)** Example sperm hypomethylated-region (HMR) (red: common, green: human-specific, not sperm HMR in chimpanzee) encompassing preeclampsia-hypermethylated LTR12C around *GCNT2* and *APOBEC1*. Black bars above: Methylation level (beta) of CpG loci in chimpanzee (top) or human (bottom) genomes. Chimpanzee data were mapped to human genome by LiftOver. Sperm-specific hypomethylation valley could be immediately identified visually on the figure. LTR12C loci were shown next to genes. Enlarged panels showing per-CpG methylation difference (PE - control) of each LTR12C loci in the sperm hypomethylation valley of human.


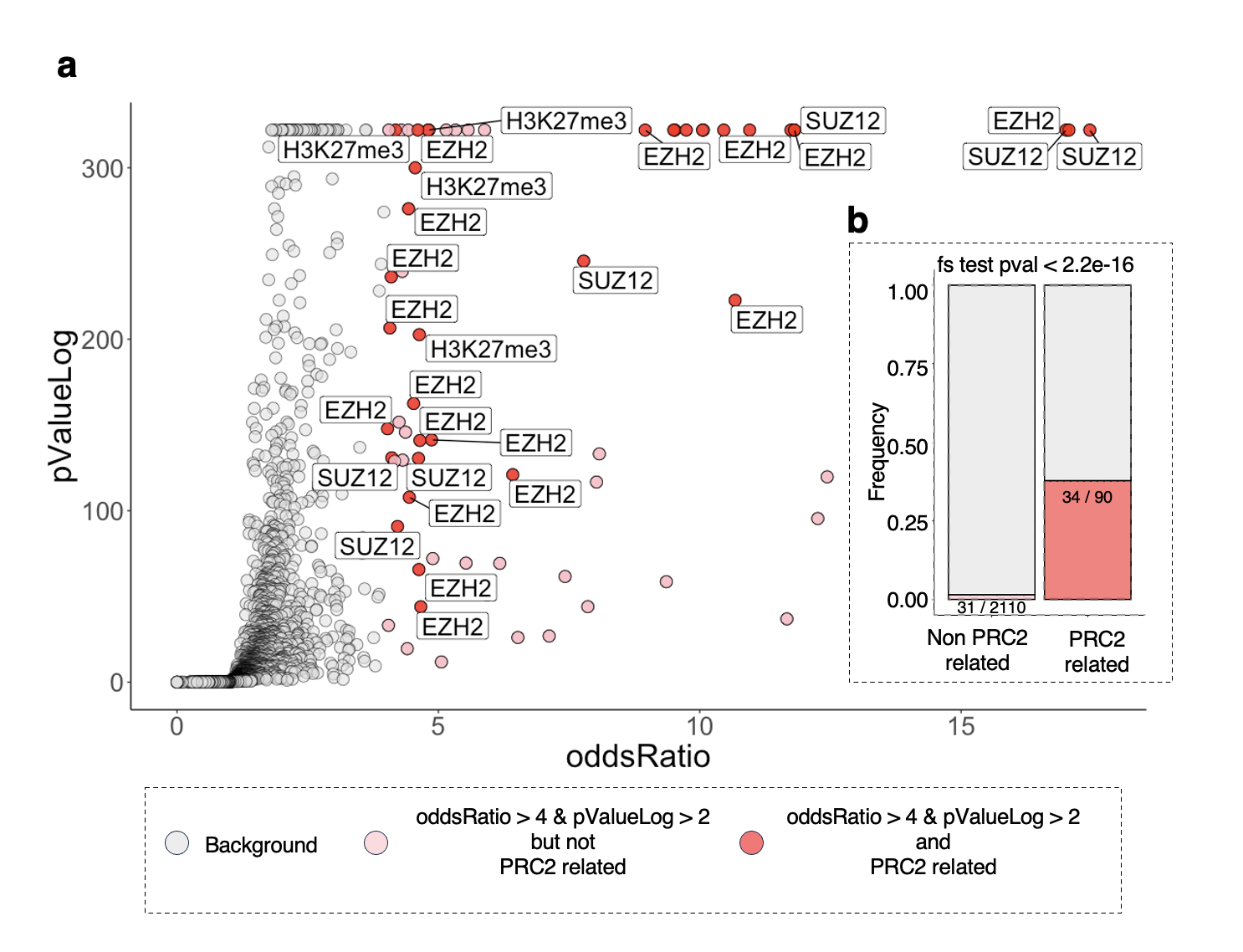


**Fig. S21. PE DMR regions are enriched with PRC2 related binding loci.**

Enrichment analysis by Locus Overlap Analysis (LOLA) on PE DMR regions using MSigDB and cross-tissue annotation of DNase hypersensitivity databases as reference. **(a)** Enrichment of PRC2 binding (including EZH2 and SUZ12) and H2K27me3 histone mark. **(b)** Significant enrichment of significant and PRC2 related loci to total PRC2 related loci than significant and non-PRC2 related loci to total non-PRC2 related loci, P value was calculated by Fisher's exact test. Grey: non significant loci (pValueLog < 2 or oddsRatio < 4); pink: significant (pValueLog > 2 and oddsRatio > 4) but non-PRC2 related loci; red: significant and PRC2 related loci.


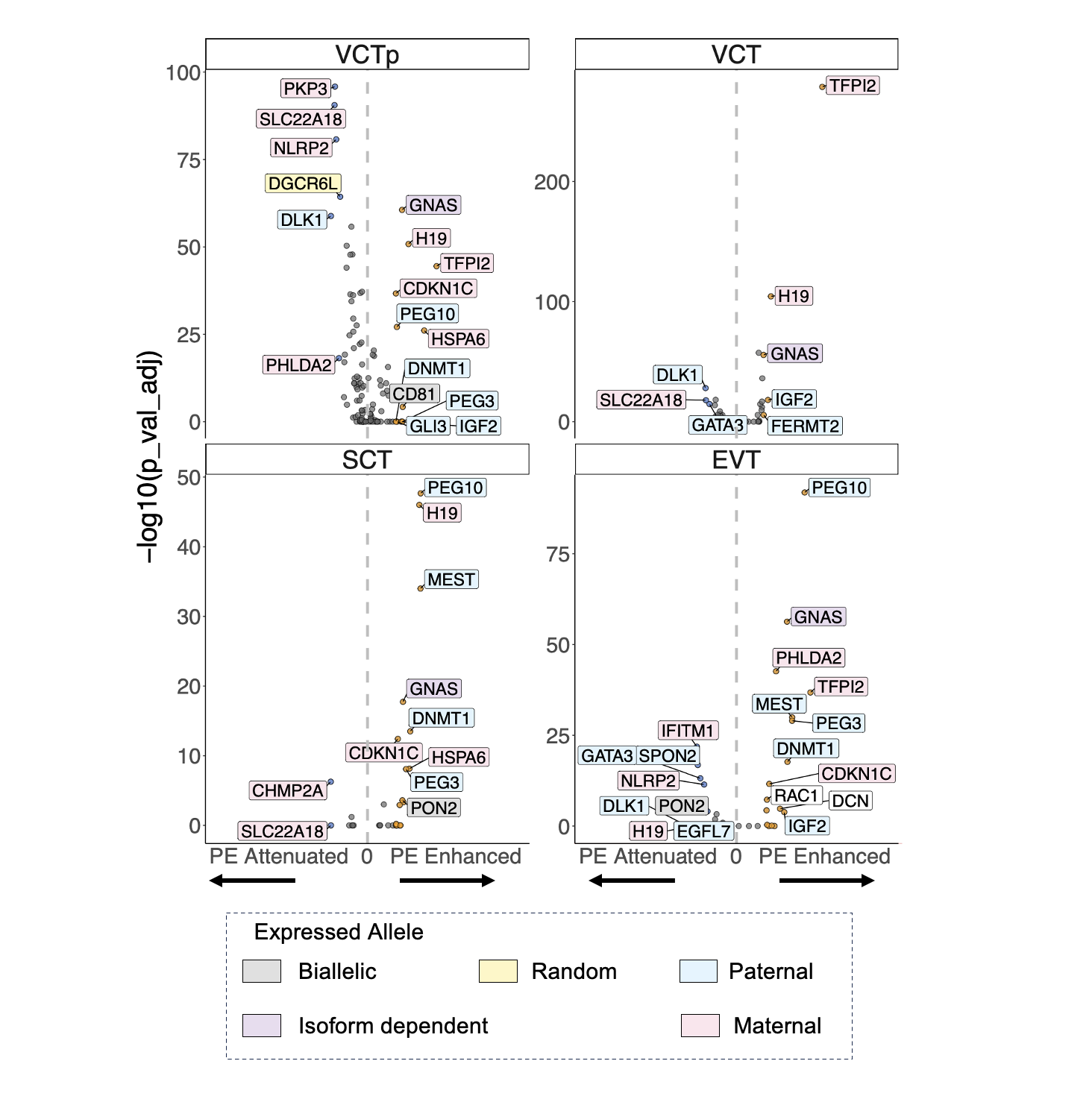


**Fig. S22. Differential expression of imprinted genes in trophoblast.**

Expression of imprinted genes in different trophoblast lineages between control and PE. Orange: genes with significantly enhanced expression in PE; blue: genes with significantly decreased expression in PE. Genes that are significantly differential expressed are labelled with gene name and colored with expressed allele.


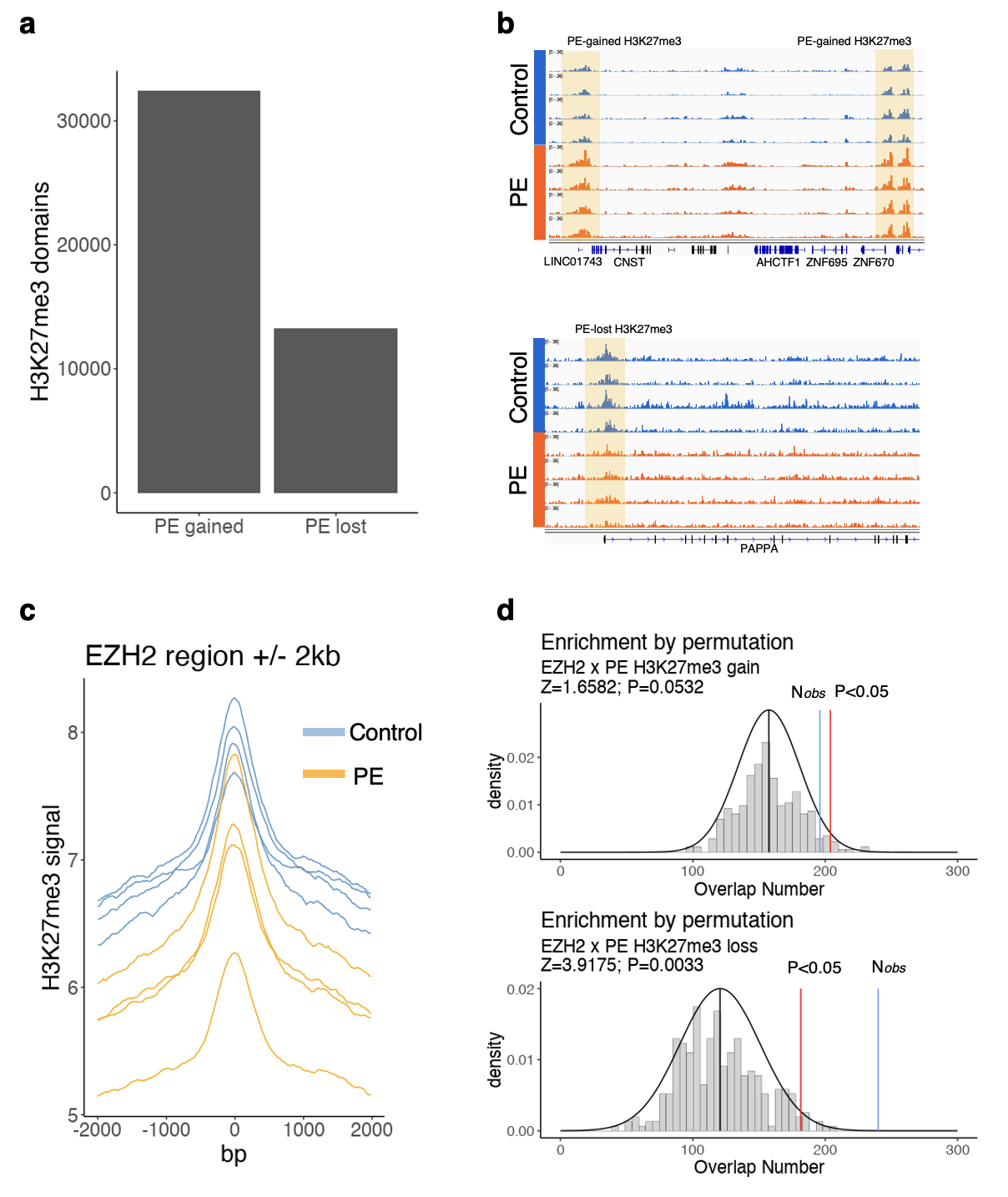


**Fig. S23. Reduced H3K27me3 modification on EZH2-controlled genes in PE placenta.**

**(a)** Number of PE-gained and PE-lost H3K27me3 domains identified by CUT&Tag. **(b)** Reduced and gained H3K27me3 in PE placenta. **(c)** H3K27me3 signal around +/- 2kbp of EZH2 binding sites. **(d)** Enrichment of EZH2 binding sites in PE-lost H3K27me3 domains but not PE-gained H3K27me3 domains. Permutation bootstrapping was performed in regioneR.

**
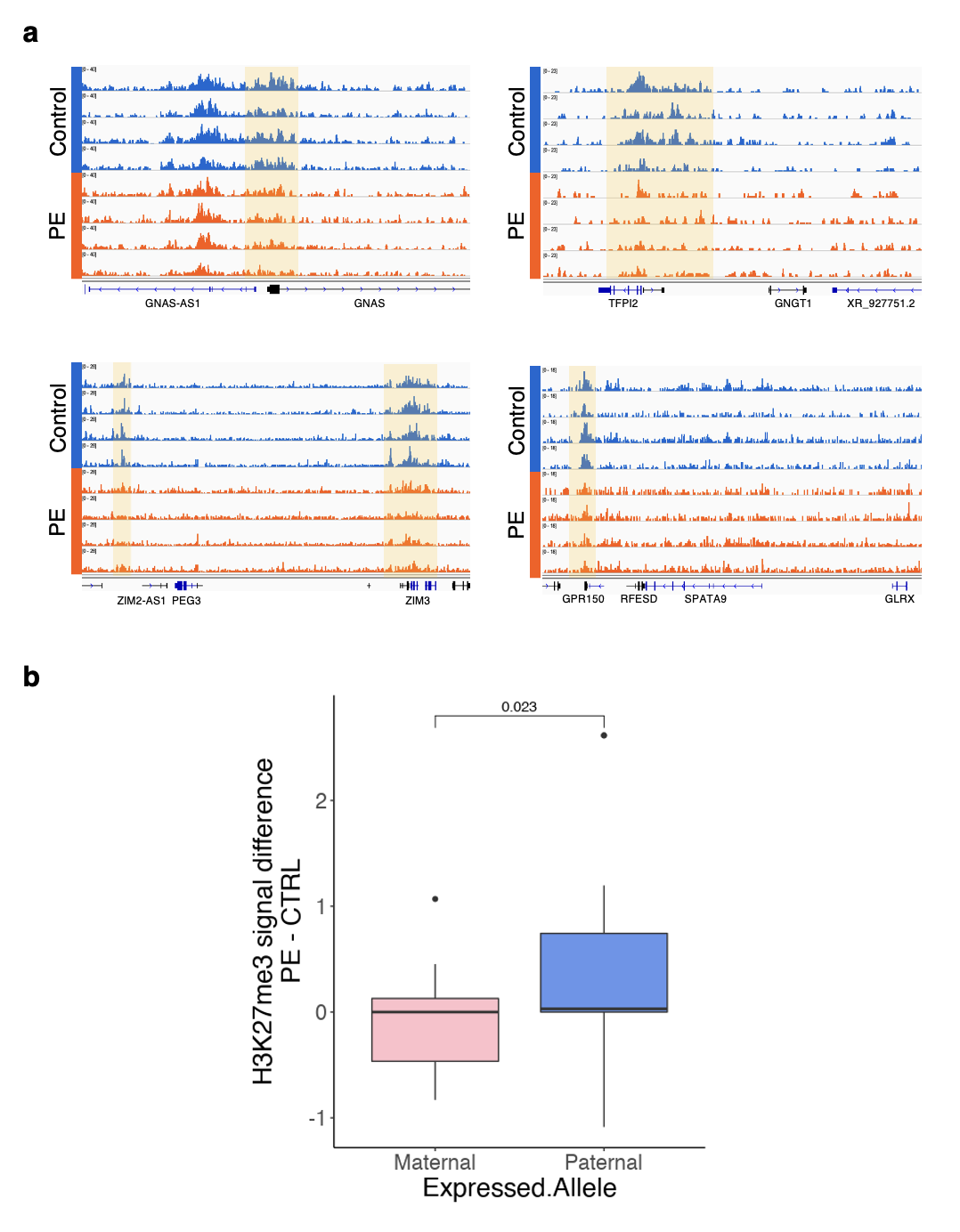
**

**Fig. S24. Reduced H3K27me3 modification on paternally imprinted genes in PE placenta.**

**(a)** Examples of H3K27me3 signal difference on imprinted genes. **(b)** paternally-imprinted genes (maternal allele expressed) are more likely to undergone PE-specific H3K27me3 lost. P-value was tested by t-test.


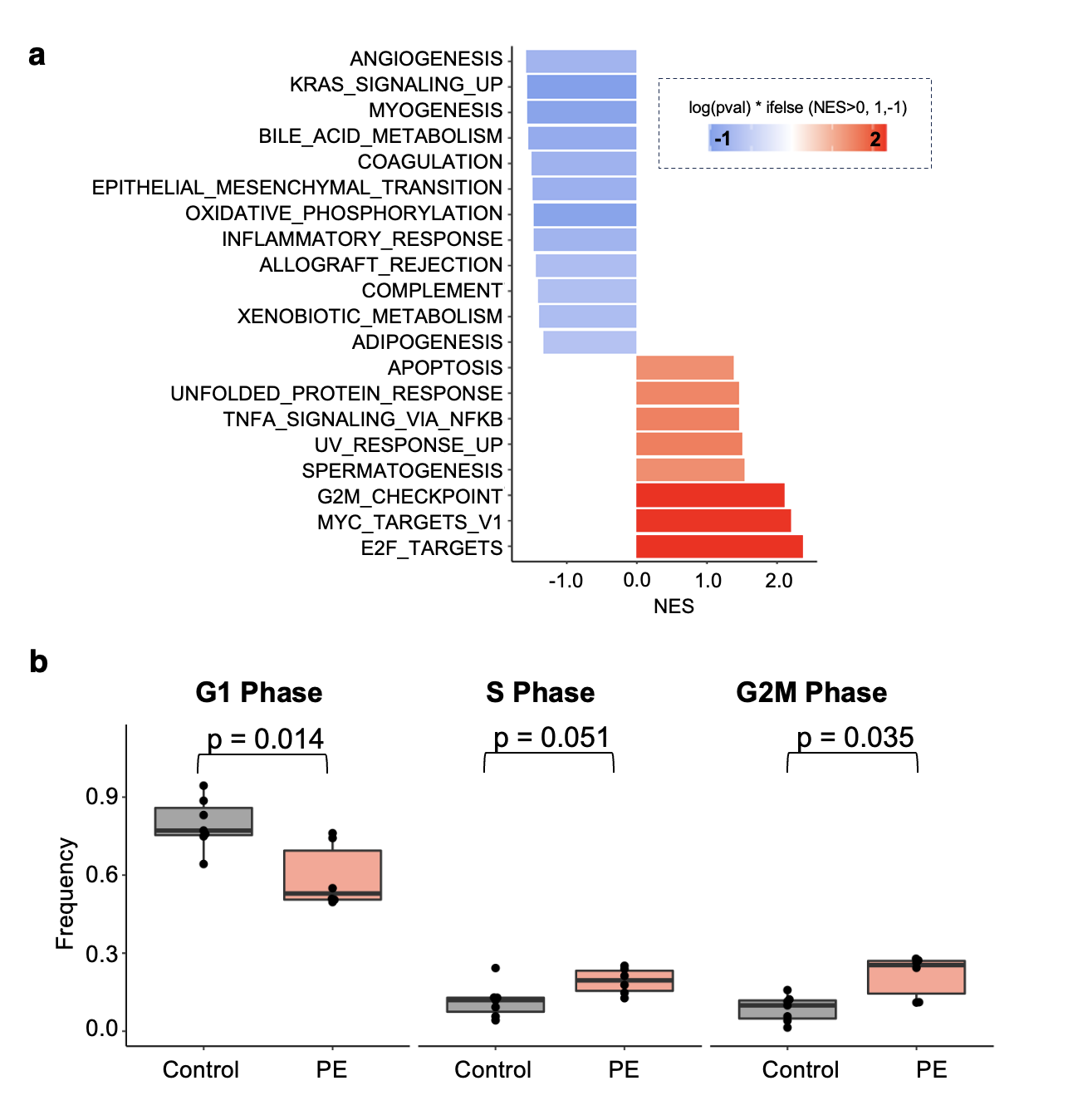


**Fig. S25. PE trophoblast overexpressed genes to stall its cell cycle progression.**

**(a)** GSEA analysis of the differentially expressed genes in PE trophoblast. **(b)** Percentage of cells in each cell cycle phase (G1, S, and G2/M) as scored by cell-cycle-related gene set expression in the scRNA dataset. P-values in **(b)** were tested by t-test.


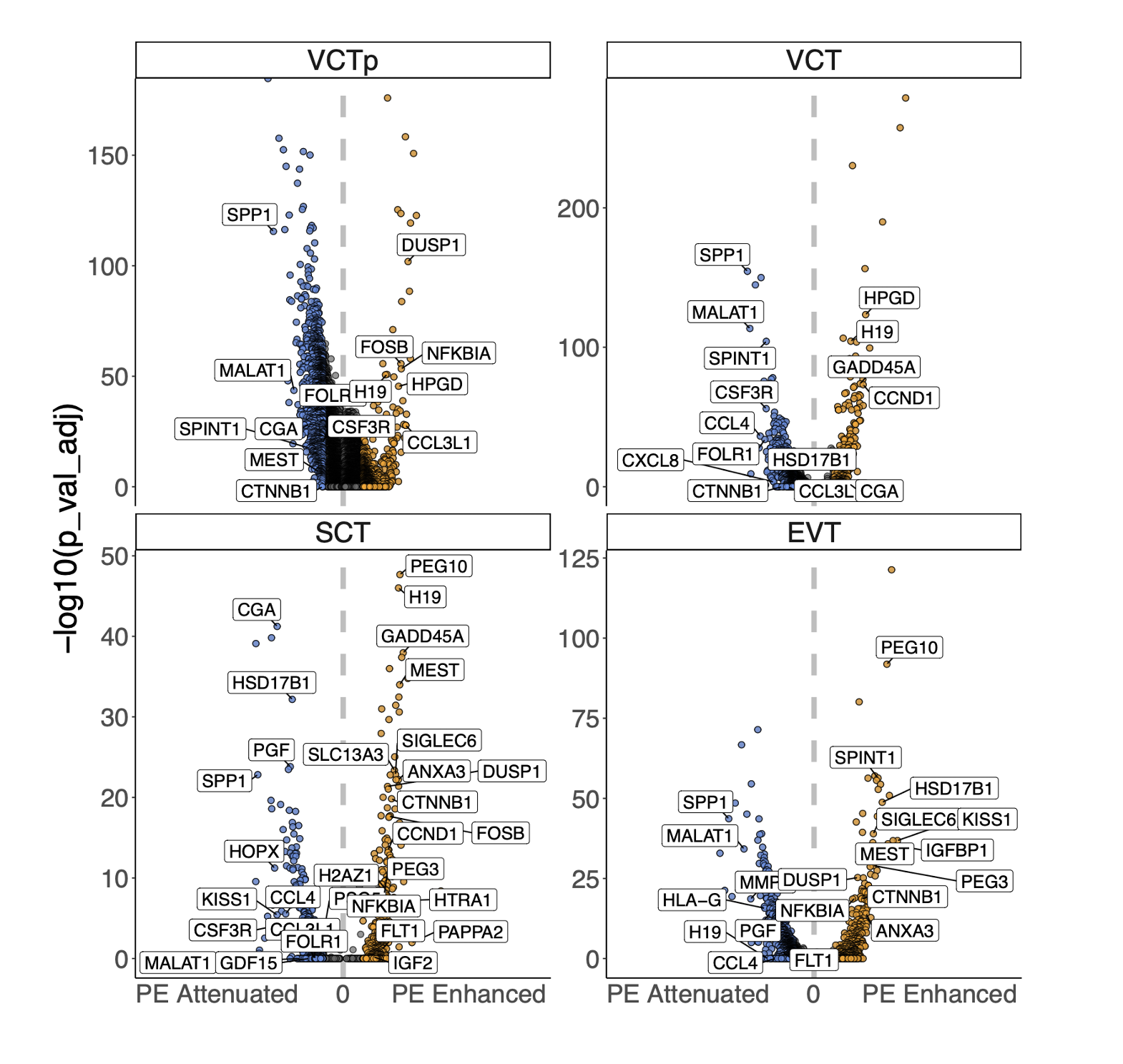


**Fig. S26. Differential gene expression in each trophoblast lineage.**

Differential gene expression between control and PE in different trophoblast lineages. Orange: genes with significantly enhanced expression in PE; blue: genes with significantly decreased expression in PE.


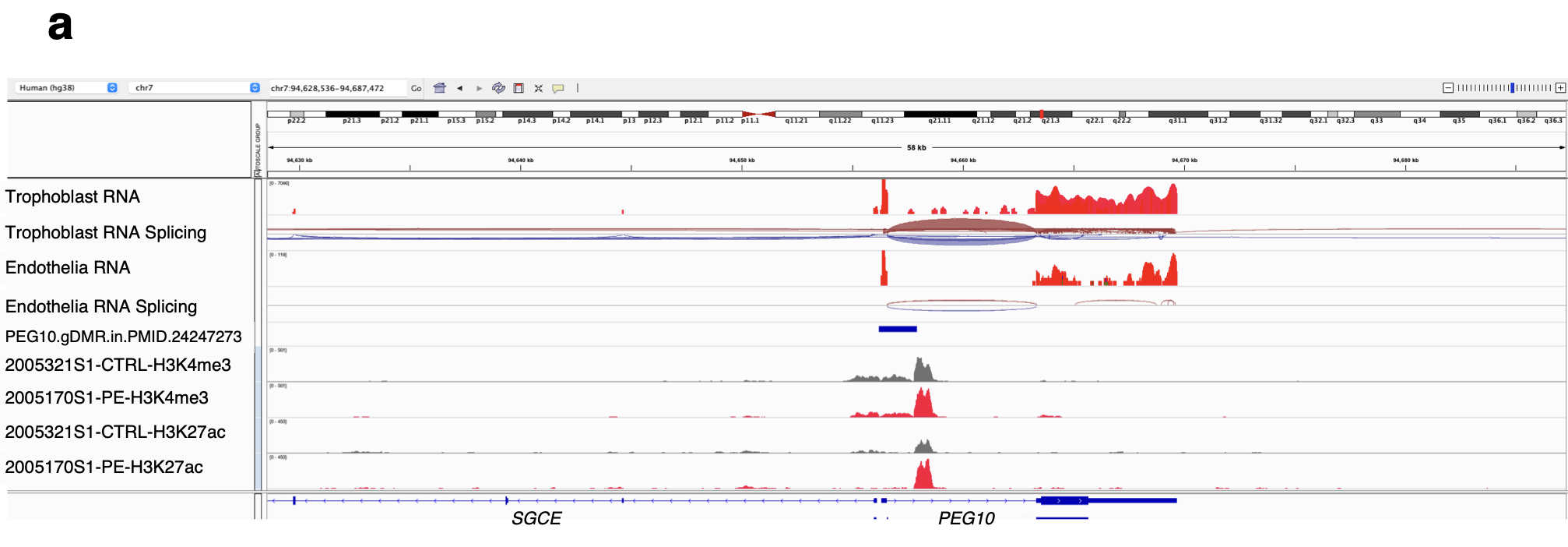


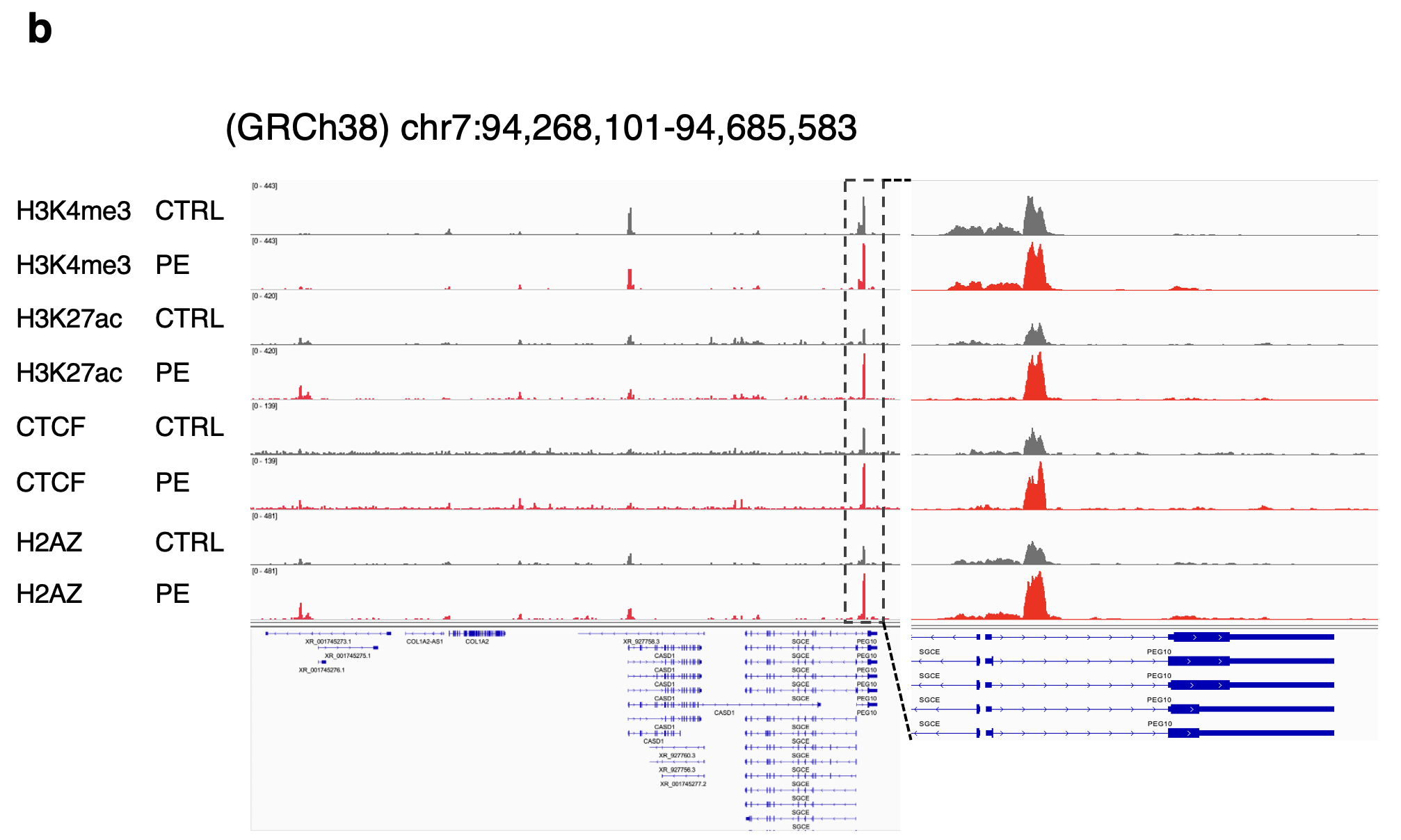


**Fig. S27. Differential epigenetic modification around *PEG10.***

**(a)** RNA structure, chromatin accessibility, enhancer/promoter location, and gDMR position around *PEG10* locus. RNA reads are shown in log scale to better visualize the low coverage regions. Histone modifications of control /PE placenta are shown in similar scale. The PEG10 gDMR defined in Hannula-Jouppi *et al*. [87]is shown for comparison. Reference genome is hg38. **(b)** Left: Histone modification (including H3K4me3, H3K27ac, CTCF and H2AZ) by Cut&Tag around *PEG10* genomic loci in control (grey) and PE (red) placenta in (GRCh38) chr7:94,268,101-94,685,583. Right: Zoom-in view around *PEG10* promoter region.

**
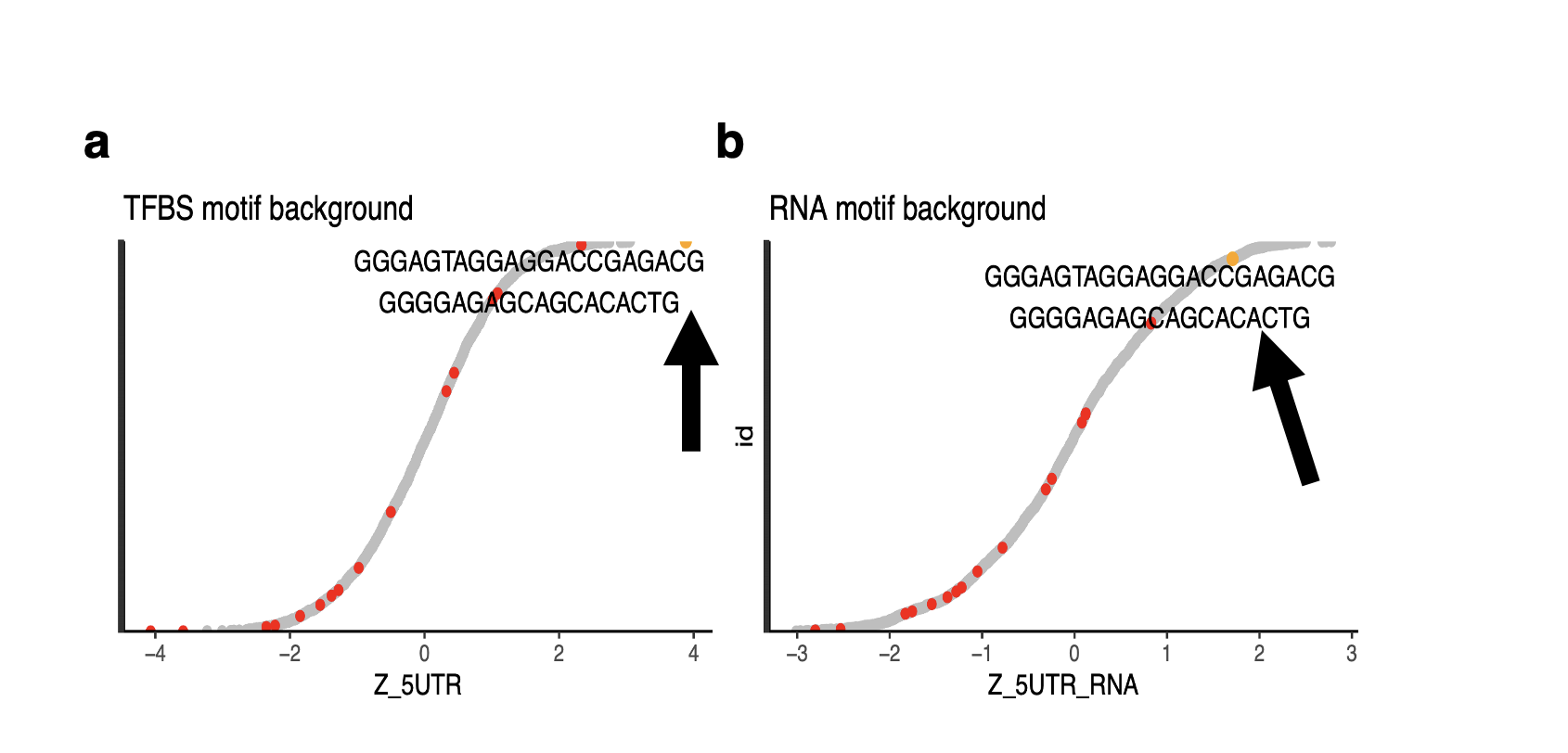
**

**Fig. S28. Motif similarity of cargo genes.**

Z-score of similarity between the *de novo* inferred sequence motif of cargo genes, and random backgrounds. Y axis denotes the rank of Z-score, and X axis is the Z-score. Gray dots are background motifs of JASPAR2015 TFBS motifs **(a)** or motifs of RNA binding proteins **(b)**. Red dots are similarities between 5’ UTR and 3’ UTR motifs. The orange dot represents 5’UTR GGGAGTAGGAGGACCGAGACG motif compared to 3’UTR GGGGAGAGCAGCACACTG motif, which is with a highest similarity amongst all comparisons (similarity = 0.68, Z as 4.3 in random TFBS background or 1.8 in RNA binding protein background).


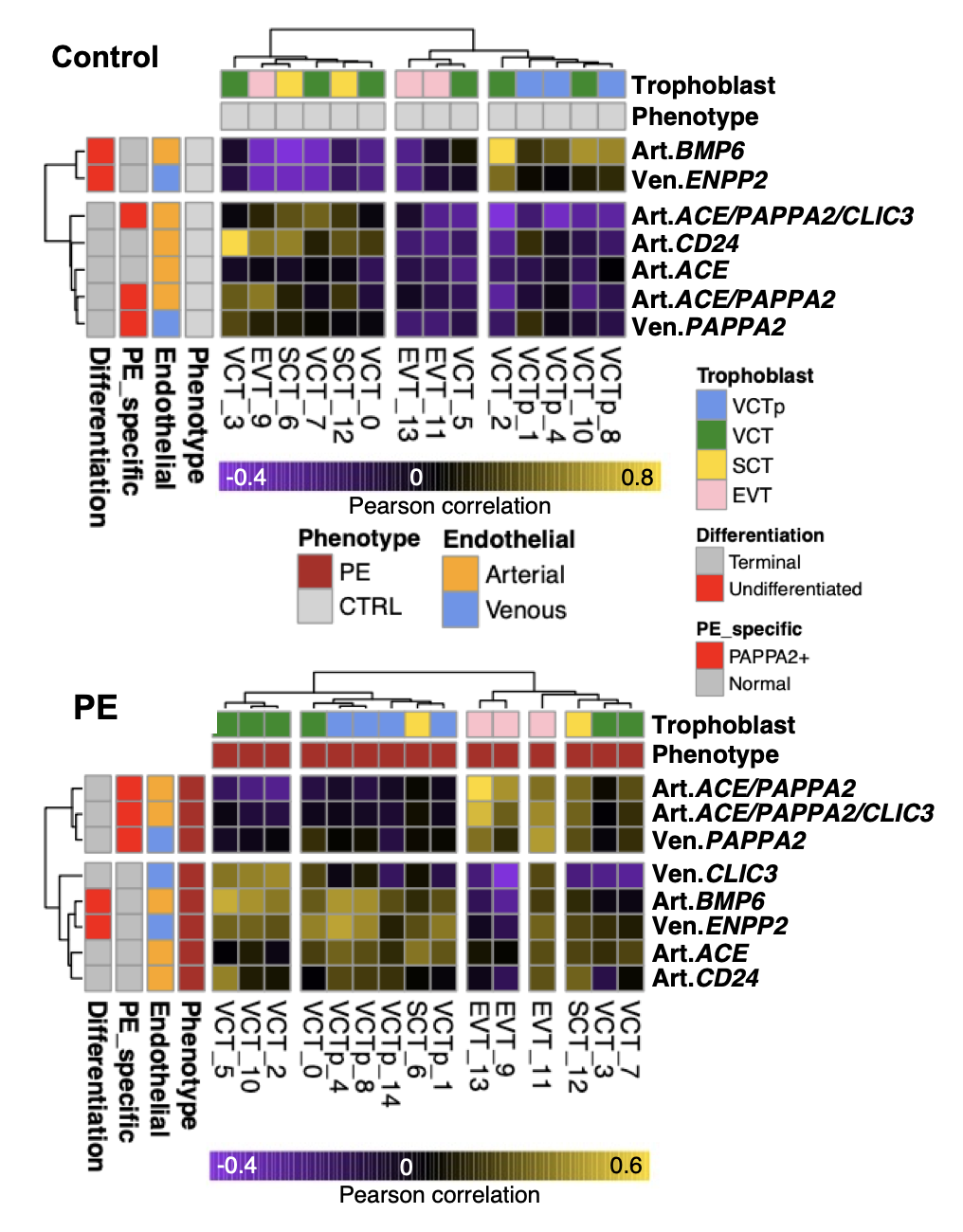


**Fig. S29. Heatmap of similarity of cargo gene RNA expression between trophoblasts and endothelial cells.**

Heatmap of Pearson's correlation of RNA expression level of putative transferred 'cargo' transcripts between trophoblasts and endothelial cells, unsupervised hierarchical clustering (trees) shows that cells in proximity of each other are more likely to share a common expression profile of these genes. The terminally differentiated SCT and EVT does not share cargo expression profile with endothelial cells in control (top panel). In PE, these cells show high degree of cargo expression profile similarity with ACE/PAPPA2+ endothelial cells (bottom panel). ***Abbreviation***: Art: arterial; Ven: venous; Art.A+P: Arterial ACE+ PAPPA2+; Art.A+P+C: Arterial ACE+ PAPPA2+ CLIC3+.


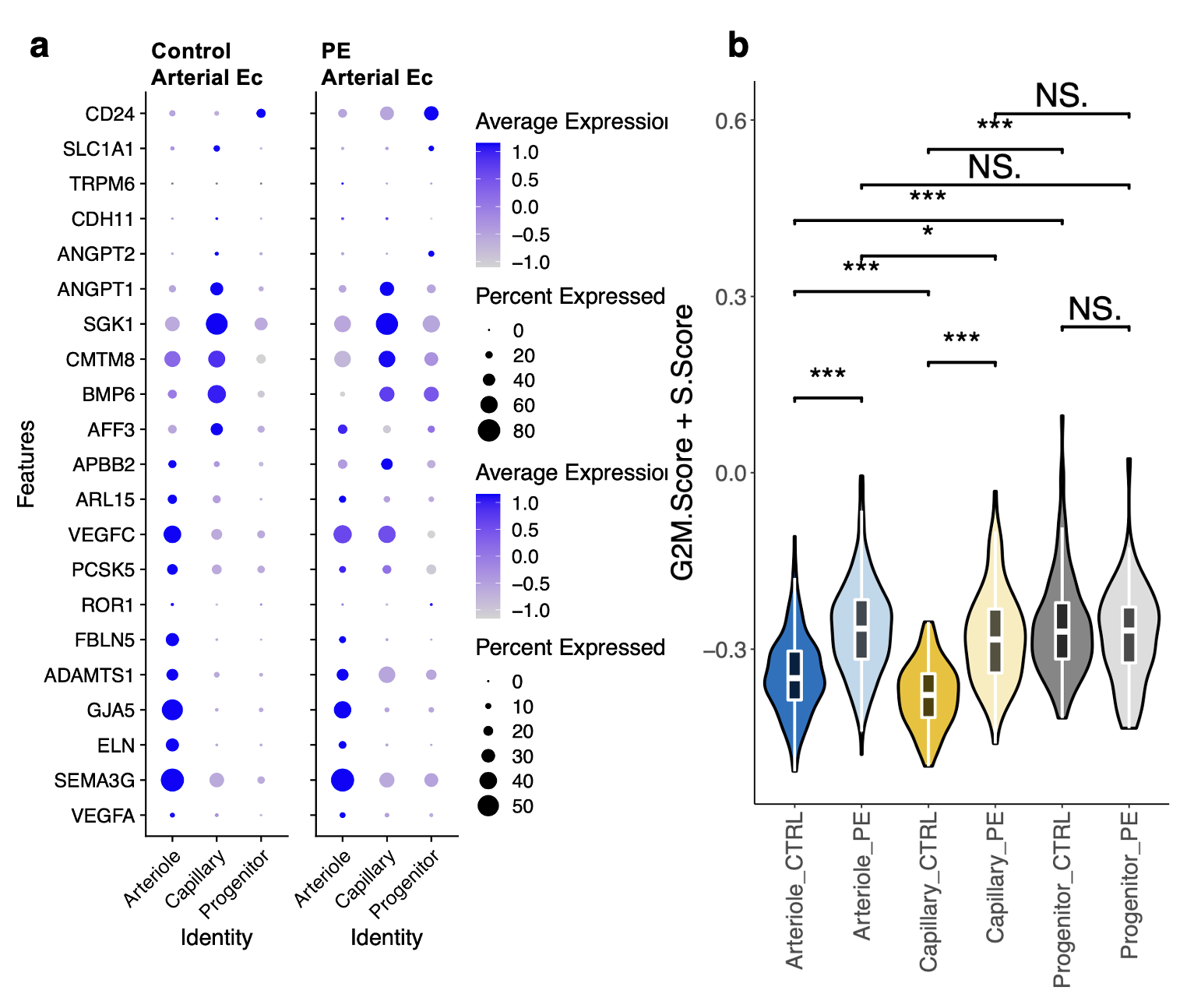


**Fig. S30. Differential expression and cell cycle activity in endothelial cells.**

**(a)** Differential expression of capillary marker (*SGK1*), arteriole marker (*SEMA3G*), angiogenic factors (*VEGFA/VEGFC/ANGPT1/ANGPT2*) and other arterial marker in control and PE arteriole clusters (ACE+ arterial clusters in Fig. 7a). **(b)** Active cell division score (inferred by G2M score + S score), in progenitor (CD24+), capillary (SGK1+) and arteriole (ACE+/SEMA3G+) between control and PE arterial cells. Progenitor clusters show higher cellular division rate than capillary and arteriole. In capillary and arteriole, PE cells divided more active than control. P-values were tested by t-test (*: P<0.05, ** P<0.01, ***: P<0.001. ns: not significant).

**
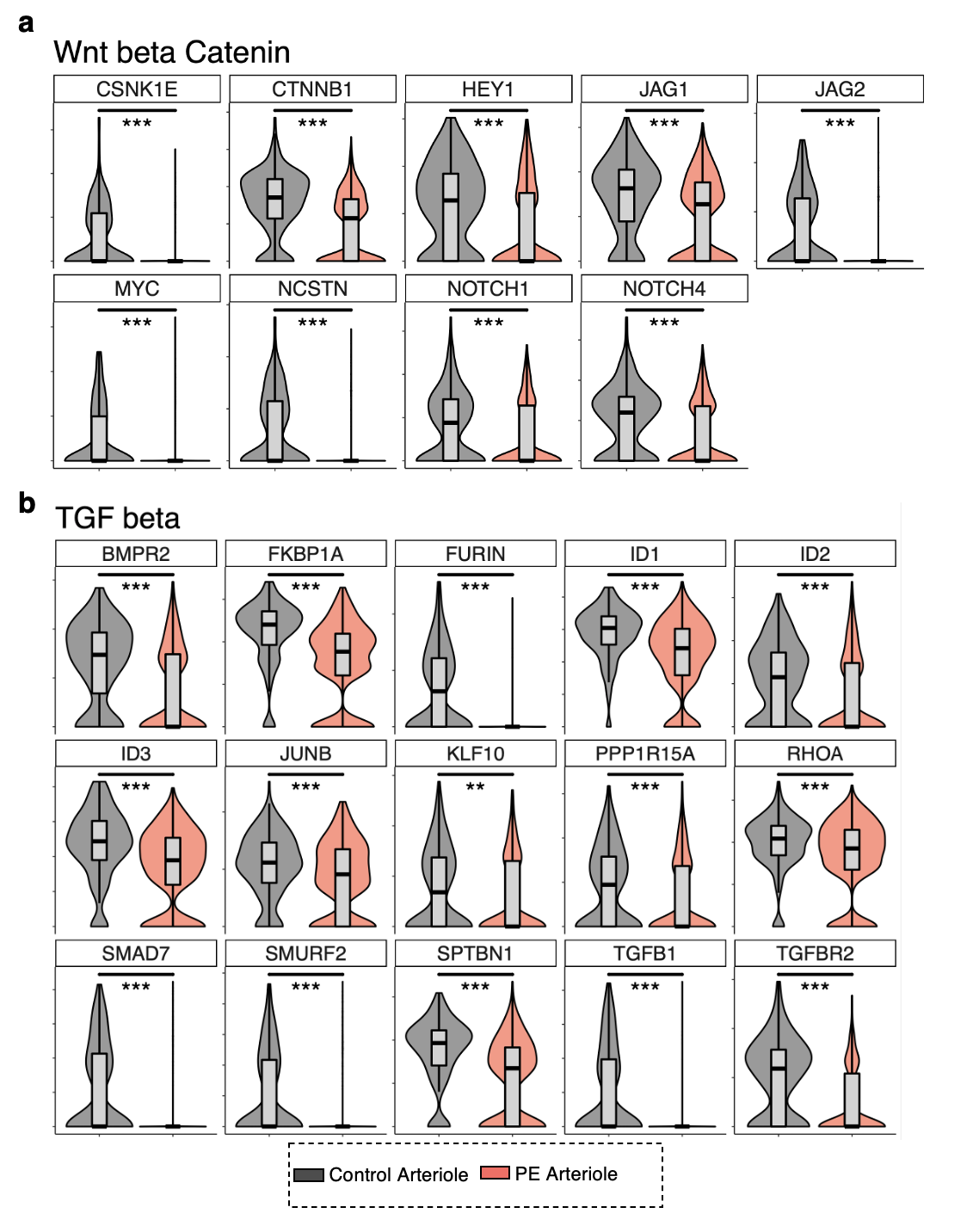
**

**Fig. S31. Differential expressed genes in Wnt beta Catenin and TGF-beta pathways.**

RNA expression of genes involved in Wnt/beta-catenin **(a)** and TGF-beta **(b)** signaling between control (grey) and PE (red) arteriole cells. P value was tested by t-test (*: P<0.05, ** P<0.01, ***: P<0.001).


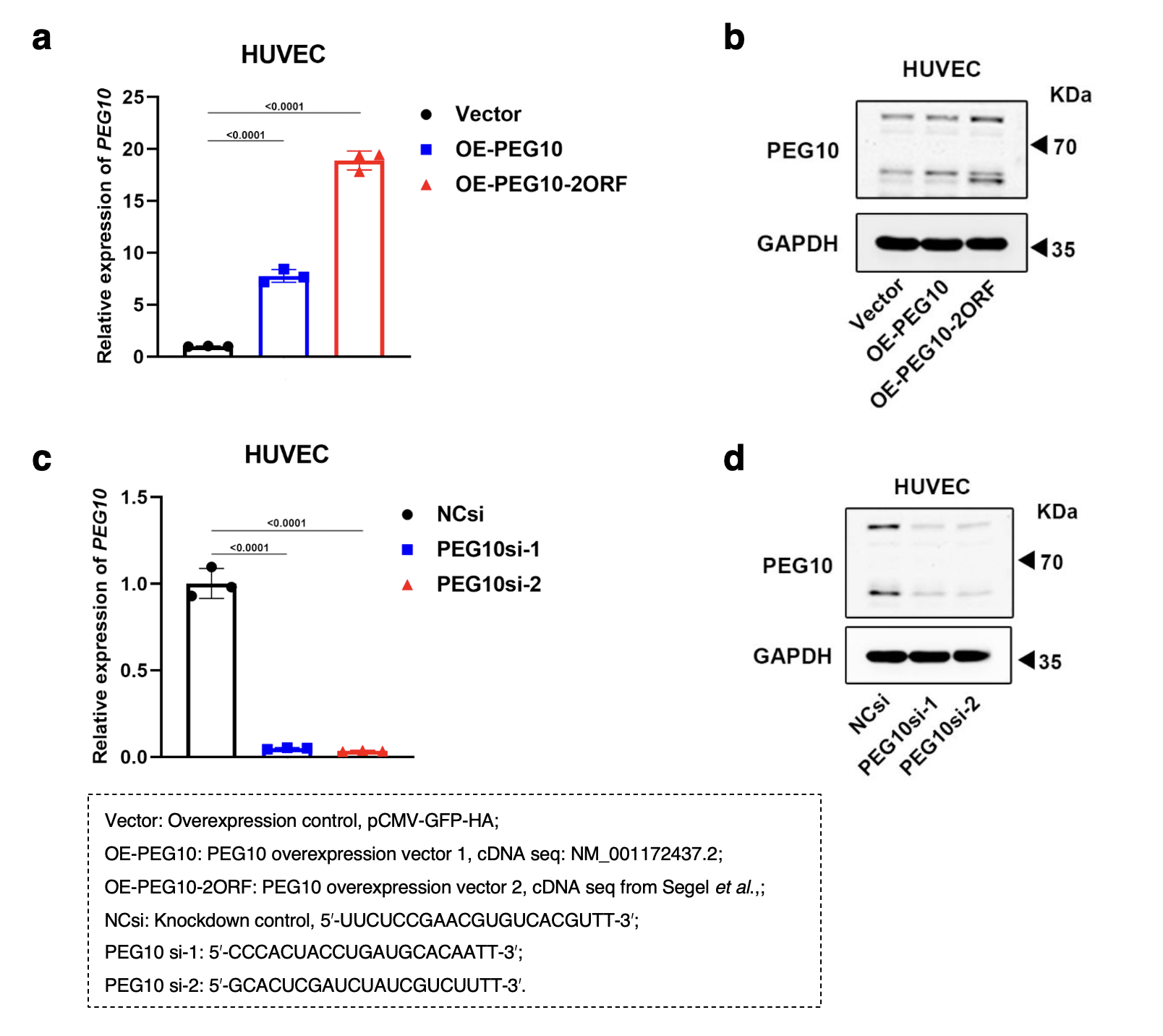


**Fig. S32. PEG10 qPCR and Western blot.**

**(a)** Relative RNA expression of PEG10 in HUVEC cells transfected with control vector (Vector) or PEG10-overexpression (OE-PEG10, OE-PEG10-2ORF) vectors. **(b)** PEG10 Western blot in HUVEC cells transfected with control vector (Vector) or PEG10-overexpression (OE-PEG10, OE-PEG10-2ORF) vectors. **(c)** Relative RNA expression of PEG10 in HUVEC cells transfected with control scrambled siRNA (NCsi) or PEG10-targeting siRNA (PEG10si-1, PEG10si-2). **(d)** PEG10 Western blot in HUVEC cells transfected with control siRNA (NCsi) or PEG10-targeting siRNA (PEG10si-1, PEG10si-2). P-values were tested by t-test.


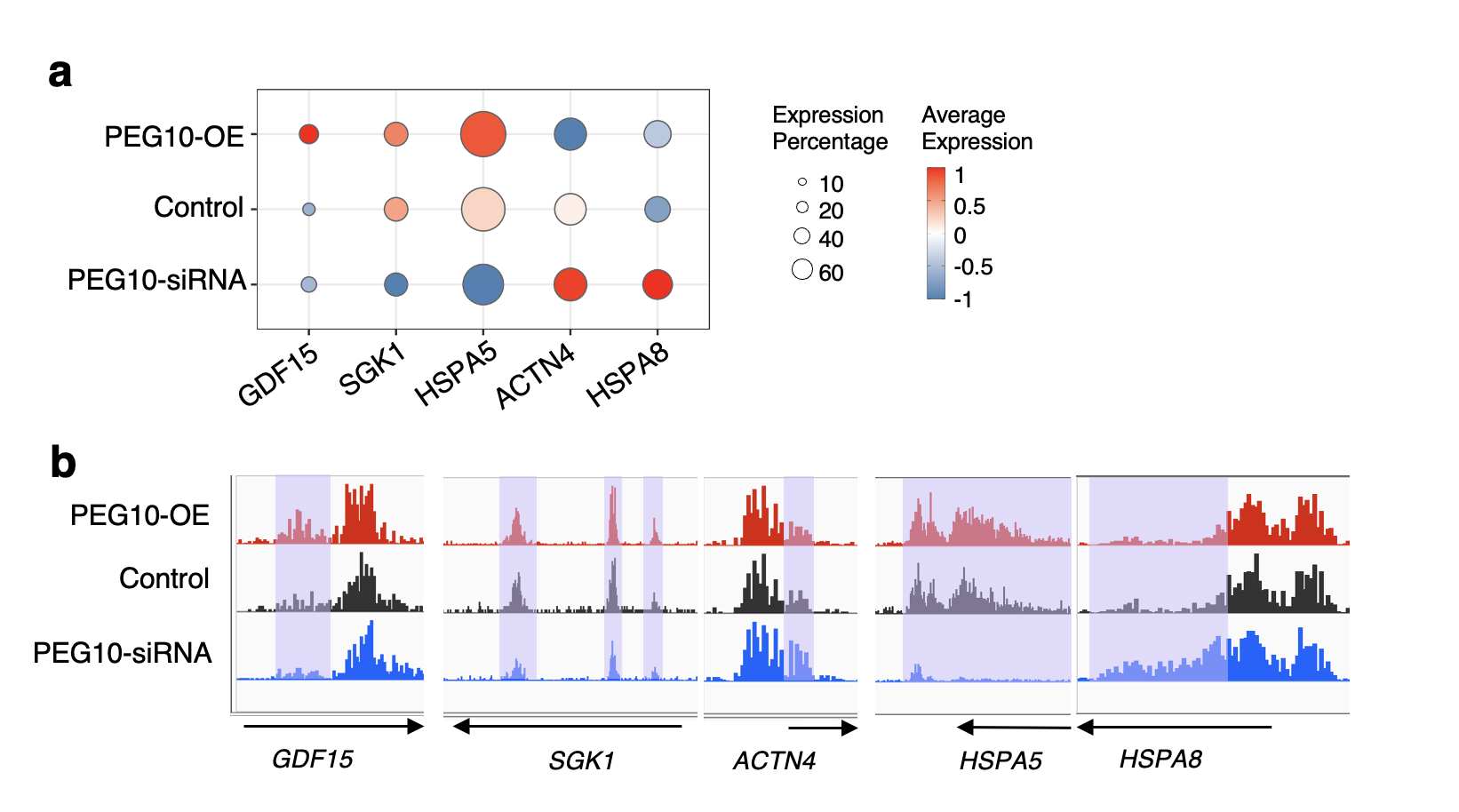


**Fig. S33** **Transcriptional dysregulations under PEG10 perturbation.**

PEG10 overexpression caused **(a)** transcriptional dysregulation and **(b)** altered chromatin accessibility of endothelial genes *SGK1* and *ACTN4*, and heat-shock response genes *HSPA8* and *HSPA5*.
