# Supplementary material for: Disruption of maternal vascular remodeling by a fetal endoretrovirus-derived gene in preeclampsia
Source: Genome Biol. 2024 May 7;25:117. doi: 10.1186/s13059-024-03265-z (PMC11075363; doi:10.1186/s13059-024-03265-z)
Supplement: Supplementary file 10 — Additional file 10. Uncropped images of Western blots in Fig. S32. [file 13059_2024_3265_MOESM10_ESM.pptx]

## Slide 1
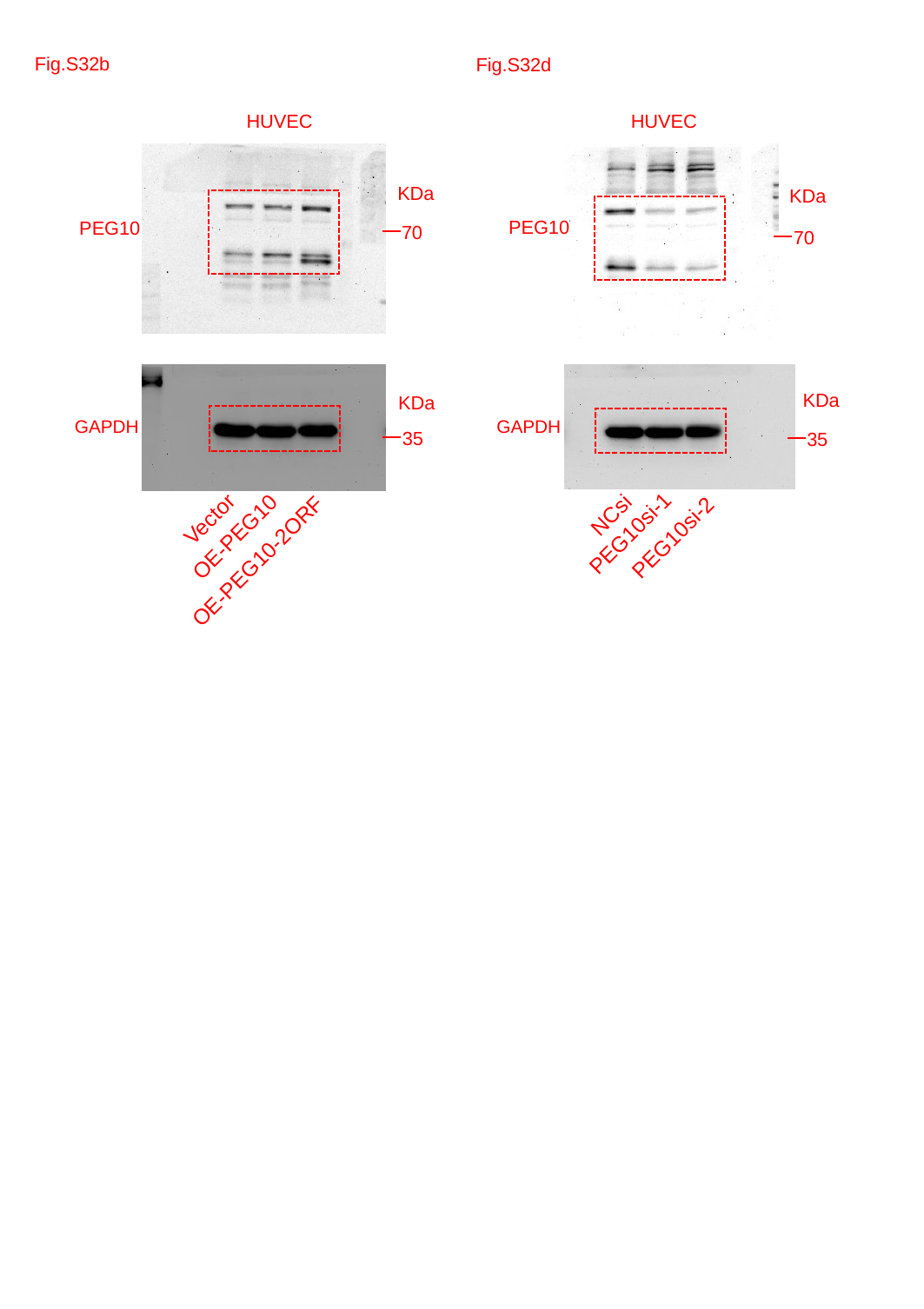

Fig.S32b
Fig.S32d
HUVEC
HUVEC
KDa
KDa
PEG10
PEG10
70
70
KDa
KDa
GAPDH
GAPDH
35
35
NCsi
Vector
PEG10si-1
OE-PEG10
PEG10si-2
OE-PEG10-2ORF
