# Supplementary material for: Disruption of maternal vascular remodeling by a fetal endoretrovirus-derived gene in preeclampsia
Source: Genome Biol. 2024 May 7;25:117. doi: 10.1186/s13059-024-03265-z (PMC11075363; doi:10.1186/s13059-024-03265-z)
Supplement: Supplementary file 11 — Additional file 11. Protocol for accessing the data with controlled access on GSA. [file 13059_2024_3265_MOESM11_ESM.docx]

To access the raw data in this study (HRA001423), navigate to the project's information page (https://ngdc.cncb.ac.cn/gsa-human/browse/HRA001423) and click ‘Request Data’ to submit the data request form, following the protocol provided by GSA (example steps are shown below). The Data Access Commity (DAC): Drs. Yuan Wei (weiyuanbysy@163.com) and Yi Zhang (zy@eulertechnology.com), will evaluate your request and feedback within one week.

The GSA data request policy could be found at: https://ngdc.cncb.ac.cn/gsa-human/document/GSA-Human_Request_Guide_for_Users_us.pdf . Any questions about the GSA site could be asked by email to gsa@big.ac.cn.
