# Supplementary material for: Disruption of maternal vascular remodeling by a fetal endoretrovirus-derived gene in preeclampsia
Source: Genome Biol. 2024 May 7;25:117. doi: 10.1186/s13059-024-03265-z (PMC11075363; doi:10.1186/s13059-024-03265-z)
Supplement: Supplementary file 12 — Additional file 12. Review history. [file 13059_2024_3265_MOESM12_ESM.docx]

Review History

**First round of review**

**Reviewer 1**

**Were you able to assess all statistics in the manuscript, including the appropriateness of statistical tests used?**

There are no statistics in the manuscript.

**Comments to author:**

In this study entitled "Disruption of maternal vascular remodelling by a fetal endoretrovirus in preeclampsia", the authors reveal that the abnormal expression of a fetal endoretrovirus is a potential factor for preeclampsia through multi-layered epigenetic mechanisms. Overall, this is a good manuscript and the results are generally interesting.

I only have the following minor points that need to be clarified before I can recommend publication of the paper:

1. Line 137-138, the authors described have more stromal cells in PE placenta. While, according to the Fig. 1d, there are more stromal cells in CTRL placenta. Please explain.

In Fig. 1, please discuss the roles of PE associated endothelial cells in PE pathogenesis.

2. In Fig. 2, Please discuss the reasons why there are the immature and mitotic trophoblasts in PE.

3. In Fig. 3d, five of six master genes include PBX1, NFYA/B/C, YY1, but no EZH2. Please explain.

4. In Fig. 4, the authors studied the DNA methylation profile in PE and CTRL placentas. And the authors described the important roles of DNA methylation and H3k27me3 in many imprinted genes. So H3K27me3 profile should be performed in this study.

5. Line 322-324, "defective fetal vascular formation (Shiura et al., 2021) loss of germline"should be "defective fetal vascular formation (Shiura et al., 2021) . Loss of germline". Please check.

6. In Fig 5, the authors highlighted PEG10. While, GADD45A is an important factor for chromatin accessibility, and CCDN1 is an important factor for cell cycle. So the authors should discuss the roles of other upregulated genes.

7. Although the authors show that PEG10 is a key potential factor for PE. The authors should perform more wet experiments to confirm whether upregulation of PEG10 result in PE.

**Reviewer 2**

**Were you able to assess all statistics in the manuscript, including the appropriateness of statistical tests used?**

No, I do not feel adequately qualified to assess the statistics.

**Comments to author:**

Gong et al. analysed multi-omics sequencing data of placenta samples from the cases of preeclampsia (PE) compared to those from normal pregnancies, and they mainly claimed the following two findings: 1) trophoblast cells, a major cell type of the placenta, are developmentally delayed (i.e. relatively immature) in PE because they are epigenetically differentially regulated, and 2) PEG10, a paternally expressed retrotransposon-derived gene, is hypomethylated and overexpressed in PE, which might disrupt maternal vascular development. They applied various computational analyses to support their claims, including Scissor (inferring phenotypes), scVelo, Slingshot (developmental lineage), Monocle3 (pseudotime analysis), SCAVENGE (linking genetic associations in specific cell types), chromVAR (linking transcription factors with chromatin accessibility), cytoTRACE (maturation and differentiation analysis), gene ontology and pathway analyses etc.

Their claims sound interesting -although the first one is not completely new (e.g. see the review paper from Pfeffer and Pearton, Reproduction 2012)- but it appears that they are not supported by enough evidence (e.g. independent validation assays and/or an in vivo model). In addition, the link between the two claims looks very weak -PEG10 seemed to gain its attention (out of many other imprinted genes) after the description of developmental delay in PE, which seems to lack its relation between the two. Also this reviewer found that the manuscript is very dense in terms of interpreting extensive amounts of their analyses as the story progresses. It could be that there are many presented figures (both in the main and the supplementary) generated by a number of different computational tools, and there are many pointers of these in the current manuscript, which make this reviewer go back and forth to follow them. Maybe it would be nice to tone down a little bit and focus on either one of their claims, probably not both, with more lines of evidence. That said, this reviewer found a similar preprint manuscript from the same research group, where they focused on the first part of their claim (i.e. trophoblast developmental delay in PE; https://www.biorxiv.org/content/10.1101/2020.05.08.085290v3.full) -strangely it was not referenced in the current manuscript. The overall tone of the current manuscript sounds very speculative and this makes it difficult to evaluate whether or not they could be reproduced. For example, how could the author validate their findings suggested from Scissor and SCAVENGE? Throughout, causal mechanisms were inferred based on computational analyses. However without experimental evidence, directionality cannot be inferred. For example, while there's interesting data showing that PEG10 is overexpressed in PE trophoblasts and likely targeted to maternal cells, it cannot be concluded that this is the cause of maternal artery maldevelopment. Finally, considering heavy use of computational analyses, the method sections are not described in detail enough to reproduce their figures and findings - the authors are encouraged to provide the codes used in their analysis. Overall, it remains unsatisfactory to warrant publication in its current form.

Specific comments:

The left-most panel of Figure 1a is misleading. The authors used the external scRNA-Seq datasets (line 116-118), but they were omitted from Figure 1a. Strangely they seemed to be included in the UMAP figure uner "Cell Phenotyping" panel, as well as Fig 1b, 1c and 1d. Also according to the "Phenotype" column in Table S1, for those samples with scRNA, there are 4 SPE samples (3 with GDM; 1 with FGR) and 1 DCDA_sIUGR_SPE. However in Figure 1a, it is shown that 6 PE samples (3 PE with GDM; 1 PE with DCDA and IUGR; 2 PE with FGR), of which the number of PE with FGR does not match. In addition, 1 CTRL sample (donor_id=2100042) and 1 sample with DCDA_sIUGR_SPE are annotated as not being used in Figure 1, which apparently conflicts with what is shown in Figure 1a. Also, line 118, CTRL should be defined where it appears for the first time. In Fig 1d, it is confusing which samples were included in the CTRL category (external scRNA datasets deemed to be included in this category). According to Table S1, CTRL includes both 'healthy' (n=1) and 'preterm' (n=3) and 'GDM (n=1)' (as shown in Fig 1a) - this should be mentioned explicitly either in the main text or in the figure legend. Fig 1h - inconsistent cell type annotation in the insets (e.g. Fibro vs Fibroblast). Inconsistent list of genes between Fig 1f and Fig 1g.

The scRNA datasets consist of external stem cells, early gestation, and preterm and term from the current study. However the external bulk RNA-Seq (from Yang et al.) used in the Scissor procedure is based on advanced gestation from the placenta which is the fetal origin. Therefore, in theory, Scissor would not show any signal of PE from non-placental tissues of scRNA-Seq. This looks a bit obvious from Fig 1h where PE-associated cells are located in the fetal origin shown in Fig 1c. Also, the authors might like to consider repeating the analysis using external scRNA datasets of PE and controls at term (and/or preterm) to see whether the patterns are reproducible?

It is not clear how possible batch effects were resolved, e.g, in-house vs external sources; samples of different locations from the current studies.

Fig 2e and Fig 2f - it would be great to add distribution curves of latent time and CytoTRACE score along with the x and y-axis. Fig 2g, as the CTRL and PE groups consist of both term and preterm delivery, this reviewer wondered whether there is any confounding effect by the status of preterm? Fig 2b and Fig 2e, the CTRL group includes first trimester and stem cells (both external datasets) as well as third trimester samples (current study) - maybe 'juvenile' and 'adult' may represent these?

Line 361, "The promoter of PEG10 is differentially methylated in the gamete germline (germline differentially methylated region: gDMR) (Acurzio et al., 2021)" and the author showed the promoter region in Fig 6a, which is in the intronic region of PEG10. However, the study from Lux et al. Plos One, 2010 (also referenced from the current study) suggested the promoter would be located in the upstream of transcript start start, rather than within the intronic region of PEG10.

What are the thresholds being used in 'PE-associated' and "CTRL-associated' cells?

SCT and Endo from Fig S1c do not seem to agree with Fig S4b.

Line 1242, maybe better to replace "differential genes" with "differentially expressed genes". Also, "pvalue < 0.05" - does it mean the raw p-values or adjusted p-value? "Absolute value of log2FC > 0.5" - does it mean the original (i.e. unbroken) log2FC or shrunken log2FC?

It would be great to include the QC metrics for all the sequencing data, e.g. sequencing depth, the number of filtered reads etc.

The selection of their references should be trimmed and they should be more focused. Likewise, the discussion section is too long.

Please provide links of e GSA repository (Accession number: HRA001423)

In Fig S1a - the color code for the data from Yu et al is not distinguishable.

Fig S3a the cell type annotation of scATAC has been covered by that of scRNA

Very difficult to follow the main figures and their related supplementary figures. For example, page 8, Figure S7, a supplementary figure for Fig 2, is mentioned before Figure S5 and S6.

Fig S6d and S6e, no legend for DC1 and DC2.

Line 859, no reference given to ACOG guideline.

Page 35, "Public data" - please give the accession numbers of external datasets.

Line 909, 915, 962, please provide the model number of Novaseq and the place where it was performed.

Line 984, which normalisation method was used? And line 1040, which adjustment method was used?

Line 1017, no reference given to cisTopic and line 1229, no reference given to eagle.

Line 1047, "pct.1-pct.2" what does that mean?

Page 46, bulk RNA-Seq data processing is not described in detail. For example, no descriptions of the options used by STAR for mapping and quantification. Also give a full reference for GSE148241. It is very unusual not doing multiple corrections for p-values.

In Fig 4g and Fig 5a, is the y-axis based on adjusted or unadjusted p-values?

Fig S17 - neither coordinates nor the transcript architecture provided for PEG10.

**Authors Response**

**Point-by-point responses to the reviewers’ comments:**

Reviewer #1:

In this study entitled "Disruption of maternal vascular remodelling by a fetal endoretrovirus in preeclampsia", the authors reveal that the abnormal expression of a fetal endoretrovirus is a potential factor for preeclampsia through multi-layered epigenetic mechanisms. Overall, this is a good manuscript and the results are generally interesting.

*Reply: We thank the reviewer for reviewing our paper and giving many important suggestions. We thank the reviewer to find our manuscript 'a good manuscript and the results are generally interesting'. Particularly, we thank the reviewer asking us to perform additional wet experiments to prove the function of PEG10 on endothelial cells, and to analyze H3K27me3 modification in PE and CTRL placenta. We have now performed the experiments par the reviewer's request.*

I only have the following minor points that need to be clarified before I can recommend publication of the paper:

1. Line 137-138, the authors described have more stromal cells in PE placenta. While, according to the Fig. 1d, there are more stromal cells in CTRL placenta. Please explain.

*Re: We thank the reviewer for the question. Measuring the space occupied by 'CTRL stromal cells' in Fig. 1d might be misleading. The frequency of stromal cell ("Endo" and "Fibro") in PE and CTRL placenta samples were shown in Supp.Fig. 1d, (please refer to Fig. R1 in the ‘Response letter’ pdf file). We apologize for the confusion.*

In Fig. 1, please discuss the roles of PE associated endothelial cells in PE pathogenesis.

*Reply: We thank the reviewer for the suggestion. We have now added a brief discussion on PE associated endothelial cells in the results section accompanying Fig. 1, as follow:*

*" Trophoblast dysfunction in PE is well documented. On the other hand, endothelial cells might also play an important role in PE. Blood vessel defect is thought to be central for many PE phenotypes including defective placentation, hypertension, and kidney defect. During placenta implantation, the uterine spiral artery is extensively transformed. Specifically, vascular smooth muscle cells around the spiral artery degenerate, and trophoblasts invade into the vascular wall, inducing repetitive damage and repair of the endothelium cell layer. The result is a new endothelium layer generated by repetitive neo-genesis of endothelial cells. Though the endothelial cell defect in PE is documented, its underlying molecular mechanism is not yet known. Our results now provided genetic evidence to support endothelial cell to involve in PE pathogenesis." (line 165).*

2. In Fig. 2, Please discuss the reasons why there are the immature and mitotic trophoblasts in PE.

*Reply: We thank the reviewer for the suggestion. We have now added a brief discussion on PE associated immature and mitotic trophoblast cells in the results section accompanying Fig. 2, as follow:*

*"PE is known to be associated with underdeveloped placenta. Specifically, excessive immature and proliferating trophoblasts has been documented for PE placenta [1]. Our single cell analysis result confirmed this pathological observation. In PE, the delayed development of cells in trophoblast lineage possibly resulted from delayed maturation, slowed cell division, or both. Our results suggest that whilst mitotic division rate is likely to be slowed down in PE trophoblasts (Fig. 2d), delayed maturation (loss of stemness / increased differentiation) of PE trophoblasts is observed only in the terminally differentiated SCT and EVT cells (Fig. 2c). Hence, it is more likely that trophoblast development is blocked at the transition point for terminal differentiation in PE. Such phenotype could be resulted from incomplete epigenetic reprogramming." (line 225).*

3. In Fig. 3d, five of six master genes include PBX1, NFYA/B/C, YY1, but no EZH2. Please explain.

*Reply: We thank the reviewer for the question. EZH2 binding site is not present in the JASPAR and CISBP database we used for ArchR-mediated inference of TFBS activity. The re-analysis including manually selected TFBS motif file into the full refference database is technically difficult. Hence, the analysis did not include EZH2. However, YY1 recruits EZH2 to many of its binding sites [2], and we consider the reduced YY1 activity as inferred in Fig. 3d is strongly linked to EZH2 reduction. Furthermore, the transcription factors E2F7/E2F4 that promoting EZH2 transcription (https://maayanlab.cloud/archs4/gene/EZH2#correlation) are downregulated in PE (please refer to Fig. R2 in the ‘Response letter’ pdf file). Hence, we consider EZH2 activity is also downregulated in PE. In addition, we have now provided evidences that EZH2 binding sites were subjected to decreased H3K27me3 modification (please refer to Fig. R3 in the ‘Response letter’ pdf file).*

4. In Fig. 4, the authors studied the DNA methylation profile in PE and CTRL placentas. And the authors described the important roles of DNA methylation and H3k27me3 in many imprinted genes. So H3K27me3 profile should be performed in this study.

*Reply: We thank the reviewer for the suggestion. We have followed the reviewer's instruction, and performed H3K27me3 CUT&Tag experiment on PE and CTRL placentas. Briefly, placenta samples of CTRL and PE are subjected to CUT&Tag experiment using anti-H3K27me3 (abcam Ab6002) following the same protocol as described in the Methods section. H3K27me3 regions were called using MACS2 using the '--broad --broad-cutoff 0.1 --nolambda --SPMR --nomodel -B) option. The called H3K27me3 regions then merged to neighboring regions within +/- 5kbp to define H3K27me3 domains. Differentially modified region were called as abs(mean(PE)-mean(CTRL))/(std(PE)+std(CTRL)) > 2. Enrichment of EZH2-bound regions for H3K27me3 gain or loss in PE were done with regioneR::permutationTest. Overall profile of H3K27me3 on EZH2-bound-regions were computed by deepTools.*

*In total, we identified 45,693 regions that differentially enriched for H3K27me3 betwen PE and CTRL placentas. In these regions, 32,409 regions showed increased H3K27me3 signal in PE, and 13,284 regions showed decreased H3K27me3 signal in PE (Fig. R3a, R3b). For example, H3K27me3 enrichment around a strongly repressed promoter of PAPPA is lost in PE placenta compared to CTRL (Fig. R3b). Overall, H3K27me3 signal are lost in PE placenta around +/- 2kbp within EZH2 binding sites (Fig. R3c). Significant enrichment of H3K27me3 loss was found on EZH2 binding sites (Fig. R3d).*

*The differential H3K27me3 modification on PE placenta is likely to affect gene imprinting status. We thus analyzed H3K27me3 modification on imprinting genes. H3K27me3 modification is increased on known maternal imprinted genes (expressed allele is paternal) compared to paternally imprinted genes (expressed allele is maternal) (please refer to Fig. R4 in the ‘Response letter’ pdf file). Combined with the RNA expression (Fig. 4h, 4i) and DNA methylation (Fig. 4f), these data suggest that epigenetic reprogramming is disrupted in PE placenta, leading to dysregulation of imprinted genes.*

5. Line 322-324, "defective fetal vascular formation (Shiura et al., 2021) loss of germline"should be "defective fetal vascular formation (Shiura et al., 2021) . Loss of germline". Please check.

*Reply: We thank the reviewer for pointing out this mistake. We have followed the reviewer's instruction to modify this typo.*

6. In Fig 5, the authors highlighted PEG10. While, GADD45A is an important factor for chromatin accessibility, and CCDN1 is an important factor for cell cycle. So the authors should discuss the roles of other upregulated genes.

*Reply: We thank the reviewer for the suggestion. We have followed the reviewer's instruction to analyze and discuss the role of other upregulated genes in PE. We performed GSEA enrichment of differentially expressed genes in the PE trophoblast scRNA dataset (please refer to Fig. R5 in the ‘Response letter’ pdf file). PE trophoblasts showed increased gene set activities in 'G2M checkpoint', 'E2F targets', and 'MYC targets' pathways. These results is in concordance to the function of PE upregulated genes such as CCND1 (an important factor that regulates cell cycle) and GADD45A (an important factor that regulates chromatin accessibility and cell cycle). The results suggest a scenario that in PE trophoblast, GADD45A expression is induced to arrest the cells at G2/M checkpoint from further division and differentiation, which is supported by cell-cycle gene set expression scoring on single cell RNA-seq dataset (Fig. R5b).*

*These results are now put into the results and discussion sections.*

7. Although the authors show that PEG10 is a key potential factor for PE. The authors should perform more wet experiments to confirm whether upregulation of PEG10 result in PE.

*Reply: We thank the reviewer for the suggestion. We have followed the reviewer's instruction, and performed wet experiments to show that PEG10 is essential for vascular endothelial cell development and function.*

*We performed siRNA knockdown (2 different siRNAs) or cDNA overexpression (2 different constructs) of PEG10 in human endothelial cell line (HUVEC). The manipulations changed the PEG10 RNA expression (please refer to Fig. R6 in the ‘Response letter’ pdf file) and protein expression level (Fig. R6b and R6d) relative to the basal level.*

*Compared to control experiments (scrambled siRNA, or empty overexpression construct), PEG10 overexpression induced growth suppression of endothelial cells, whilst PEG10 knockdown induced excessive cell growth (please refer to Fig. R7 in the ‘Response letter’ pdf file). In addition to this, we have also performed ShareSeq (scRNA and scATAC assay on the same single cell) experiment on PEG10 overexpressed, PEG10 knockdown, and CTRL cells. Cell cycle scores of scRNA expression clearly showed that PEG10 expression has an negative effect on cell cycle (Fig. R7b). Hence, PEG10 inhibits endothelial cell growth.*

*We then tested whether endothelial cell functionality is disrupted by PEG10 with in vitro tube formation assay. The results showed that PEG10 overexpression results in reduction of tube counts, and a reduction of tube junctions (Fig. R7c-R7d). On the contrary, PEG10 siRNA increased both tube counts and junction counts compared to scrambled siRNA control (Fig. R7e-R7f). Hence, PEG10 disturbed endothelial cell function to inhibit endothelial tube formation.*

*Compared to PEG10 overexpressed cells, PEG10 knockdown cells showed elevated expression of proliferation-related genes such as MKI67 and TOP2A, as well as a number of TGF-beta signalling pathway molecules such as SMAD3, TGFB1 and TGFBR2 (please refer to Fig. R8 in the ‘Response letter’ pdf file), whilst PEG10 overexpression resulted in increased expression of cell cycle arrest genes such as GADD45A (Fig. R8a). Gene set expression analysis also identified cell cycle/proliferation pathways as downregulated in PEG10 overexpressed cells compared to PEG10 RNAi cells (Fig. R8b). PEG10 perturbs TGF-beta signalling via directly binding to ACVRL1[3]. Angiogenesis-related gene sets including 'Hallmark of angiogenesis' and 'Angiotensin II receptor type 1 pathway' are negatively regulated by PEG10 (Fig. R8b). Supplementing TGFB1 protein in the culture medium rescued PEG10 overexpression effect in endothelial tube formation assay (Fig. R8c-R8d). Hence, PEG10 directly perturbs endothelial cell function in PE by down-regulating TGF-beta signalling. Finally, PEG10 overexpression affects the transcription of many genes essential for angiogenesis and endothelial function, such as SGK1, GDF15, and ACTN4 (please refer to Fig. R9 in the ‘Response letter’ pdf file), suggesting possible downstream effector of PEG10-TGF-beta signalling.*

*References for Reviewer 1*

*1. Redline RW, Patterson P: Pre-eclampsia is associated with an excess of proliferative immature intermediate trophoblast. Hum Pathol 1995, 26:594-600.*

*2. Caretti G, Di Padova M, Micales B, Lyons GE, Sartorelli V: The Polycomb Ezh2 methyltransferase regulates muscle gene expression and skeletal muscle differentiation. Genes Dev 2004, 18:2627-2638.*

*3. Lux A, Beil C, Majety M, Barron S, Gallione CJ, Kuhn HM, Berg JN, Kioschis P, Marchuk DA, Hafner M: Human retroviral gag- and gag-pol-like proteins interact with the transforming growth factor-beta receptor activin receptor-like kinase 1. J Biol Chem 2005, 280:8482-8493.*

Reviewer #2:

Overall comments:

Gong et al. analysed multi-omics sequencing data of placenta samples from the cases of preeclampsia (PE) compared to those from normal pregnancies, and they mainly claimed the following two findings: 1) trophoblast cells, a major cell type of the placenta, are developmentally delayed (i.e. relatively immature) in PE because they are epigenetically differentially regulated, and 2) PEG10, a paternally expressed retrotransposon-derived gene, is hypomethylated and overexpressed in PE, which might disrupt maternal vascular development. They applied various computational analyses to support their claims, including Scissor (inferring phenotypes), scVelo, Slingshot (developmental lineage), Monocle3 (pseudotime analysis), SCAVENGE (linking genetic associations in specific cell types), chromVAR (linking transcription factors with chromatin accessibility), cytoTRACE (maturation and differentiation analysis), gene ontology and pathway analyses etc.

Their claims sound interesting -although the first one is not completely new (e.g. see the review paper from Pfeffer and Pearton, Reproduction 2012)- but it appears that they are not supported by enough evidence (e.g. independent validation assays and/or an in vivo model). In addition, the link between the two claims looks very weak -PEG10 seemed to gain its attention (out of many other imprinted genes) after the description of developmental delay in PE, which seems to lack its relation between the two. Also this reviewer found that the manuscript is very dense in terms of interpreting extensive amounts of their analyses as the story progresses. It could be that there are many presented figures (both in the main and the supplementary) generated by a number of different computational tools, and there are many pointers of these in the current manuscript, which make this reviewer go back and forth to follow them. Maybe it would be nice to tone down a little bit and focus on either one of their claims, probably not both, with more lines of evidence.

*Reply: We thank the reviewer for reviewing our paper and giving many important suggestions. We have completely re-written the manuscript, and re-ordered the figure/tables, in order to fix the logic flow to focus on the main findings of this study. Furthermore, we have performed additional wet experiments and computational analysis to strengthen our points.*

That said, this reviewer found a similar preprint manuscript from the same research group, where they focused on the first part of their claim (i.e. trophoblast developmental delay in PE; https://www.biorxiv.org/content/10.1101/2020.05.08.085290v3.full) -strangely it was not referenced in the current manuscript.

*Reply: We thank the reviewer for the question. We have decided that the previous analysis based on bulk ATAC and DNAm sequencing is not themselves sufficient to support the hypothesis. We thus performed additional single cell sequencing experiments and re-analyzed the data to validate our hypothesis.*

The overall tone of the current manuscript sounds very speculative and this makes it difficult to evaluate whether or not they could be reproduced.

For example, how could the author validate their findings suggested from Scissor and SCAVENGE?

*Reply: We thank the reviewer for the question. We have performed wet experiments to show that PE-upregulated PEG10 negatively affects endothelial cell function by down-regulating TGF-beta signalling (please refer to Fig. R6, R7, R8, R9 in the ‘Response letter’ pdf file), validating our hypothesis. We have also validated the trophoblast developmental delay in PE in additional dataset generated by other groups (please refer to Fig. R14, R15, R16 in the ‘Response letter’ pdf file). These results further validated our computational analysis.*

Throughout, causal mechanisms were inferred based on computational analyses. However without experimental evidence, directionality cannot be inferred. For example, while there's interesting data showing that PEG10 is overexpressed in PE trophoblasts and likely targeted to maternal cells, it cannot be concluded that this is the cause of maternal artery maldevelopment.

*Reply: We thank the reviewer for the question. We have performed wet experiments to show that PE-upregulated PEG10 negatively affects endothelial cell function by down-regulating TGF-beta signalling (Fig. R6, R7, R8, R9). Particularly, we have found that endothelial cell development and function are negatively affected by overexpressed PEG10. This result, in agreement to the previously reported widespread endothelial cell dysfunction in preeclampsia [1], revealed a novel molecular mechanism by which the fetal trophoblast signal to maternal endothelial cells to reprogram their fate.*

Finally, considering heavy use of computational analyses, the method sections are not described in detail enough to reproduce their figures and findings - the authors are encouraged to provide the codes used in their analysis.

*Reply: We thank the reviewer for the question. We have provided the codes and source data necessary for generating the figures in this paper, which are publicly accessible. Specifically, the source codes are placed in:*

*https://github.com/MagpiePKU/Preeclampsia_Paper_2023 (github)*

*And source data are placed in:*

*https://zenodo.org/record/8079129 (zenodo)*

Specific comments:

The left-most panel of Figure 1a is misleading. The authors used the external scRNA-Seq datasets (line 116-118), but they were omitted from Figure 1a. Strangely they seemed to be included in the UMAP figure uner "Cell Phenotyping" panel, as well as Fig 1b, 1c and 1d.

*Reply: We thank the reviewer for the question. We have added back the external scRNA-seq datasets in this schematic figure. Please refer to Fig. R10 in the ‘Response letter’ pdf file.*

Also according to the "Phenotype" column in Table S1, for those samples with scRNA, there are 4 SPE samples (3 with GDM; 1 with FGR) and 1 DCDA_sIUGR_SPE. However in Figure 1a, it is shown that 6 PE samples (3 PE with GDM; 1 PE with DCDA and IUGR; 2 PE with FGR), of which the number of PE with FGR does not match. In addition, 1 CTRL sample (donor_id=2100042) and 1 sample with DCDA_sIUGR_SPE are annotated as not being used in Figure 1, which apparently conflicts with what is shown in Figure 1a.

*Reply: We thank the reviewer for the question. We have collected in total 21 placenta samples from 11 donors for this study. In these donors, 5 were non-PE, and 6 were PE. Due to sample variability, some of the samples were only sequenced by either scRNA-seq or scATAC-seq, but not both of them. In the PE donors, 3 of them were sequenced by both scRNA and scATAC. One (1) donor was only sequenced by scATAC. Two (2) donors, including the DCDA-sIUGR donor, were only sequenced by scRNA.*

*Two (2) of these donors, a CTRL donor (2100042) and a DCDA-sIUGR donor (2200314), were not enrolled in the first study period (before 2021) but enrolled latter (after 2021) for internal validation. Hence, in Fig.1b-h, we only used 9 donors out of 11. However, these donors were also included in the Fig. 1a schematic figure to show the overall study design. We apologize for the confusion and have made it clear in the manuscript.*

Also, line 118, CTRL should be defined where it appears for the first time.

*Reply: We thank the reviewer for pointing this out. We have correspondingly added the definition of CTRL (control) at its first appearance in the manuscript. For other abbreviations, we have made sure that they were defined at their first appearance in the manuscript.*

In Fig 1d, it is confusing which samples were included in the CTRL category (external scRNA datasets deemed to be included in this category).

*Reply: We thank the reviewer for pointing this out. We have now clarified that the two external datasets of 1st trimester placentas were defined as CTRL in the manuscript.*

According to Table S1, CTRL includes both 'healthy' (n=1) and 'preterm' (n=3) and 'GDM (n=1)' (as shown in Fig 1a) - this should be mentioned explicitly either in the main text or in the figure legend.

*Reply: We thank the reviewer for pointing this out. We have now clarified the definition of CTRL as 'non-PE placenta', and explicitly mentioned that these includes 'healthy' (n=1), 'preterm' (n=3), GDM (n=1), and 1st trimester placentas from external data (n=2). These modifications were done for the main text.*

Fig 1h - inconsistent cell type annotation in the insets (e.g. Fibro vs Fibroblast).

*Reply: We thank the reviewer for pointing this out. We have now corrected the label in Figure 1h as suggested by the reviewer (please refer to Fig. R11 in the ‘Response letter’ pdf file).*

Inconsistent list of genes between Fig 1f and Fig 1g.

*Reply: We thank the reviewer for the question. The cell type definition level in scATAC and scRNA assays shown in Fig. 1b and Fig. 1e are not similar, due to visual display clarity. The markers shown in Fig. 1f and Fig. 1g followed the definition in Fig. 1b and Fig. 1e, respectively. For scRNA, we did not separately analyze CD4 and CD8 T cells in Fig. 1b. Hence, we did not show IL7R and GZMB gene* expression in *Figure 1f. Similarly, the scATAC NK cluster (too small) was not shown in Fig. 1e, and hence its marker XCL2 was not shown in Fig. 1g. Finally, the scATAC myeloid cells were not separated into M2/Monocyte/HB in Fig. 1g, and hence HBEGF/CSF1R were not shown in Fig. 1g.*

The scRNA datasets consist of external stem cells, early gestation, and preterm and term from the current study. However the external bulk RNA-Seq (from Yang et al.) used in the Scissor procedure is based on advanced gestation from the placenta which is the fetal origin. Therefore, in theory, Scissor would not show any signal of PE from non-placental tissues of scRNA-Seq. This looks a bit obvious from Fig 1h where PE-associated cells are located in the fetal origin shown in Fig 1c.

*Reply: We thank the reviewer for the question. The Yang et al bulk RNA-seq data [2] were from placenta samples without explicit mentioning of sampling procedure. However, based on the gene expression, we conclude that these samples unlikely to consist 100% of fetal origin cells. For example, the canonical endothelial cell marker PLVAP, CLDN5, CDH5 [3], as well as fibroblast marker MYL9, were found to be expressed in these samples (please refer to Fig. R12 in the ‘Response letter’ pdf file). We conclude that it is likely to have PE-associated non-fetal signal in these datasets. Furthermore, even if the Yang et al dataset is indeed enriched for cells of fetal origin, it would only affect the sensitivity instead of specificity of Scissor analysis. Hence, a positive signal in Scissor analysis is unlikely to be false-positive.*

*In Fig. 1h, the most significant scRNA PE-associated cells (x-axis) include extravillous trophoblast (EVT) and endothelial cells. Whilst EVT is strictly fetal origin, endothelial cells in our dataset is of mixed origin as could be identified by comparing Fig. 1b and Fig. 1c. Single cell origin inference was performed using RNA-expression-similarity to single cells from external dataset which were identified to be of maternal or fetal origin by SNP. The results were shown in Supp. Fig. 2, which identifies a large fraction of endothelial cells of maternal origin.*

Also, the authors might like to consider repeating the analysis using external scRNA datasets of PE and controls at term (and/or preterm) to see whether the patterns are reproducible?

*Reply: We thank the reviewer for the suggestion. We have followed the reviewer's instruction, and performed validation experiments using external scRNA datasets.*

*By searching for publicly available dataset on NCBI GEO using the keywords "preeclampsia placenta single cell RNA", we obtained data from two different datasets (GSE173193, GSE182381) [4, 5] that contains (a) scRNA-seq experiment and (b) consists both PE and CTRL placenta. Data were subjected to quality control and cell type classification before further analysis. One dataset (GSE182381) showed very few trophoblast and endothelial cells after QC, and was not followed further due to data quality concerns (please refer to Fig. R13 in the ‘Response letter’ pdf file).*

*The dataset GSE173193 is of better quality as assessed by our QC standard (please refer to Fig. R14 in the ‘Response letter’ pdf file) . Due to high non-endothelial cell contamination in the endothelial cell set which is not removable, we were unable to analyze endothelial single cells in this dataset (please refer to Fig. R15 in the ‘Response letter’ pdf file). We cleared and analyzed the trophoblast cells in this dataset, resulting in well defined TSC, VCT, SCT, and EVT populations (please refer to Fig. R16 in the ‘Response letter’ pdf file). Since raw sequencing data was not provided for the dataset, we were unable to perform RNA velocity analysis on this dataset. However, we were able to perform CytoTRACE analysis, and used the stemness index inferred by CytoTRACE as an index of cell developmental maturity. CytoTRACE result showed significant delay of maturation in PE trophoblasts (Fig. R16c).*

*We ranked the trophoblast cell clusters according to their average developmental maturity, and found that PE cells were generally developmentally delayed. Most significantly affected cell clusters included the terminally differentiated VCT (cluster 3), SCT (cluster 13) and EVT (cluster 6) (Fig. R16e). Counting cell frequency in each cluster showed that PE placenta consist more single cells with lower developmental maturity (Fig. R16f). Hence, the PE trophoblasts were developmentally delayed, as in our own dataset.*

*We further analyzed the relative expression level of gene sets under control of the master transcription factors we identified in Fig. 4. We found that for all but one (NFYA gene set in SCT) cases, master transcription factor controlled genes were expressed in lower levels in PE cells compared to CTRL cells (please refer to Fig. R17 in the ‘Response letter’ pdf file). Hence, the master transcription factor regulated gene network is disrupted in PE trophoblasts, as in our own dataset. We conclude that the patterns observed in our own data is reproducible.*

It is not clear how possible batch effects were resolved, e.g, in-house vs external sources; samples of different locations from the current studies.

*Reply: We thank the reviewer for the question. The scRNA results in Fig. 1, 2, and 7 were subjected to batch effect correction with Harmony. We have made clarification of this in the main text with appropriate link to the Methods section.*

Fig 2e and Fig 2f - it would be great to add distribution curves of latent time and CytoTRACE score along with the x and y-axis.

*Reply: We thank the reviewer for the suggestion. We have accordingly modified the figure as reviewer requested (please refer to Fig. R18 in the ‘Response letter’ pdf file). The density distribution of CytoTRACE and latent time were shown on X- and Y-axis, respectively.*

Fig 2g, as the CTRL and PE groups consist of both term and preterm delivery, this reviewer wondered whether there is any confounding effect by the status of preterm?

*Reply: We thank the reviewer for the question. We have plotted trophoblast latent time from samples of each sampled gestational week (please refer to Fig. R19 in the ‘Response letter’ pdf file).*

*Trophoblasts in CTRL pregnancies could be clearly separated into juvenile or adult population and the majority of juvenile populations in 3rd CTRL placentas enrich in fetal sides (Fig. R19a-b). Comparing CTRL maternal sides and CTRL fetal sides with the same gestational week, trophoblast in maternal sides are more mature than that in fetal sides (Fig. R9b), consistent with the physiology that trophoblast originate from the fetal and undergo maturation and differentiation along its migration to the maternal surface. However, trophoblasts from all PE pregnancies are always stuck in the middle (Fig. R19c). In the comparison groups, when we compared samples from close gestational weeks (29gw CTRL vs 32gw PE; 30gw CTRL vs 35gw PE; 38 gw CTRL vs 36 gw PE), there are always more adult populations in CTRL placentas, though the gestational week of CTRL placenta may be younger than PE (Fig. R19b-c). This data clearly showed that trophoblast maturity increased from early to late CTRL samples, but such transition is blocked in the PE samples. A summary of this result is shown in Fig. R20 in the ‘Response letter’ pdf file.*

Fig 2b and Fig 2e, the CTRL group includes first trimester and stem cells (both external datasets) as well as third trimester samples (current study) - maybe 'juvenile' and 'adult' may represent these?

*Reply: We thank the reviewer for the question. Fig. 2e does not include first trimester cells and iTSC stem cells but only term placentas. The latent time distribution grouped by trimester is shown in Fig. R20. Although the 'day 21' sample consists mostly 'juvenile' population, the 'adult' population increased significantly in early gestation (6-12gw) and stayed at high level in CTRL placenta (Fig. R20).*

Line 361, "The promoter of PEG10 is differentially methylated in the gamete germline (germline differentially methylated region: gDMR) (Acurzio et al., 2021)" and the author showed the promoter region in Fig 6a, which is in the intronic region of PEG10. However, the study from Lux et al. Plos One, 2010 (also referenced from the current study) suggested the promoter would be located in the upstream of transcript start start, rather than within the intronic region of PEG10.

*Reply: We thank the reviewer for the question. We agree with the reviewer that the promoter of PEG10 locates at the 5' upstream of PEG10. From our H3K4me3 and H3K27ac CUT&Tag data, it is also clear that the 5' upstream of PEG10 is associated with higher H3K4me3/H3K27ac signal to be considered as promoter, whilst the intronic region is likely to be an enhancer with higher H3K27ac level. However, the germline differentially methylated region (gDMR) encompassing PEG10 is reported to be large, encompassing several loci on 7q21.3. Since the gDMR is differently defined in different literatures, we searched for literature and took a relatively small one from Hannula-Jouppi, et al. [6] and performed lift-over to hg38 version of genome. A detailed snapshot of the relative position of gDMR, RNA structure, and histone modifications around the SGCE-PEG10 locus is shown in Fig. R21 in the ‘Response letter’ pdf file. We have made clarifications of this point in the manuscript accordingly.*

What are the thresholds being used in 'PE-associated' and "CTRL-associated' cells?

*Reply: We thank the reviewer for the question. Scissor selects cells that are highly associated with certain phenotype. We used the default setting in our Scissor analysis. In binomial fitting model, where significance level (alpha) is 0.05 and percentage cutoff is 0.2 (the default setting, Scissor selected cells should not exceed 20%), the Scissor positive cell is defined as the average of correlations with all bulk samples is greater than 0 and the number of positive correlations is larger than the number of negative correlations, and the Scissor negative cell is defined as the average of correlations with all bulk samples is less than 0 and the number of negative correlations is larger than the number of positive correlations. By Scissor::reliability.test, the p-value of Scissor identified association is 0.000 and AUC is 0.8738095. We have put this explanation into the Methods section. For SCAVENGE, we selected seed cells as the top 0.1% Z-score enriched cells. Cells with top 25% TRS (for either PE or CTRL) were considered positively associated with trait.*

SCT and Endo from Fig S1c do not seem to agree with Fig S4b.

*Reply: We thank the reviewer for the question. The cell frequency in Fig. S1c is the relative frequency of a certain single cell type in an sample, and the cell frequency in Fig. S4b is the relative frequency of PE-associated single cells in this particular cell type. These two frequencies are not directly comparable.*

Line 1242, maybe better to replace "differential genes" with "differentially expressed genes".

*Reply: We thank the reviewer for the suggestion. We have made modifications according to the reviewer's request.*

Also, "pvalue < 0.05" - does it mean the raw p-values or adjusted p-value?

*Reply: We thank the reviewer for the question. This refer to the raw P-values.*

"Absolute value of log2FC > 0.5" - does it mean the original (i.e. unbroken) log2FC or shrunken log2FC?

*Reply: We thank the reviewer for the question. We took the logFC value from DEseq2, which is the original log2FC.*

It would be great to include the QC metrics for all the sequencing data, e.g. sequencing depth, the number of filtered reads etc.

*Reply: We thank the reviewer for the question. We have now provided the raw sequencing QC metrics of each libraries as a supplementary table.*

The selection of their references should be trimmed and they should be more focused. Likewise, the discussion section is too long.

*Reply: We thank the reviewer for the suggestion. We have therefore re-analyzed the references to focus on the most relevant ones. We have re-written the discussion section to trim it.*

Please provide links of e GSA repository (Accession number: HRA001423)

*Reply: We thank the reviewer for the question. We have made the link temporarily available for revision usage at: https://ngdc.cncb.ac.cn/gsa-human/s/4oxbZPvS .*

In Fig S1a - the color code for the data from Yu et al is not distinguishable.

*Reply: We thank the reviewer for the question. Since single cells from Yu et al. paper is relatively few in number, they are not very visible. We have now modified the coloring of Fig. S1a as shown in Fig. R22 in the ‘Response letter’ pdf file to improve visualization of the data. Furthermore, we provided additional, separated visualization of single cells from each dataset as shown in Fig. R22.*

Fig S3a the cell type annotation of scATAC has been covered by that of scRNA

*Reply: We thank the reviewer for pointing out the problem. We have now corrected the figure (please refer to Fig. R23 in the ‘Response letter’ pdf file).*

Very difficult to follow the main figures and their related supplementary figures. For example, page 8, Figure S7, a supplementary figure for Fig 2, is mentioned before Figure S5 and S6.

*Reply: We thank the reviewer for pointing out the problem. We have now re-written the paper to fix the citation orders of figures and tables.*

Fig S6d and S6e, no legend for DC1 and DC2.

*Reply: We thank the reviewer for pointing out the problem. The DC1 and DC2 refer to diffusion component 1 and 2, as eigenvectors in diffusion map analysis. We have made the clarification into manuscript.*

Line 859, no reference given to ACOG guideline.

*Reply: We thank the reviewer for pointing out the problem. We have put the reference [7] into manuscript.*

Page 35, "Public data" - please give the accession numbers of external datasets.

*Reply: We thank the reviewer for pointing out the problem. The used external datasets in the manuscript are: H3K27ac ChIP-seq data of placenta from Roadmap Project: GSM1127147; human embryo ATAC-seq: SRP163205 [8] and GSE101571 [9]; human embryo methylation sequencing: GSE18233 [10]; human placenta single cell RNAseq: PRJNA492324 [11], PRJEB28266 [12], GSE150578 [13] and GSE173193 [4]; human placenta bulk RNAseq GSE148241 [2]; human brain vascular single cells RNAseq GSE187875 [14]. We have put the reference into the methods section.*

Line 909, 915, 962, please provide the model number of Novaseq and the place where it was performed.

*Reply: We thank the reviewer for the question. The model number of Novaseq is Novaseq 6000. Sequencing was performed at Berry Genomics, Beijing, China. ShareSeq data generated during this revision was performed on MGI2000 sequencer in Euler Technology, Beijing, China. We have put the information into manuscript.*

Line 984, which normalisation method was used?

*Reply: We thank the reviewer for the question. We used 'LogNormalize' method in Seurat for normalization. We have put the information into manuscript.*

And line 1040, which adjustment method was used?

*Reply: We thank the reviewer for the question. We used Bonferroni method in R for p-value adjustment. We have put the information into manuscript.*

Line 1017, no reference given to cisTopic

*Reply: We thank the reviewer for the question. We have put the information [15] into manuscript.*

and line 1229, no reference given to eagle.

*Reply: We thank the reviewer for the question. We have put the information [16] into manuscript.*

Line 1047, "pct.1-pct.2" what does that mean?

*Re: We thank the reviewer for the question. Pct.1 is the percentage of cells in the cluster where differential expression of the gene is detected, and pct.2 is the percentage of cells in all other (as comparison control) clusters. 'abs(pct.1 - pct.2) > 0.3' means the difference of cell fraction that positively expressing a certain gene between the given cluster and all other clusters is at least 30%. For example, if 60% of single cells in a cluster X expressed gene Y, compared to only 20% of single cells not in cluster X expressed gene Y, then the abs(pct.1 - pct.2) is 0.4. We have put the information into manuscript.*

Page 46, bulk RNA-Seq data processing is not described in detail. For example, no descriptions of the *options used by STAR for mapping and quantification.*

*Reply: We thank the reviewer for the question. The parameters used in mapping and quantification was as folllowed: alignment was performed with STAR (version 2.5.3a). STAR reference genome was built with '--runMode genomeGenerate --runThreadN 16 --genomeDir b37/STAR-genome --genomeFastaFiles b37/human_g1k_v37_decoy.fasta --sjdbGTFfile b37/Homo_sapiens.GRCh37.82.gtf --sjdbOverhang 100'. Alignment was performed with '--chimSegmentMin 20 --chimScoreMin 5 --quanMode GeneCounts --twopassMode Basic --outSAMtype BAM SortedByCoordinate'. The quantified gene table was then passed to DEseq2 and analyzed with the default parameter, only filtering for genes that are expressed. Expressed genes were filtered as 'count >= 10'. We have put the information into manuscript.*

Also give a full reference for GSE148241.

*Reply: We thank the reviewer for the question. GSE148241 is from Yang X et al. Hypertension. 2020 Jun;75(6):1532-1541. PMID: 32306769 [2]. We have put the information into manuscript.*

It is very unusual not doing multiple corrections for p-values. In Fig 4g and Fig 5a, is the y-axis based on adjusted or unadjusted p-values?

*Reply: We thank the reviewer for the question. We have accordingly modified the figures according to their adjusted p-values, as shown in Fig. R24 in the ‘Response letter’ pdf file. In Fig. 4g, the maximal value of adjusted p-value of labelled gene is 3.403427e-05. In Fig. 5a, the maximal value of adjusted p-value of labelled gene is 5.100295e-08. We have updated all differential gene expression figures with adjusted p-value. These updated figures were now put into the manuscript to replace the unadjusted ones.*

Fig S17 - neither coordinates nor the transcript architecture provided for PEG10

*Reply: We thank the reviewer for the question. We have now corrected the figure to include the transcript architecture as well as the coordinates (please refer to Fig. R25 in the ‘Response letter’ pdf file).*

*References for Reviewer 2*

*1. Opichka MA, Rappelt MW, Gutterman DD, Grobe JL, McIntosh JJ: Vascular Dysfunction in Preeclampsia. Cells 2021, 10.*

*2. Yang X, Yang J, Liang X, Chen Q, Jiang S, Liu H, Gao Y, Ren Z, Shi YW, Li S, et al: Landscape of Dysregulated Placental RNA Editing Associated With Preeclampsia. Hypertension 2020, 75:1532-1541.*

*3. Schupp JC, Adams TS, Cosme C, Jr., Raredon MSB, Yuan Y, Omote N, Poli S, Chioccioli M, Rose KA, Manning EP, et al: Integrated Single-Cell Atlas of Endothelial Cells of the Human Lung. Circulation 2021, 144:286-302.*

*4. Zhou W, Wang H, Yang Y, Guo F, Yu B, Su Z: Trophoblast Cell Subtypes and Dysfunction in the Placenta of Individuals with Preeclampsia Revealed by Single‑Cell RNA Sequencing. Mol Cells 2022, 45:317-328.*

*5. Campbell KA, Colacino JA, Puttabyatappa M, Dou JF, Elkin ER, Hammoud SS, Domino SE, Dolinoy DC, Goodrich JM, Loch-Caruso R, et al: Placental cell type deconvolution reveals that cell proportions drive preeclampsia gene expression differences. Commun Biol 2023, 6:264.*

*6. Hannula-Jouppi K, Muurinen M, Lipsanen-Nyman M, Reinius LE, Ezer S, Greco D, Kere J: Differentially methylated regions in maternal and paternal uniparental disomy for chromosome 7. Epigenetics 2014, 9:351-365.*

*7. Gestational Hypertension and Preeclampsia: ACOG Practice Bulletin Summary, Number 222. Obstet Gynecol 2020, 135:1492-1495.*

*8. Liu L, Leng L, Liu C, Lu C, Yuan Y, Wu L, Gong F, Zhang S, Wei X, Wang M, et al: An integrated chromatin accessibility and transcriptome landscape of human pre-implantation embryos. Nat Commun 2019, 10:364.*

*9. Wu J, Xu J, Liu B, Yao G, Wang P, Lin Z, Huang B, Wang X, Li T, Shi S, et al: Chromatin analysis in human early development reveals epigenetic transition during ZGA. Nature 2018, 557:256-260.*

*10. Zhu P, Guo H, Ren Y, Hou Y, Dong J, Li R, Lian Y, Fan X, Hu B, Gao Y, et al: Single-cell DNA methylome sequencing of human preimplantation embryos. Nat Genet 2018, 50:12-19.*

*11. Suryawanshi H, Morozov P, Straus A, Sahasrabudhe N, Max KEA, Garzia A, Kustagi M, Tuschl T, Williams Z: A single-cell survey of the human first-trimester placenta and decidua. Sci Adv 2018, 4:eaau4788.*

*12. Vento-Tormo R, Efremova M, Botting RA, Turco MY, Vento-Tormo M, Meyer KB, Park JE, Stephenson E, Polanski K, Goncalves A, et al: Single-cell reconstruction of the early maternal-fetal interface in humans. Nature 2018, 563:347-353.*

*13. Yu L, Wei Y, Duan J, Schmitz DA, Sakurai M, Wang L, Wang K, Zhao S, Hon GC, Wu J: Blastocyst-like structures generated from human pluripotent stem cells. Nature 2021, 591:620-626.*

*14. Winkler EA, Kim CN, Ross JM, Garcia JH, Gil E, Oh I, Chen LQ, Wu D, Catapano JS, Raygor K, et al: A single-cell atlas of the normal and malformed human brain vasculature. Science 2022, 375:eabi7377.*

*15. Bravo Gonzalez-Blas C, Minnoye L, Papasokrati D, Aibar S, Hulselmans G, Christiaens V, Davie K, Wouters J, Aerts S: cisTopic: cis-regulatory topic modeling on single-cell ATAC-seq data. Nat Methods 2019, 16:397-400.*

*16. Loh PR, Danecek P, Palamara PF, Fuchsberger C, Y AR, H KF, Schoenherr S, Forer L, McCarthy S, Abecasis GR, et al: Reference-based phasing using the Haplotype Reference Consortium panel. Nat Genet 2016, 48:1443-1448*

**Second round of review**

**Reviewer 1**

Thanks for the authors' revision. The following points need to be clarified：

1. According to this manuscript, the authors showed that PE placentas retain more immature trophoblast cells with delayed epigenetic reprogramming, especially de novo DNA methylation. While, the exact roles of DNA methyltransferases or demethylases in PE and the potential mechanisms underlying abnormal DNA methylation have not been studied.

2. Although the authors described that the frequency of stromal cells, including "Endo" and "Fibro" types, in PE placenta samples is higher than that of CTRL placenta samples, there was no significant difference in the number of stromal cells in PE and CTRL placenta samples in Fig. 1D. So, at first, the authors should label stromal cells containing "Endo" and "Fibro" types clearly. Secondly, the authors should label the number of stromal cells in PE and CTRL placenta samples in Fig. 1D.

3. Thanks for the more wet experiments about PEG10. While, gain-of-function or loss-of-function of PEG10 in HUVECs, not in trophoblast cells. On the other hand, the authors should perform the knockdown experiment of PEG10 in trophoblast cells of PE samples or overexpression experiment of PEG10 in trophoblast cells of CTRL samples, and then detect the maternal vascular development to confirm the important roles in PE.

4. Some previous publications report the elevated expression of PEG10 in PE placentas（Liang X., et al, Genet Mol Res, 2014; Chen H., et al, Acta Histochem, 2012）. PEG10 is required for the maintenance of fetal capillary structure in the mouse placenta (Shiura H., et al, Development, 2021). The conclusion of this manuscript is partially similar to these previous publications, though they used a number of multi-omics data and different computational tools.

5. In this manuscript, the authors display many bioinformation analysis and presented figures. Can the author appropriately reduce some data and present the most critical data to enhance logicality and readability of this manuscript?

**Reviewer 2**

This reviewer would like to appreciate the authors for getting back with their rebuttal. Previously it was mainly argued that 1) their claims were not supported with multiple lines of evidence, and 2) the manuscript appeared very complex in terms of the structure. The first point seems to be addressed by conducting more experiments (e.g. H3K28me3 Cut&Tag and cell culture), but the second point still remains to be addressed. It was recommended previously to focus on either one of their claims (i.e., developmental delay or PEG10 in vascular development), but the authors seem to prefer keeping them both, which could be part of the reason for the second point. If the authors would insist on that direction, probably they would better significantly slim down on the developmental delay and early transcriptional defect part, while being less speculative.

Other comments

1. The result section should focus on describing their findings while remaining less subjective. There are some sentences in Result, which should be removed or moved to Discussion, e.g. line 165-173 (page 8)

2. In other studies, there seemed contradictory results about PEG10 expression level in PE, which is reported being downregulated in PE (e.g. Baird et al. 2023 (pmid: 37464405), Liang et al. 2014 (pmid: 25526181)), or no change (Gong et al. 2021 - https://www.obgyn.cam.ac.uk/placentome/). The authors might want investigate this from other publicly available datasets, including GSE173193 which was already included in the manuscript.

3. In the volcano plots, the location of H19 appeared opposite in Fig 4g and Suppl Fig 22. Also, the order of the most significant gene (i.e. located on top) does not seem to agree in both. For example, GNAS and PEG10 (both >200 on the y-axis) are the top two in Fig 4g, but they are not ranked as top Suppl Fig 22 and even their scale on the y-axis <90 at most.

4. Two different versions of human genome reference were used in the analysis (line 1250 page 46) – why?

5. The x-axis is shown with CytoTrace score in Suppl Fig 11d, whereas it is shown as “latent time” in Fig 2, Suppl Fig 9 and 10. That said, both PE and CTRL appeared bimodal in Suppl Fig 11d.

6. Line 430-431 (page 18),the PEG10 promoter region should be reinvestigated. The authors implied that the peaks from H3K4me3 and H3K27ac (Suppl Fig 27a) indicate the promoter region, but clearly it is far from the TSS and it is located between the 1st and the 2nd exon. Please refer to the study from Ahn et al. 2022 (pmid: 32878957) and Suzuki et al. 2007 (17432937).

**Authors Response**

**Point-by-point responses to the reviewers’ comments:**

Reviewer #1: Thanks for the authors' revision. The following points need to be clarified：

1. According to this manuscript, the authors showed that PE placentas retain more immature trophoblast cells with delayed epigenetic reprogramming, especially de novo DNA methylation. While, the exact roles of DNA methyltransferases or demethylases in PE and the potential mechanisms underlying abnormal DNA methylation have not been studied.

*Reply: We thank the reviewer for the comment. The current study aims to provide a comprehensive single cell atlas of preeclampsia for identifying the associated molecular mechanism. We identified multiple abnormalities in preeclampsia including the impaired epigenetic reprogramming, delayed trophoblast maturation, and PEG10 overexpression. Whilst the exact roles of DNA methyltransferases or demethylases in PE, or their underlying molecular mechanisms, have not been addressed in this work, we consider it is more suitable to address these questions in future studies.*

2. Although the authors described that the frequency of stromal cells, including "Endo" and "Fibro" types, in PE placenta samples is higher than that of CTRL placenta samples, there was no significant difference in the number of stromal cells in PE and CTRL placenta samples in Fig. 1D. So, at first, the authors should label stromal cells containing "Endo" and "Fibro" types clearly. Secondly, the authors should label the number of stromal cells in PE and CTRL placenta samples in Fig. 1D.

*Reply: We thank the reviewer for the question. We have labeled 'Fibro' (fibroblasts) and 'Endo' (endothelia) to provide the specific cell counts from either PE or CTRL samples in each cell type (Fig. R1). To do this, we analyzed all single-cell RNAseq samples including in-house sequenced term placentas, early gestational week placenta, and iTSC from public sources (GSE150578, PRJEB28266 and PRJNA492324). The number of endothelial cells and fibroblast from CTRL or PE placentas are listed in Table R1 (current Suppl. Table 2 "Cell number in Fig. 1d" sheet).*

*In Supp. Fig. 1d, we computed "cell type frequency" by dividing single cell number from a given cell type to the total cell count from that sample, then compared cell type prevalence between CTRL term placentas and PE term placentas. The detail numbers corresponding to 'Fibro' and 'Endo' cell types are listed in Table R2. The mean frequency of endothelia (Endo.) is 0.0635 in CTRL and 0.124 in PE (Wilcoxon test P = 0.8), while for fibroblasts (Fibro.), it is 0.0948 in CTRL and 0.151 in PE (Wilcoxon test P = 0.4). Overall, the stromal cell prevalence seems to be increased in PE placenta, but not significant.*

*We have updated the annotation in Fig. 1d. and included this source data in the revised supplementary table (Suppl. Table 2 "Cell number in Fig. 1d" sheet) .*

3. Thanks for the more wet experiments about PEG10. While, gain-of-function or loss-of-function of PEG10 in HUVECs, not in trophoblast cells. On the other hand, the authors should perform the knockdown experiment of PEG10 in trophoblast cells of PE samples or overexpression experiment of PEG10 in trophoblast cells of CTRL samples, and then detect the maternal vascular development to confirm the important roles in PE.

*Reply: We thank the reviewer for the comment. In our hypothesis, PEG10 functions in endothelial cells. Hence, we tested their function in HUVEC cells, which is a stable and robust cell-line representing vascular endothelial system. PEG10 is reported to shuttle from the "source" (PEG10-synthesizing) cell to a "target" (PEG10-null) cell [1]. Even in an artificial system which does not express PEG10 originally, forced expression of mammalian PEG10 results in shuttling of PEG10 protein and its bound RNA to the "target" cells. Hence, we do not think over-expressing PEG10 in trophoblast for its transfer to endothelial cells is necessary. Testing PEG10 in HUVEC cells is sufficient to validate the hypothesis and reveal molecular function of PEG10 in PE.*

*Preeclampsia is a human-specific phenotype [2, 3]. In rodent models, preeclampsia could be induced by surgically reduced uterine perfusion [4-6], hypoxia environmental stress [7-9], diet [10, 11], chemical [12], administration of sFlt-1 [13-15], N-nitro-L-arginine methyl ester [16, 17], overexpression of sFlt-1 [18, 19], human STOX1 [20], human renin and angiotensinogen [21, 22], genetic deficiency in catechol-O-methyltransferase [23]. These animal models are induced during pregnancy and affect all experimental animals, on the contrary, PE occurs spontaneously affecting about 5% of pregnancy wordwide with heterogeneous phenotypes [24-26]. The inbred BPH/5 mouse [27], SHHF/Mcc-fa(cp) rat [28] and Dahl salt-sensitive rat [29] developed hypertension before pregnancy, which are similar to superimposed preeclampsia. Thus, current rodent preeclampsia models are not similar to human early-onset, severe preeclampsia, which is associated with developmentally delayed placenta. Hence, at the moment, the experiment asked by the reviewer could only be performed in vivo in pregnant human, which would be in conflict to the declaration of Helsinki and local legal regulations on human research. We consider it would be an interesting experiment for future studies, if an ex vivo human placentation model is available. However, it is way beyond the scope of this work.*

4. Some previous publications report the elevated expression of PEG10 in PE placentas（Liang X., et al, Genet Mol Res, 2014; Chen H., et al, Acta Histochem, 2012）. PEG10 is required for the maintenance of fetal capillary structure in the mouse placenta (Shiura H., et al, Development, 2021). The conclusion of this manuscript is partially similar to these previous publications, though they used a number of multi-omics data and different computational tools.

*Reply: We thank the reviewer for the comment. The expression of PEG10 in PE placentas has been reported in several prior studies. The Chen et al. [30] and Liang X et al. [31] reported association between PEG10 expression and PE in placentas, without identifying the cellular origin of this molecular alteration. However, whether the increased PEG10 was a cause or a consequence of PE remained unclear. Furthermore, the pathological consequence of increased PEG10 in PE placentas is elusive. In our study, we found significantly increased single-cell PEG10 RNA expression in PE trophoblast (Fig. 5a-c) and endothelial cells (Fig. 6e). This is supported by immunofluorescence staining in human placentas (Fig. 5e-h), single-cell ATAC analysis, and histone modification by Cut&Tag (Fig. 6a). We further tracked a fetus-carried paternal PEG10 SNV in a PE sample to show that the PEG10 transcripts in maternal cells were originated from the fetal cells (Fig. 6). Finally, we have performed experiments to show that excessive PEG10 impairs endothelial cell development and function by perturbing the TGF-beta signalling pathway (Fig. 9). Overall, our data provided evidence of excessive expression of PEG10 in PE trophoblast impairs vascular development in their adjacent endothelial cells. These results improved our understanding of PE pathology and revealed new targets of clinical intervention.*

5. In this manuscript, the authors display many bioinformation analysis and presented figures. Can the author appropriately reduce some data and present the most critical data to enhance logicality and readability of this manuscript?

*Reply: We thank the reviewer for the comment. We have re-written the manuscript to improve its logic flow and readability.*

Detailed response to reviewer #2

Reviewer #2:

This reviewer would like to appreciate the authors for getting back with their rebuttal. Previously it was mainly argued that 1) their claims were not supported with multiple lines of evidence, and 2) the manuscript appeared very complex in terms of the structure. The first point seems to be addressed by conducting more experiments (e.g. H3K28me3 Cut&Tag and cell culture), but the second point still remains to be addressed. It was recommended previously to focus on either one of their claims (i.e., developmental delay or PEG10 in vascular development), but the authors seem to prefer keeping them both, which could be part of the reason for the second point. If the authors would insist on that direction, probably they would better significantly slim down on the developmental delay and early transcriptional defect part, while being less speculative.

*Reply: We thank the reviewer for acknowledging that our conclusion is now strengthened by adding the H3K27me3 CUT&Tag and cell culture experiments. For the second point, we thank the reviewer for pointing out that it would be better to slim down on the developmental delay part, and tune down the overall tone of paper. Since the developmental delay part serves as a basis for PEG10 overexpression in PE trophoblasts, we consider it might be better to keep it in paper. We have now re-written the paper to reduce the discussion on developmental delay and transcriptional defect in PE placenta, as the reviewer requested.*

Other comments

1. The result section should focus on describing their findings while remaining less subjective. There are some sentences in Result, which should be removed or moved to Discussion, e.g. line 165-173 (page 8)

*Reply: We thank the reviewer for the comment. We have slimed down the developmental delay and transcription defect parts and removed subjective sentences in the Result part.*

2. In other studies, there seemed contradictory results about PEG10 expression level in PE, which is reported being downregulated in PE (e.g. Baird et al. 2023 (pmid: 37464405), Liang et al. 2014 (pmid: 25526181)), or no change (Gong et al. 2021 - https://www.obgyn.cam.ac.uk/placentome/). The authors might want investigate this from other publicly available datasets, including GSE173193 which was already included in the manuscript.

*Reply: We thank the reviewer for the question. As the reviewer asked, we re-evaluated multiple external publicly accessible datasets for analyzing RNA expression levels of PEG10 in PE and CTRL placentas (Table R3). These data are from different experimental platforms (RNA microarray and next-generation sequencing), and consists samples from different ethnicities. Our re-analysis results show that significantly increased PEG10 in PE could be detected in 4 (GSE190971, GSE234729, GSE30186, and GSE173193, Table R3) out of 10 different experiments. On the other hand, in all these experiments, we only detect PEG10 decrease in one dataset (GSE75010), with a tiny effect size (logFC = -0.00876, Table R3). Thus, we detected more increased PEG10 expression in PE in these datasets.*

*Further analysis showed that the relative expression of PEG10 in PE placentas is influenced by both demographic feature and tropholast subtype composition in placentas. In dataset GSE234729 (a bulk RNAseq dataset), we observed that the PE-specific increase of PEG10 is associated with ethnicity (Fig. R2). In the Asian mothers, PE placentas exhibit statistically significantly higher PEG10 expression (Fig. R2). Whilst PEG10 is still increased in PE samples from European and African mothers, the differences are not significant (Fig. R2).*

*Whilst this result agrees with our findings, it also suggests the complexity of the molecular phenotypes and pathological mechanisms of preeclampsia. It is known that PE etiology differs between maternal ethnicities [1-4]. In concordance to this, the expression of PE-specific genes (LEP, FSTL3, FLT1, HK2 etc., https://www.medrxiv.org/content/10.1101/2023.06.16.23291528v1) differs significantly between pregnant females of Asian or African/European ancestries (https://www.medrxiv.org/content/10.1101/2023.06.16.23291528v1).*

*The inconsistent observation of PEG10 overexpression in PE samples might be addressed to such difference in disease etiology.*

*As the reviewer requested, we separately analyzed the GSE173193 scRNA dataset and our in-house scRNA dataset. Increased PEG10 expression in the terminally differentiated SCT and EVT trophoblast cells in PE samples was found in both datasets (Fig. R3 and R4).*

*Whilst these results agree with our original observation from full dataset analysis, they also suggest that cell type composition might influence the power to detect PEG10 expression difference in bulk RNAseq/microarray experiments. Particularly, the sampled placenta might differ in the early (VCTp/VCT)-to-late (SCT/EVT) trophoblast ratio. For example, sampling the maternal surface of placenta would result in increased SCT/EVT prevalence in the sample, whilst sampling the fetal surface of placenta would obtain more VCTp/VCT. Hence, the failure to detect increased PEG10 expression in PE samples from some of the experiments in Table R3 may also result from a sampling bias.*

*We have now put the dataset-wise analysis of single-cell RNAseq results in the Results section. Because bulk RNA-seq analysis involved some publicly accessible data not yet published in peer-reviewed journals (for example, GSE234729), we consider it might better to leave these results for revision only.*

3. In the volcano plots, the location of H19 appeared opposite in Fig 4g and Suppl Fig 22. Also, the order of the most significant gene (i.e. located on top) does not seem to agree in both. For example, GNAS and PEG10 (both >200 on the y-axis) are the top two in Fig 4g, but they are not ranked as top Suppl Fig 22 and even their scale on the y-axis <90 at most.

*Reply: We thank the reviewer for the question. In Fig. 4g, we calculated differential expression of an imprinted gene list between CTRL and PE trophoblasts, including VCTp, VCT, SCT and EVT subtypes, while in Supp. Fig. 22, we conducted a separate differential expression analysis for each trophoblast subtype, comparing PE and CTRL groups independently. In differential expression analysis, Wilcox test was used. The difference in trophoblast subtype composition between Fig. 4g and Supp. Fig. 22 accounts for the observed differences in the volcano plots. The trophoblast population and analysis methods are identical in Fig.4g and Fig.5a, thus the position of overlapped genes in Fig. 4g and Fig. 5a are comparable, and we re-labeled the imprinted genes from Fig. 4g in Fig. 5a for clearer comparsion (Fig. R5). Additionally, we have compiled a list of differentially expressed genes from Fig. 4g, Fig. 5a and Supp. Fig. 22 to Supp. Table S8.*

4. Two different versions of human genome reference were used in the analysis (line 1250 page 46) – why?

*Reply: We thank the reviewer for the question. We upgraded our ATAC, Cut&Tag and Cut&Run analysis pipeline at 2021 and updated the human reference genome to hg38. Prior to or during 2020, ATAC, Cut&Tag and Cut&Run analysis were based on the hg37 reference genome, while from 2021 onwards, we have been utilizing hg38 as reference. Our previous preprint contained some of the prior-to-2020 results shown in this paper (https://www.biorxiv.org/content/10.1101/2020.05.08.085290v3). Hence, we consider it might be better to leave them as they were to keep the consistency.*

5. The x-axis is shown with CytoTrace score in Suppl Fig 11d, whereas it is shown as “latent time” in Fig 2, Suppl Fig 9 and 10. That said, both PE and CTRL appeared bimodal in Suppl Fig 11d.

*Reply: We thank the reviewer for the question. According to the methods used in scVelo, the latent time is indicative of developmental age [5]. It is inferred from both spliced and unspliced RNA read count matrices. An increase in latent time corresponds to advanced developmental age. On the other hand, the CytoTrace score estimates the stemness of a single cell [6]. It is calculated from spliced RNA read count matrix. A lower CytoTrace score means terminally differentiated cells with decreased stem potential. Theoretically, as cell becomes more mature, the latent time should increase, and the CytoTrace score should decrease.*

*Unspliced count matrix was not provided in dataset GSE173193 (Supp. Fig. 11), prohibiting us to perform direct one-to-one comparison of the dataset GSE173193 to our in-house dataset with fully similar analysis methods. Because we previously observed inverse-correlation of CytoTrace score and latent time (Fig. 2e and 2f), we assessed trophoblast maturation in GSE173193 with CytoTrace analysis.*

*Here, to clarify the reviewer's concern, we performed exactly similar CytoTRACE analysis on the in-house dataset and the external dataset GSE173193, and directly compared their results head-to-head.*

*We re-analyzed CytoTrace score from the trophoblast of in-house sequenced trophoblast in Fig.2b and decomposed the CytoTrace scores into bimodal normal distributions with mixtools in CTRL and PE, respectively. As the reviewer pointed out, the distribution patterns of CytoTrace scores in both PE and CTRL exhibit a bimodal mode, with Adult and Juvenile distributions (Fig. R6a-b).*

*The CytoTRACE score distribution of CTRL Adult trophoblast is significantly different from that of PE Adult (p-value < 2.2e-16, Kolmogorov-Smirnov test). Similar situation was observed when comparing the CytoTRACE score distributions of CTRL Juvenile and PE Juvenile trophoblasts (p-value < 2.2e-16, Kolmogorov-Smirnov test). Furthermore, PE trophoblasts have significantly higher CytoTrace score in both Adult and Juvenile categories (Fig. R6c), indicating reduced maturity of PE trophoblast in both PE Adult and PE Juvenile distributions. Thus, though both CTRL and PE trophoblast exhibited bimodal distributions in CytoTrace score, PE trophoblast showed developmental delay. We further classified the CTRL and PE trophoblast according to which distribution they are most likely to belong (CTRL Adult, PE Adult, CTRL Juvenile and PE Juvenile) (Fig. R6d) and calculated the relative prevalence of single cells of each class in CTRL and PE (Fig. R6e). Trophoblasts from CTRL were significantly enriched in the CTRL Adult class (p-value < 2.2e-16, CTRL vs PE odds ratio = 5.43, Fisher's Exact Test, Fig. R6e), however, trophoblasts from PE were significantly enriched in PE Juvenile class (p-value < 2.2e-16, CTRL vs PE odds ratio = 0.35, Fisher's Exact Test, Fig. R6e). These results are reproduced in the external validation GSE173193 dataset (Fig. R7) with exactly similar codes. Hence, our original observations that PE trophoblasts are developmentally delayed could be supported by additional external dataset.*

6. Line 430-431 (page 18),the PEG10 promoter region should be reinvestigated. The authors implied that the peaks from H3K4me3 and H3K27ac (Suppl Fig 27a) indicate the promoter region, but clearly it is far from the TSS and it is located between the 1st and the 2nd exon. Please refer to the study from Ahn et al. 2022 (pmid: 32878957) and Suzuki et al. 2007 (17432937).

*Reply: We thank the reviewer for the question. We have zoom-in-ed Supp. Fig. 27b around the PEG10 promoter region in Fig. R7 and we modified Supp. Fig. 27b as Fig. R8 for clarification.*

**Third round of review**

**Reviewer 1**

The authors have addressed all my concerns.

**Reviewer 2**

This reviewer would like to appreciate again the authors for getting back with their rebuttal with regard to my previous comments, which seem to be addressed more or less. However, even though the PEG10 gDMR overlaps the TSS (Fig 6a), the location of the PEG10 promoter region described in the manuscript, between the 1st and the 2nd exon shown as the peak from H3K4me3 and H3K27ac (Suppl Fig 27), is different from what other previous studies reported (e.g. pmid: 32878957, 17432937 and 20084274), which indicate the upstream of the TSS. That said, there could be a possibility of having an alternative promoter region (or it could be an enhancer region) as indicated by the EPD database (https://epd.expasy.org/cgi-bin/epd/get_doc?db=hgEpdNew&format=genome&entry=PEG10_2) and FANTOM – they are indicated by the following GeneCards website: https://www.genecards.org/cgi-bin/carddisp.pl?gene=PEG10#genomic_location. Therefore, to avoid further confusion, this reviewer would suggest simply replacing “promoter” with “locus” in the followings:

1) at line 382, e.g. “scATAC data showed that PEG10 locus was only accessible…”

2) at line 779 and 789 (page 30), e.g. “Compared to trophoblasts, Ec and Fb has little chromatin accessibility on PEG10 locus…” and “The PEG10 locus is subjected to imprinting (gDMR)...”.

**Authors Response**

**Point-by-point responses to the reviewers’ comments:**

Response: We modified the phrases regarding PEG10 locus, exactly as requested by Referee 2.
